# Supplementary material for: Feasibility and benefits of group-based exercise in residential aged care adults: a pilot study for the GrACE programme
Source: PeerJ. 2016 May 18;4:e2018. doi: 10.7717/peerj.2018 (PMC4878364; doi:10.7717/peerj.2018)
Supplement: Supplemental Information 2 [file peerj-04-2018-s002.pdf]

ClinicalTrials.gov PRS
Protocol Registration and Results System

ID: RO 1823 Feasibility and Benefits of Group Based Exercise in Residential Aged Care Adults NCT02640963

Protocol Registration Preview

Feasibility and Benefits of Group Based Exercise in Residential Aged Care Adults

This study has been completed.

Sponsor:
Bond University

Information provided by (Responsible Party):
Samantha Fien, Bond University

ClinicalTrials.gov Identifier:
NCT02640963

First received: December 15, 2015
Last updated: March 18, 2016
Last verified: March 2016

Purpose

This study is a pilot study (feasibility and acceptability study), which will compare feasibility and efficacy outcomes between a 12-week Exercise Program and control group in RAC residents.

Condition	Intervention	Phase
Geriatric Disorder<br>Sarcopenia	Exercise<br>Control	N/A

Study Type: Interventional
Study Design: Parallel Assignment, Open Label, Randomized, Safety/Efficacy Study

Official Title: Feasibility and Benefits of Group Based Exercise in Residential Aged Care Adults: a Pilot Study for the GrACE Programme

Further study details as provided by Samantha Fien, Bond University:

Primary Outcome Measure:

- recruitment rate [Time Frame: up to 12 weeks] [Designated as safety issue: No]

  defined as the number of residents recruited from those invited. Measurement units = number and percentage

- measurement (physiological and surveys) completion rate [Time Frame: up to 12 weeks] [Designated as safety issue: No]

  defined as the number of participants able to complete each outcome

measure at baseline and follow-up. Measurement units = number and percentage

- loss-to-follow-up [Time Frame: up to 12 weeks] [Designated as safety issue: No]
  defined as participants who withdrew or dropped out and did not consent to a follow up assessment. Measurement units = number and percentage

- exercise session adherence [Time Frame: up to 12 weeks] [Designated as safety issue: No]
  measured by the number of sessions attended out of the maximum 24 sessions. Measurement units = number and percentage

- acceptability [Time Frame: up to 12 weeks] [Designated as safety issue: No]
  measured via a programme satisfaction survey completed post-training that assessed the burden of training and testing, as well as how participants felt about the trial. Measurement units = number and percentage

- adverse events [Time Frame: up to 12 weeks] [Designated as safety issue: No]
  defined as incidents in which harm or damage resulted to a participant and included, but were not limited to, falls and fall-related injuries, musculoskeletal or cardiovascular incidents and problems with medication and medical devices. Measurement units = number and percentage

Secondary Outcome Measures:

- Gait Speed [Time Frame: change from baseline to 12 weeks] [Designated as safety issue: Yes]
  Gait speed was recorded via the GaitMat II system (Manufacturer is EQInc; Model is GaitMat II), which required participants to walk across a level pressure mat system 3.66 m (11.91 ft.) long (McDonough et al. 2001). Participants completed the trials at their preferred (habitual) walking (gait) speed. The following instructions were given, "Walk towards the end of the room at a pace that is comfortable for you". Participants were allowed to walk in their own footwear. All measures were initiated from a standing start 2 m (6.56 ft.) from the GaitMat II platform as suggested by Kressig and Beauchet (Kressig & Beauchet 2006) to reduce the effect that acceleration may have on gait speed. The average gait speed (m/s) from three attempts was used for data analysis. Participants were allowed as much rest as required between attempts, with rest periods typically being up to 1 minute. Measurement units = metres per second

- Handgrip strength [Time Frame: change from baseline to 12 weeks] [Designated as safety issue: Yes]

Upper body muscle function was measured by isometric handgrip strength and sit-to-stand performance, respectively. When performing the handgrip strength assessments, participants were seated, instructed to keep their elbow at 90° and asked to squeeze a handgrip dynamometer (Sammons Preston Roylan, Bolingbrook, IL) to their maximum ability for a period of up to five seconds (Mathiowetz 2002). Three trials were performed with the subject's dominant hand with one-minute rest between trials and the best result used for analysis (Roberts 2011). Measurement units = kilograms

- Sit to stand performance [Time Frame: change from baseline to 12 weeks] [Designated as safety issue: Yes]
  In the sit-to-stand measure, participants sat and stood to a full standing position from a chair as many times as possible in 30 seconds whilst keeping their arms crossed against their chest (Millor 2013). Measurement units = repetitions in 30 seconds

Enrollment: 37
Study Start Date: January 2015
Study Completion Date: May 2015
Primary Completion Date: May 2015

Arms	Assigned Interventions
Experimental: Intervention: the GrACE programme<br>The programme included several weight-bearing exercises (using body weight and dumbbells) and a range of seated, non-resisted upper- and lower-body dynamic and reaching movements. While developed for respite care older adults, the GrACE programme was slightly modified for the RAC setting; using reduced range of motion and resistance, and an extended conditioning/familiarisation phase. The conditioning phase lasted for three weeks and focus on the development of correct technique. After concluding the conditioning phase, participants started to use light dumbbells. Participants performed the exercises twice per week for 12 weeks. Training sessions lasted approximately 45 minutes, were separated by at least 48 hours and were delivered by an experienced allied health professional.	Exercise<br>to determine the feasibility of the GrACE (Group Aged Care Exercise) programme in RAC, with the secondary objective of measuring the programme benefits on gait speed, sit to stand and handgrip strength.
Placebo Comparator: Control Group<br>All subjects assigned to the control group were given the option to engage in other activities that were offered by the facility during the 12-week intervention period. Activities were facility	Control<br>to compare with the GrACE (Group Aged Care Exercise)

specific, and included Zumba aerobic exercise and walking, however no specific resistance exercises were offered.	programme in RAC, as well as the secondary objective of measuring the programme benefits on gait speed, sit to stand and handgrip strength against the intervention group

Study Design and Recruitment This study compared the delivery feasibility and outcomes of a 12-week combined resistance and weight bearing exercise programme which the investigators named the GrACE programme. Participant recruitment and assessment occurred over a five-month period.

The RAC was approached about participation via email and telephone follow-up. Potential participants were identified at a meeting with the facility Service Manager. Participants were screened via the inclusion criteria at the meeting with the Service Manager and a Registered Nurse, and written consent was attained prior to participation. Following an explanation of the procedures, purposes, benefits and associated risks of the study, participants had the opportunity to ask questions. A total of 37 older RAC adults provided written informed consent for the study. The exercise group contained 20 participants and the control group 17 participants. Ethical approval to conduct this study was attained from Bond University's Human Ethics Research Committee (RO 1823).

Intervention: the GrACE programme Previous work by our group trialled a successful exercise programme in respite day care that could promise benefits to those in RAC (HenwoodWooding & de Souza 2013). In brief, the GrACE programme included a number of targeted weight-bearing exercises (using body weight and dumbbells) and a range of seated, non-resisted upper- and lower-body dynamic and reaching movements. While developed for respite care older adults, the programme was slightly modified for the RAC setting; initially using reduced range of motion and resistance, and an extended conditioning/familiarisation phase. The conditioning phase lasted for three weeks in which technique was emphasised without using any weights or additional resistance. The focus of this technique of the conditioning phase was to develop the correct technique and minimise the potential for any delayed onset muscle soreness or adverse effects. After concluding the conditioning phase, participants were able to use light dumbbells (often starting with 0.5kg) increasing to heavier dumbbells (up to 4kg) with their increasing capacity over the course of the programme. Participants performed the exercises twice per week for 12 weeks. Training sessions lasted approximately 45 minutes, were separated by at least 48 hours and were delivered by an allied health professional experienced working with RAC adults.

Control Group All subjects assigned to the control group were given the option to engage in other activities that were offered by the facility during the 12-week intervention period. Activities were facility specific, and included Zumba Gold aerobic exercise and walking, however no specific resistance exercises were offered.

Data Collection Reasons for refusal (non-consent) to participate were recorded (Henwood 2014). All muscle function outcome measures in this study have been previously validated for use with older adults, and their protocols reported elsewhere (Henwood, Wooding & de Souza 2013; Sterke et al. 2012). Assessments were completed one-on-one with each participant. During muscle function measures assessments, participants were encouraged to rest as needed and given verbal support and encouragement to reduce any potential burden to the participant.

Eligibility

Ages Eligible for Study: 65 Years to 100 Years
Genders Eligible for Study: Both
Accepts healthy volunteers.

Inclusion Criteria:

- Aged 65 years and over,
- Residing in a RAC,
- Able to walk with a walker and/or walking stick or could self-ambulate and,
- Could provide informed consent.

Exclusion Criteria:

- End-stage terminal and/or life expectancy <6-months (ethical reasons),
- Two person transfer or unable to self-ambulate (due to increased falls risk),
- Unable to communicate or follow instructions (personal needs beyond the scope of this project),
- Insufficient cognitive function to provide informed consent and,
- Dangerous behaviours that would endanger the client or research staff.

Contacts and Locations

Investigators

Study Chair: Justin Keogh, PhD Bond University

More Information

study involving community dwelling older adults

Publications:

Henwood TR, Keogh JW, Reid N, Jordan W, Senior HE. Erratum to: Assessing sarcopenic prevalence and risk factors in residential aged care: methodology and feasibility. J Cachexia Sarcopenia Muscle. 2014 Sep;5(3):237. doi: 10.1007/s13539-014-0151-0.

McDonough AL, Batavia M, Chen FC, Kwon S, Ziai J. The validity and reliability of the GAITRite system's measurements: A preliminary evaluation. Arch Phys Med Rehabil. 2001 Mar;82(3):419-25.

Sterke CS, van Beeck EF, Looman CW, Kressig RW, van der Cammen TJ. An electronic walkway can predict short-term fall risk in nursing home residents with dementia. Gait Posture. 2012 May;36(1):95-101. doi: 10.1016/j.gaitpost.2012.01.012. Epub 2012 Mar 3.

Kressig RW, Beauchet O; European GAITRite Network Group. Guidelines for

clinical applications of spatio-temporal gait analysis in older adults. Aging Clin Exp Res. 2006 Apr;18(2):174-6.

Mathiowetz V. Comparison of Rolyan and Jamar dynamometers for measuring grip strength. Occup Ther Int. 2002;9(3):201-9.

Roberts HC, Denison HJ, Martin HJ, Patel HP, Syddall H, Cooper C, Sayer AA. A review of the measurement of grip strength in clinical and epidemiological studies: towards a standardised approach. Age Ageing. 2011 Jul;40(4):423-9. doi: 10.1093/ageing/afr051. Epub 2011 May 30. Review.

Millor N, Lecumberri P, Gómez M, Martínez-Ramírez A, Izquierdo M. An evaluation of the 30-s chair stand test in older adults: frailty detection based on kinematic parameters from a single inertial unit. J Neuroeng Rehabil. 2013 Aug 1;10:86. doi: 10.1186/1743-0003-10-86.

Henwood T, Wooding A, and de Souza D. 2013. Centre-based exercise delivery: feasability of a staff-delivered program and the benefits for low-functioning older adults accessing respite day care. Activities, Adaptations & Ageing 73:224-238.

Responsible Party:	Samantha Fien, Student, Bond University
Study ID Numbers:	RO 1823
Health Authority:	Australia: Human Research Ethics Committee
Plan to Share Data?:	No
Study Data/Documents:	Study Protocol<br>Activities, Adaptations & Ageing Journal Article Article can be requested via email contact with tim@agefitsolutions.com.au
