# Supplementary material for: Feasibility and benefits of group-based exercise in residential aged care adults: a pilot study for the GrACE programme
Source: PeerJ. 2016 May 18;4:e2018. doi: 10.7717/peerj.2018 (PMC4878364; doi:10.7717/peerj.2018)
Supplement: Supplemental Information 3 [file peerj-04-2018-s003.docx]

## Appendix 1.

The GrACE Programme

The programme included weight-bearing exercises and a range of seated, non-resisted upper- and lower-body dynamic and reaching movements. Prior to performing the exercises, participants warmed up for approximately fifteen minutes using the non-resistive movements, and completed each session with a warm down of approximately ten minutes with targeted stretching. The stretches involved non-resistive movements undertaken two or three times and reaching (stretching) movements were held for 20 seconds. Between the warm up and cool down the participants performed the following-weight bearing and resistance exercises: chair stands, chair dips, calf raises and hip flexor/abdominal lifts, trunk twists, and bicep curl and shoulder press.

The aim was to complete three sets of ten repetitions per exercise. Initially participants worked at a reduced intensity (50% of estimated 1RM) and number of sets and repetitions, with the sets and repetitions increasing over the first four weeks to reach the GrACE programmes full delivery goal. Prior to the GrACE programme all participants were familiarised to the programme, with participants commencing with no resistance so to encourage appropriate technique and posture, whilst reducing reliance on balancing aids before progressing to resistance (dumbbells).


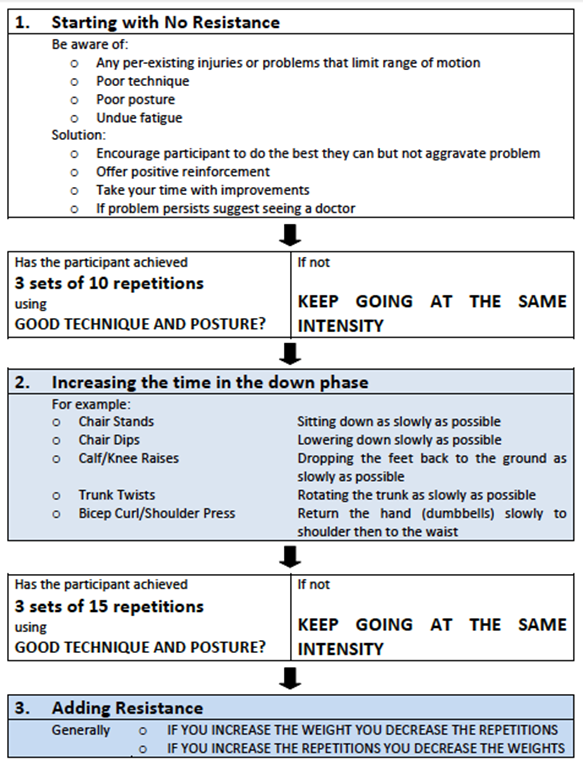


The programme took approximately one hour to complete and was delivered by an accredited, allied health professional. Participants were closely monitored, told to rest as needed and to not over exert themselves.
