# Supplementary material for: Feasibility and benefits of group-based exercise in residential aged care adults: a pilot study for the GrACE programme
Source: PeerJ. 2016 May 18;4:e2018. doi: 10.7717/peerj.2018 (PMC4878364; doi:10.7717/peerj.2018)
Supplement: Supplemental Information 4 [file peerj-04-2018-s004.docx]

## Appendix 2

Feedback from the participants in regards to the exercise group

| **Feedback Questions** | **Comments** |
| --- | --- |
| What was the best aspect of the GrACE programme? | “Neck exercises”  “Improvement”  “The use of weights”  “I liked all of it”  “The instructor”  “Enjoyable group exercise”  “It kept me moving”  “I liked the instructor coming to remind me exercises were on, cause I forgot most of the time”  “Instructor was very concise”  “Nothing was too difficult”  “I enjoyed the challenge”  “The group and the instructor”  “I was very happy with all the programme had to offer”  “Feeling of support”  “Breathing exercises”  “I liked that I got to push myself”  “Everyone was enthusiastic”  “I realised that I need to exercise more, but I have to be realistic”  “Everybody being in a group made the exercises more enjoyable” |
| What aspect of the GrACE programme needs improvement? | “More time for exercises”  “None”  “Nothing”  “None, it was all good”  “I liked it all”  “Needs to go for longer”  “Better spacing of the days we do exercise” |
| Other comments/feedback. | “I like the structure, no interruptions, everyone seems to cope and it is so worthwhile”  “Covered a lot of things that I liked”  “A very good, organised programme – even one could say meticulous”  “I would like the programme to continue”  “It was wonderful”  “Thank you for your organisation and commitment”  “I would like to do more of it”  “I find it easier to walk now after the exercise programme”  “I would like more variation”  “Considering I am 3 months older now than when I commenced, I am surprised that I now feel okay at the end of the programme, I was exhausted at the start of the programme”  “It is a joy to do the exercises with our instructor” |
