# Supplementary material for: Feasibility and benefits of group-based exercise in residential aged care adults: a pilot study for the GrACE programme
Source: PeerJ. 2016 May 18;4:e2018. doi: 10.7717/peerj.2018 (PMC4878364; doi:10.7717/peerj.2018)
Supplement: Supplemental Information 5 [file peerj-04-2018-s005.doc]

GET
  FILE='C:\Users\13133041\Desktop\gait study data spss\gaitwithgsvariablesthisfilesam.sav'.
DATASET NAME DataSet1 WINDOW=FRONT.
CORRELATIONS
  /VARIABLES=meangaitspeed lsteplength rsteplength lstridelength rstridelength lsupportbase rsupportbase lsteptime rsteptime lswingtime rswingtime lstancetime rstancetime lsstime rsstime ldstime rdstime
  /PRINT=TWOTAIL NOSIG
  /STATISTICS DESCRIPTIVES
  /MISSING=PAIRWISE.


Correlations


Notes	
Output Created	18-DEC-2015 15:27:54	
Comments		
Input	Data	C:\Users\13133041\Desktop\gait study data spss\gaitwithgsvariablesthisfilesam.sav	
	Active Dataset	DataSet1	
	Filter	<none>	
	Weight	<none>	
	Split File	<none>	
	N of Rows in Working Data File	101	
Missing Value Handling	Definition of Missing	User-defined missing values are treated as missing.	
	Cases Used	Statistics for each pair of variables are based on all the cases with valid data for that pair.	
Syntax	CORRELATIONS
  /VARIABLES=meangaitspeed lsteplength rsteplength lstridelength rstridelength lsupportbase rsupportbase lsteptime rsteptime lswingtime rswingtime lstancetime rstancetime lsstime rsstime ldstime rdstime
  /PRINT=TWOTAIL NOSIG
  /STATISTICS DESCRIPTIVES
  /MISSING=PAIRWISE.	
Resources	Processor Time	00:00:00.05	
	Elapsed Time	00:00:00.02	


[DataSet1] C:\Users\13133041\Desktop\gait study data spss\gaitwithgsvariablesthisfilesam.sav


Descriptive Statistics	
	Mean	Std. Deviation	N	
meangaitspeed	.6303	.18869	100	
lsteplength	.8756	4.65974	100	
rsteplength	.4123	.08076	100	
lstridelength	.8258	.15412	100	
rstridelength	.8284	.15155	100	
lsupportbase	.1539	.05893	100	
rsupportbase	.1528	.05926	100	
lsteptime	.6542	.11683	100	
rsteptime	.6670	.17462	100	
lswingtime	.7681	3.55937	100	
rswingtime	.4206	.09525	100	
lstancetime	.9391	.27993	100	
rstancetime	.9081	.18487	100	
lsstime	.4178	.09003	100	
rsstime	.4120	.06467	100	
ldstime	.2372	.07225	100	
rdstime	.2385	.07313	100	


Correlations	
	meangaitspeed	lsteplength	rsteplength	lstridelength	rstridelength	lsupportbase	rsupportbase	
meangaitspeed	Pearson Correlation	1	.113	.737**	.742**	.743**	-.200*	-.187	
	Sig. (2-tailed)		.264	.000	.000	.000	.046	.063	
	N	100	100	100	100	100	100	100	
lsteplength	Pearson Correlation	.113	1	.074	.084	.097	.058	.060	
	Sig. (2-tailed)	.264		.464	.403	.335	.568	.556	
	N	100	100	100	100	100	100	100	
rsteplength	Pearson Correlation	.737**	.074	1	.930**	.912**	-.338**	-.356**	
	Sig. (2-tailed)	.000	.464		.000	.000	.001	.000	
	N	100	100	100	100	100	100	100	
lstridelength	Pearson Correlation	.742**	.084	.930**	1	.982**	-.271**	-.273**	
	Sig. (2-tailed)	.000	.403	.000		.000	.006	.006	
	N	100	100	100	100	100	100	100	
rstridelength	Pearson Correlation	.743**	.097	.912**	.982**	1	-.315**	-.314**	
	Sig. (2-tailed)	.000	.335	.000	.000		.001	.001	
	N	100	100	100	100	100	100	100	
lsupportbase	Pearson Correlation	-.200*	.058	-.338**	-.271**	-.315**	1	.953**	
	Sig. (2-tailed)	.046	.568	.001	.006	.001		.000	
	N	100	100	100	100	100	100	100	
rsupportbase	Pearson Correlation	-.187	.060	-.356**	-.273**	-.314**	.953**	1	
	Sig. (2-tailed)	.063	.556	.000	.006	.001	.000		
	N	100	100	100	100	100	100	100	
lsteptime	Pearson Correlation	-.590**	-.099	-.138	-.068	-.040	-.109	-.140	
	Sig. (2-tailed)	.000	.329	.171	.499	.694	.282	.163	
	N	100	100	100	100	100	100	100	
rsteptime	Pearson Correlation	-.570**	-.040	-.108	-.091	-.086	-.131	-.140	
	Sig. (2-tailed)	.000	.696	.283	.369	.394	.195	.165	
	N	100	100	100	100	100	100	100	
lswingtime	Pearson Correlation	.288**	-.007	.260**	.267**	.290**	-.045	-.077	
	Sig. (2-tailed)	.004	.947	.009	.007	.003	.655	.448	
	N	100	100	100	100	100	100	100	
rswingtime	Pearson Correlation	-.289**	.003	.178	.192	.215*	-.510**	-.541**	
	Sig. (2-tailed)	.004	.976	.077	.055	.032	.000	.000	
	N	100	100	100	100	100	100	100	
lstancetime	Pearson Correlation	-.714**	-.060	-.260**	-.220*	-.222*	.059	.078	
	Sig. (2-tailed)	.000	.555	.009	.028	.026	.557	.442	
	N	100	100	100	100	100	100	100	
rstancetime	Pearson Correlation	-.698**	-.100	-.301**	-.238*	-.216*	-.119	-.134	
	Sig. (2-tailed)	.000	.323	.002	.017	.031	.237	.183	
	N	100	100	100	100	100	100	100	
lsstime	Pearson Correlation	-.285**	.006	.154	.173	.206*	-.474**	-.508**	
	Sig. (2-tailed)	.004	.952	.126	.086	.040	.000	.000	
	N	100	100	100	100	100	100	100	
rsstime	Pearson Correlation	-.352**	-.063	-.054	.073	.102	-.194	-.159	
	Sig. (2-tailed)	.000	.536	.590	.470	.311	.053	.114	
	N	100	100	100	100	100	100	100	
ldstime	Pearson Correlation	-.688**	-.086	-.439**	-.435**	-.429**	.130	.109	
	Sig. (2-tailed)	.000	.394	.000	.000	.000	.198	.282	
	N	100	100	100	100	100	100	100	
rdstime	Pearson Correlation	-.667**	-.099	-.388**	-.346**	-.343**	.108	.056	
	Sig. (2-tailed)	.000	.327	.000	.000	.000	.284	.583	
	N	100	100	100	100	100	100	100	

Correlations	
	lsteptime	rsteptime	lswingtime	rswingtime	lstancetime	rstancetime	lsstime	rsstime	
meangaitspeed	Pearson Correlation	-.590**	-.570**	.288**	-.289**	-.714**	-.698**	-.285**	-.352**	
	Sig. (2-tailed)	.000	.000	.004	.004	.000	.000	.004	.000	
	N	100	100	100	100	100	100	100	100	
lsteplength	Pearson Correlation	-.099	-.040	-.007	.003	-.060	-.100	.006	-.063	
	Sig. (2-tailed)	.329	.696	.947	.976	.555	.323	.952	.536	
	N	100	100	100	100	100	100	100	100	
rsteplength	Pearson Correlation	-.138	-.108	.260**	.178	-.260**	-.301**	.154	-.054	
	Sig. (2-tailed)	.171	.283	.009	.077	.009	.002	.126	.590	
	N	100	100	100	100	100	100	100	100	
lstridelength	Pearson Correlation	-.068	-.091	.267**	.192	-.220*	-.238*	.173	.073	
	Sig. (2-tailed)	.499	.369	.007	.055	.028	.017	.086	.470	
	N	100	100	100	100	100	100	100	100	
rstridelength	Pearson Correlation	-.040	-.086	.290**	.215*	-.222*	-.216*	.206*	.102	
	Sig. (2-tailed)	.694	.394	.003	.032	.026	.031	.040	.311	
	N	100	100	100	100	100	100	100	100	
lsupportbase	Pearson Correlation	-.109	-.131	-.045	-.510**	.059	-.119	-.474**	-.194	
	Sig. (2-tailed)	.282	.195	.655	.000	.557	.237	.000	.053	
	N	100	100	100	100	100	100	100	100	
rsupportbase	Pearson Correlation	-.140	-.140	-.077	-.541**	.078	-.134	-.508**	-.159	
	Sig. (2-tailed)	.163	.165	.448	.000	.442	.183	.000	.114	
	N	100	100	100	100	100	100	100	100	
lsteptime	Pearson Correlation	1	.709**	-.086	.691**	.748**	.934**	.654**	.681**	
	Sig. (2-tailed)		.000	.397	.000	.000	.000	.000	.000	
	N	100	100	100	100	100	100	100	100	
rsteptime	Pearson Correlation	.709**	1	-.082	.642**	.785**	.725**	.613**	.455**	
	Sig. (2-tailed)	.000		.419	.000	.000	.000	.000	.000	
	N	100	100	100	100	100	100	100	100	
lswingtime	Pearson Correlation	-.086	-.082	1	-.052	-.089	-.106	-.053	-.064	
	Sig. (2-tailed)	.397	.419		.606	.380	.293	.600	.529	
	N	100	100	100	100	100	100	100	100	
rswingtime	Pearson Correlation	.691**	.642**	-.052	1	.512**	.643**	.946**	.613**	
	Sig. (2-tailed)	.000	.000	.606		.000	.000	.000	.000	
	N	100	100	100	100	100	100	100	100	
lstancetime	Pearson Correlation	.748**	.785**	-.089	.512**	1	.787**	.467**	.421**	
	Sig. (2-tailed)	.000	.000	.380	.000		.000	.000	.000	
	N	100	100	100	100	100	100	100	100	
rstancetime	Pearson Correlation	.934**	.725**	-.106	.643**	.787**	1	.615**	.688**	
	Sig. (2-tailed)	.000	.000	.293	.000	.000		.000	.000	
	N	100	100	100	100	100	100	100	100	
lsstime	Pearson Correlation	.654**	.613**	-.053	.946**	.467**	.615**	1	.666**	
	Sig. (2-tailed)	.000	.000	.600	.000	.000	.000		.000	
	N	100	100	100	100	100	100	100	100	
rsstime	Pearson Correlation	.681**	.455**	-.064	.613**	.421**	.688**	.666**	1	
	Sig. (2-tailed)	.000	.000	.529	.000	.000	.000	.000		
	N	100	100	100	100	100	100	100	100	
ldstime	Pearson Correlation	.641**	.547**	-.102	.195	.642**	.777**	.227*	.340**	
	Sig. (2-tailed)	.000	.000	.313	.051	.000	.000	.023	.001	
	N	100	100	100	100	100	100	100	100	
rdstime	Pearson Correlation	.739**	.546**	-.129	.328**	.670**	.802**	.357**	.394**	
	Sig. (2-tailed)	.000	.000	.201	.001	.000	.000	.000	.000	
	N	100	100	100	100	100	100	100	100	

Correlations	
	ldstime	rdstime	
meangaitspeed	Pearson Correlation	-.688**	-.667**	
	Sig. (2-tailed)	.000	.000	
	N	100	100	
lsteplength	Pearson Correlation	-.086	-.099	
	Sig. (2-tailed)	.394	.327	
	N	100	100	
rsteplength	Pearson Correlation	-.439**	-.388**	
	Sig. (2-tailed)	.000	.000	
	N	100	100	
lstridelength	Pearson Correlation	-.435**	-.346**	
	Sig. (2-tailed)	.000	.000	
	N	100	100	
rstridelength	Pearson Correlation	-.429**	-.343**	
	Sig. (2-tailed)	.000	.000	
	N	100	100	
lsupportbase	Pearson Correlation	.130	.108	
	Sig. (2-tailed)	.198	.284	
	N	100	100	
rsupportbase	Pearson Correlation	.109	.056	
	Sig. (2-tailed)	.282	.583	
	N	100	100	
lsteptime	Pearson Correlation	.641**	.739**	
	Sig. (2-tailed)	.000	.000	
	N	100	100	
rsteptime	Pearson Correlation	.547**	.546**	
	Sig. (2-tailed)	.000	.000	
	N	100	100	
lswingtime	Pearson Correlation	-.102	-.129	
	Sig. (2-tailed)	.313	.201	
	N	100	100	
rswingtime	Pearson Correlation	.195	.328**	
	Sig. (2-tailed)	.051	.001	
	N	100	100	
lstancetime	Pearson Correlation	.642**	.670**	
	Sig. (2-tailed)	.000	.000	
	N	100	100	
rstancetime	Pearson Correlation	.777**	.802**	
	Sig. (2-tailed)	.000	.000	
	N	100	100	
lsstime	Pearson Correlation	.227*	.357**	
	Sig. (2-tailed)	.023	.000	
	N	100	100	
rsstime	Pearson Correlation	.340**	.394**	
	Sig. (2-tailed)	.001	.000	
	N	100	100	
ldstime	Pearson Correlation	1	.859**	
	Sig. (2-tailed)		.000	
	N	100	100	
rdstime	Pearson Correlation	.859**	1	
	Sig. (2-tailed)	.000		
	N	100	100	

**. Correlation is significant at the 0.01 level (2-tailed).	
*. Correlation is significant at the 0.05 level (2-tailed).	
