# Supplementary material for: Feasibility and benefits of group-based exercise in residential aged care adults: a pilot study for the GrACE programme
Source: PeerJ. 2016 May 18;4:e2018. doi: 10.7717/peerj.2018 (PMC4878364; doi:10.7717/peerj.2018)
Supplement: Supplemental Information 6 [file peerj-04-2018-s006.doc]

EXAMINE VARIABLES=meangaitspeed steplength stridelength supportbase steptime swingtime stancetime singlesupportbase doublesupportbase
  /PLOT BOXPLOT STEMLEAF
  /COMPARE GROUPS
  /PERCENTILES(5,10,25,50,75,90,95) HAVERAGE
  /STATISTICS DESCRIPTIVES
  /CINTERVAL 95
  /MISSING LISTWISE
  /NOTOTAL.


Explore


Notes	
Output Created	29-DEC-2015 15:16:07	
Comments		
Input	Data	C:\Users\13133041\Desktop\gait study data spss\descriptivestats.sav	
	Active Dataset	DataSet2	
	Filter	<none>	
	Weight	<none>	
	Split File	<none>	
	N of Rows in Working Data File	100	
Missing Value Handling	Definition of Missing	User-defined missing values for dependent variables are treated as missing.	
	Cases Used	Statistics are based on cases with no missing values for any dependent variable or factor used.	
Syntax	EXAMINE VARIABLES=meangaitspeed steplength stridelength supportbase steptime swingtime stancetime singlesupportbase doublesupportbase
  /PLOT BOXPLOT STEMLEAF
  /COMPARE GROUPS
  /PERCENTILES(5,10,25,50,75,90,95) HAVERAGE
  /STATISTICS DESCRIPTIVES
  /CINTERVAL 95
  /MISSING LISTWISE
  /NOTOTAL.	
Resources	Processor Time	00:00:00.99	
	Elapsed Time	00:00:00.97	


Case Processing Summary	
	Cases	
	Valid	Missing	Total	
	N	Percent	N	Percent	N	Percent	
meangaitspeed	100	100.0%	0	0.0%	100	100.0%	
steplength	100	100.0%	0	0.0%	100	100.0%	
stridelength	100	100.0%	0	0.0%	100	100.0%	
supportbase	100	100.0%	0	0.0%	100	100.0%	
steptime	100	100.0%	0	0.0%	100	100.0%	
swingtime	100	100.0%	0	0.0%	100	100.0%	
stancetime	100	100.0%	0	0.0%	100	100.0%	
singlesupportbase	100	100.0%	0	0.0%	100	100.0%	
doublesupportbase	100	100.0%	0	0.0%	100	100.0%	


Descriptives	
	Statistic	Std. Error	
meangaitspeed	Mean	.6303	.01887	
	95% Confidence Interval for Mean	Lower Bound	.5929		
		Upper Bound	.6677		
	5% Trimmed Mean	.6256		
	Median	.6200		
	Variance	.036		
	Std. Deviation	.18869		
	Minimum	.29		
	Maximum	1.18		
	Range	.89		
	Interquartile Range	.27		
	Skewness	.432	.241	
	Kurtosis	-.077	.478	
steplength	Mean	.4123	.00773	
	95% Confidence Interval for Mean	Lower Bound	.3970		
		Upper Bound	.4276		
	5% Trimmed Mean	.4109		
	Median	.4100		
	Variance	.006		
	Std. Deviation	.07731		
	Minimum	.28		
	Maximum	.62		
	Range	.34		
	Interquartile Range	.12		
	Skewness	.077	.241	
	Kurtosis	-.612	.478	
stridelength	Mean	.8291	.01521	
	95% Confidence Interval for Mean	Lower Bound	.7989		
		Upper Bound	.8593		
	5% Trimmed Mean	.8260		
	Median	.8300		
	Variance	.023		
	Std. Deviation	.15214		
	Minimum	.57		
	Maximum	1.25		
	Range	.68		
	Interquartile Range	.24		
	Skewness	.185	.241	
	Kurtosis	-.581	.478	
supportbase	Mean	.1551	.00584	
	95% Confidence Interval for Mean	Lower Bound	.1435		
		Upper Bound	.1667		
	5% Trimmed Mean	.1542		
	Median	.1600		
	Variance	.003		
	Std. Deviation	.05842		
	Minimum	.02		
	Maximum	.31		
	Range	.29		
	Interquartile Range	.08		
	Skewness	.085	.241	
	Kurtosis	.126	.478	
steptime	Mean	.6573	.01238	
	95% Confidence Interval for Mean	Lower Bound	.6327		
		Upper Bound	.6819		
	5% Trimmed Mean	.6487		
	Median	.6350		
	Variance	.015		
	Std. Deviation	.12376		
	Minimum	.46		
	Maximum	.99		
	Range	.53		
	Interquartile Range	.17		
	Skewness	.942	.241	
	Kurtosis	.462	.478	
swingtime	Mean	.4180	.00729	
	95% Confidence Interval for Mean	Lower Bound	.4035		
		Upper Bound	.4325		
	5% Trimmed Mean	.4120		
	Median	.4100		
	Variance	.005		
	Std. Deviation	.07295		
	Minimum	.31		
	Maximum	.64		
	Range	.33		
	Interquartile Range	.08		
	Skewness	1.376	.241	
	Kurtosis	1.929	.478	
stancetime	Mean	.9098	.01968	
	95% Confidence Interval for Mean	Lower Bound	.8707		
		Upper Bound	.9489		
	5% Trimmed Mean	.8971		
	Median	.8500		
	Variance	.039		
	Std. Deviation	.19682		
	Minimum	.59		
	Maximum	1.45		
	Range	.86		
	Interquartile Range	.26		
	Skewness	.947	.241	
	Kurtosis	.307	.478	
singlesupportbase	Mean	.4168	.00705	
	95% Confidence Interval for Mean	Lower Bound	.4028		
		Upper Bound	.4308		
	5% Trimmed Mean	.4109		
	Median	.4100		
	Variance	.005		
	Std. Deviation	.07045		
	Minimum	.31		
	Maximum	.64		
	Range	.33		
	Interquartile Range	.08		
	Skewness	1.342	.241	
	Kurtosis	2.072	.478	
doublesupportbase	Mean	.2398	.00701	
	95% Confidence Interval for Mean	Lower Bound	.2259		
		Upper Bound	.2537		
	5% Trimmed Mean	.2361		
	Median	.2200		
	Variance	.005		
	Std. Deviation	.07014		
	Minimum	.12		
	Maximum	.44		
	Range	.32		
	Interquartile Range	.09		
	Skewness	.817	.241	
	Kurtosis	.264	.478	


Percentiles	
	Percentiles	
	5	10	25	50	
Weighted Average(Definition 1)	meangaitspeed	.3210	.4010	.4800	.6200	
	steplength	.2900	.2910	.3500	.4100	
	stridelength	.5800	.6210	.7000	.8300	
	supportbase	.0600	.0710	.1200	.1600	
	steptime	.5105	.5300	.5525	.6350	
	swingtime	.3305	.3400	.3700	.4100	
	stancetime	.6805	.7110	.7600	.8500	
	singlesupportbase	.3305	.3400	.3700	.4100	
	doublesupportbase	.1500	.1600	.1900	.2200	
Tukey's Hinges	meangaitspeed			.4800	.6200	
	steplength			.3500	.4100	
	stridelength			.7000	.8300	
	supportbase			.1200	.1600	
	steptime			.5550	.6350	
	swingtime			.3700	.4100	
	stancetime			.7600	.8500	
	singlesupportbase			.3700	.4100	
	doublesupportbase			.1900	.2200	

Percentiles	
	Percentiles	
	75	90	95	
Weighted Average(Definition 1)	meangaitspeed	.7500	.9100	.9600	
	steplength	.4675	.5190	.5390	
	stridelength	.9400	1.0380	1.0785	
	supportbase	.2000	.2100	.2700	
	steptime	.7200	.8360	.9580	
	swingtime	.4500	.4800	.6100	
	stancetime	1.0200	1.2350	1.3000	
	singlesupportbase	.4500	.4800	.6095	
	doublesupportbase	.2800	.3570	.3695	
Tukey's Hinges	meangaitspeed	.7500			
	steplength	.4650			
	stridelength	.9400			
	supportbase	.2000			
	steptime	.7200			
	swingtime	.4500			
	stancetime	1.0200			
	singlesupportbase	.4500			
	doublesupportbase	.2800			


meangaitspeed


meangaitspeed Stem-and-Leaf Plot

 Frequency    Stem &  Leaf

     4.00        2 .  9999
     3.00        3 .  244
      .00        3 .
     9.00        4 .  000113444
    10.00        4 .  5677777788
     8.00        5 .  11224444
    12.00        5 .  555556668888
    11.00        6 .  11222233444
    11.00        6 .  55566778999
     6.00        7 .  000234
     5.00        7 .  55668
     7.00        8 .  0112234
     3.00        8 .  569
     5.00        9 .  11122
     2.00        9 .  66
     2.00       10 .  04
     1.00       10 .  7
     1.00 Extremes    (>=1.18)

 Stem width:       .10
 Each leaf:       1 case(s)


öÙPÖyüxøða¿QVeen	ÿkíÙþ/¿Ü$hðeUVæÿÞÖ4øý>óWeEYe5Ê²*+ÊjUYAYAY²¢¬Ê²eeeUVPVPV£¬(«²¢¬FYQVeEY²²²*+(+(«QVUYQV£¬ ¬ ¬ÊÊÊjeUVÕ(+Êª¬(«²*+(+(«²*+Êª¬(«QVUYQV£¬ ¬ ¬ÊÊÊjeUVÕ(+(+(«²²²eEYe5Ê²*+ÊjUYAYAY²¢¬Ê²eeeUVPVPV£¬(«²¢¬FYQVeEYUYAYAY²¢¬Ê²eEYe5ÊÊÊª¬ ¬ ¬FYQVeEY²²²*+(+(«QVUYQV£¬(«²¢¬FYAYAYÕ(+Êª¬(«QVPVPVeee5Ê²*+ÊjeUVeee5Ê²*+ÊjeUV²Öð¥¬(«²¢¬(+Êª¬¸lÜeeUVPVPV£¬(«²¢¬FYAYAYæDYÅb6M¥RMMMýýýå»nß¾½uëÖt:½lÙ²eEY²¢¬Ó5ã¾¾¾°èîîîèè(ßµsçÎÝ»wOLL¬._¾|ttô|eEY²¢¬¿ÉdB;Ã¢T*år¹ò]á*vpppòÃ®®®ÊyV[[«¬(«QVõT*Uu|øÑGÕÕÕÖsçÎýüóÏÿSÁ5+Êjeý(&×étzÊ®ýû÷Å¥KZZZü=+Êje¾¬õõõ¥R)¹ÖSvÝïrVYQV£¬(kõ²vvvöööExã¸|×öíÛ?ÿüó°¸páÂºuëe5Ê²N_Ö|>ßÐÐEQ6-¿Zs÷à7n´··«Õ¡¡!eEY²¢¬~RÊjUYAYAY²¢¬Ê²eeeUVPVPV£¬(«²¢¬FYAYAYÕ(+Êª¬(«QVUYQV£¬ ¬ ¬ÊÊÊjeUVÕ(+(+(«²²²eEYe5Ê²*+ÊjUYAYAY²¢¬Ê²eeeUVPVPV£¬(«²¢¬FYAYAYÕ(+Êª¬(«QVUYQV£¬ ¬ ¬ÊÊÊjeUVÕ(+(+(«²²²eEYe5Ê²*+ÊjUYýFYe5Ê²*+ÊjUYAYAY²¢¬Ê²eeeUVPVPV£¬(«²¢¬FYQVeå±*ëSìûïÿ4¹ù÷ûWeEY9çÔ©SKæ^|ñïórþ¥áéEþ6_ÿë.¦§§ÇïaUYaö¬_¿¾¦¦ÆëÊ(+(«²²ÊÊ(+ ¬ ¬ÊÊÊª¬ ¬²ÊÊ(+(«²²ÊÊ(+ ¬ ¬ÊÊÊª¬ ¬²ÊÊ(+(«²²ÊÊª¬ ¬²²*+(+ ¬ ¬²ÊÊª¬ ¬ ¬ÊÊ(+ ¬ ¬²²*+(+ ¬ ¬²ÊÊª¬ ¬ ¬ÊÊÌvYÅb6M¥RMMMýýý9r$¼õÊÇq___XtwwwttLÙçÎ5kÖ$e=~üø?*DQ¤¬ ¬ ¬÷d2°(J¹ÊÞ®®®=ö$eýôÓO[+ÔÖÖ*+(+(ë=©Tªê:¸zõjKKKè®»Á ¬ ¬ZÖ(&×étº|Wû'î©¬ ¬ ¬XÖúúúR©Üë_ðkÊÊÊ:Y;;;Ã"<Æq×¬ ¬ ¬ZÖ|>ßÐÐEQ6-USª¬ ¬ ¬~R(+ ¬²²*+(+(«²²Ê(+(+ ¬ ¬ÊÊ(+(+ ¬²²*+(+(«²²Ê(+(+ ¬ ¬ÊÊ(+(+ ¬²²*+(+(«²²Ê(+(«²²²*+(+ ¬²²ÊÊª¬ ¬²²Ê(+(«²²²*+(+ ¬²²ÊÊª¬ ¬²²Ê(+(«²²²*+(+ ¬²²*+(+(«²²Ê(+(+ ¬ ¬Ê¿_Í¼vêÔ)¿Ä(«²Â¬êééÙ1]»vÍ/1Êª¬ ¬ ¬Ê²*+(+(+(«²²²²*+Êª¬ ¬ ¬ ¬ÊÊÊÊª¬(«²²²*+Êª¬ ¬ ¬ ¬ÊÊÊÊª¬(«²²²²*+(+(+(«²Àï,k±XÌf³©Tª©©©¿¿¿|W¡PX³fMØÕÜÜSVuú²ÆqÜ××ÝÝÝå»V¬X<ÑV®¬(ëôeÍd2aQ*r¹ÜýîúÖÕÕô.­P[[«¬(ë=©TªêºÜ3g¶mÛvöìÙ*DQ¤¬(ë=!ët:]yÀÍ7ã8¾uë»Á(ëôe­¯¯/JÉÝà°²wxxxëÖ­###¾7e ²vvvöööEx×¦å»6nÜ866æ_Ý ¬ZÖ|>ßÐÐEQ6-¿Zs÷àWSFYPV?)UY@Y@Y@YUYPVeeeeUVPVPVPVe@Yg§¬_ýõ·0­^½zöÊúüóÏ/fÑÂ,XàuÙôÌ3Ï?~ÊÌ²wßwíÚµ^PV@YAYeePVPV@Y188øÍ7ßx@Y@YeÇå-]s÷MÝÞÞþg@Yá±ë«²²Â<,ÜÒéôÞ½?þøã°8zôhØ>22²aÃT*ÕÚÚúã?-Åb±¥¥%l©¯¯§$çîÞ½Ù²eacrÖððp[[[&	Oä­êYá³Ôü¿ðá#GÂÞ(óù|rÀÉ'/^N¼~ýzÕ/¦rK82|êEõÕWÊÊ²¬ºråJHTdÈUØ¾eËðaX8q"4,,Ã:,._¾²ûáNLLLÇqhmXìÜ¹3É[Õ³Ê/+Ce»ººÂ¢¿¿ùòåÉ®pzxÚðT¯¾újÕ/¦rKò©Ã³)+(+<Ê²§.YL^VI5ÇÇÇCº:;;C,'ë[~V²åÎ;Éªgºýû÷ËÙ·ÞzëÒ¥KOU*'©«««úÅTÝ2åSÊ¦¬U!T·oß.?²­­íàÁ×¯_RÇß(ëýÎ*?7úôööö»¼¬S¾ª[Âñaª¬¬ ¬0çÊúÒK/%7iC×­[¤ëòåË·nÝÚ¶mÛýÊÚÑÑ9::ºaÃÉÖV=+¢$!¨ÉÝ¾¾¾L&°eË±±±]»vÅq©Ü|ï½÷®²lÙ2ee9WÖáááP¬ÐÅæææ¡¡¡°eß¾O>ùäâÅ÷îÝ¿²ÔbÎý³6mÚP,BhS©TØp@èeØ	®úÅTn	zýúõ+W®¼pá²²Â|ÒØÒÒò±wÛ·o_´hQ¸lmm×3äZPVPVPV@Y@Y@YeÙó¿ÒÑßÁIEND®B`


steplength


steplength Stem-and-Leaf Plot

 Frequency    Stem &  Leaf

    10.00        2 .  8889999999
    11.00        3 .  00112222444
    20.00        3 .  55555667777788888899
    22.00        4 .  0000111111222233334444
    23.00        4 .  56666666666677778889999
    10.00        5 .  0011222224
     3.00        5 .  557
     1.00        6 .  2

 Stem width:       .10
 Each leaf:       1 case(s)


a~g³Ye@YWÒ%7óù|]]]"¢httTYPVW@YUYPVeeeUVUYPVeeUVUYPVeeeUVUY@Y@YeUVUY@Y@YeUVPVPVe@YeUVPVe@YeUVPVPVe@YUYPVe@YUYPVe@YUYPVe@YUYPVe@YUYPVeeeUVUYPVeeUVUYPVeeeUVUYPVeeUVUYPVeeeUVUY@Y@YeUVUY@YeUVUY@Y@YeUVUY@YeUVUY@Y@YeUVPV¿ufcGY_8*Yl§N²Ã+«²¢¬ ¬(+ÊÊ²*«²²¢¬Ê²²*«²¢¬ ¬(«²*+(+ÊúËÆÇÇÛÚÚÉdKKK>KÆÆÆ¢((«²²¢¬«òyùòå0d³Ùþþþ0èééÉårað×¿þu`±³gÏ¦R)UYAYQÖºråJkkk¾Å0(L&^õÕ'YZUYAYQÖÌÏÏWWW]-LFÃÍÌÒ]ÇÎ++(+ÊºÃÃÃét:Dia*«²²¢¬eg¨¡¯B!>·VYeTãããa0::zèÐ¡0èèèèëëð;Í*«²²¢¬$ì[ñglZZZÂlõàÁaI>¯««K$QÜ*«²²¢¬®²²*«²¢¬ ¬(+ÊÊ²*+Ê²¢¬Ê²²¢¬(+(+Êª¬ÊÊ²*+ÊÊª¬Ê²²¢¬Êª¬ ¬(«²¢¬ ¬Êª¬(+(+Ê²²¢¬Êª¬Ê²¢¬Ê²²¢¬(+(+Êª¬ÊÊ²*+ÊÊª¬Ê²²¢¬(+(+Êª¬(+Êj=(«²¢¬ ¬(+ÊÊ²*«²²¢¬Ê²²*«²¢¬ ¬(«²*+(+Êª¬(+(«²*+ÊÊ²¢¬ ¬(«²¢¬(+Êª¬(+(+Ê²²¢¬Êª¬ ¬(«²¢¬ ¬Êª¬(+(+Êª¬ÊÊ²*+Ê²ÚáUYQVPVeeEYUYAYQVeEYAYUYQVPVUYeUVUYeeEYQVPVUYQVeUVu+ëøøxSSSUUU"7÷íÛ§¬(+(«²ixx¸âïâ²Á#GeeUÖrÔÕÕýãË/¿Ö0UVUYËãA(k±X,%VYQVPVe]µ]»výctt4j<H§ÓÊ²²*k9*TVUYË±¾¾>J%É(¼7eeUVgEYAYQVeUVPV¶XYB<nllûJ&QVUYË²Æ¦³³³ô¦¦¦&eEYAYµAE»ví»K2TVUYË,kØ?fggÃïø´°²¢¬ ¬ÊZ~Y3Lé$pWWW¶¶6eEYAYµÓÓÓét:Ì¹dÏ=ÞÁ²²*«OÝ ¬ ¬lÈ²ÎÎÎ<yRYQVPVe-ÇÄÄDmmmü>¦%_Ôª¬(+(«²ùý¬¥R©û÷ï++ÊÊª¬åê&D.¢ðll,Ü<pà²¢¬ ¬ÊZ~YÃàØ±cï¾ûîÔÔ³Á(+(«²_Ö¸£wîÜ)öÍç(+(«²i~~>¢xÜÝÝJ¥jjjnß¾­¬(+(«²ú<+ÊÊ²¢¬ ¬l±²ÖÕÕ%d2ÉdeeUÖ2>ºbeEYAYµ©T*ìï¼óÎÌÌL|]Ãp³²²RYQVPVe-¿¬«¢¢ººZYQVPVe-GoooØ?Þ~ûí0a-ñÉá.(+ÊÊª¬«PñP®Á²²*ëª/jøÉdRYQVPVeõyVßº¬KÎý~ÿý÷MMMÊ²²*ëu~~>,q6eeUÖr¾åæAï`ªªªRVUYWçÎ;ñ³ÆÓÖÊÊÊ3gÎ(+ÊÊª¬e~óùã9÷«¬ÊÊÊ¶(k&ill|<_Èª¬ÊÊÊ¶³.¹º¡²¢¬ ¬ÊZ~ûúúâ«ÎÌÌEeEYAYõ×~êæ×_ÝptttïÞ½Éd²µµull,,	¿£(KZZZFFFUYAYÙ.e]«666ÆÖÀÀ@SSSd³Ùþþþ0èééÉårÊª¬ ¬l²®¹ø»]kkkãsËB!ÉÁ'ªê©§BÂmÔÇ¦¾~wû³ëôS]ýdøY§'okû§?ý©ÝdU^y¥ãl^§òhX¿>üìÞ°õG7nÜ8~üx,õÆãðwÿçbôÑÎ;mÔÇ9­ìÿ·7ãÏû]ÿJÙUXÕÿöÒ&ÝçÃÿ[¿øâqutt´¾¾>ù[·n÷<333Ùlvvv6>É¼ðËÕÞ eýò¿ÏoÆÿz÷îÈdËgC¢6é>ùýïuupppù;Wû<÷îÝ;zôèääd|3Nølp+«²*+Êª¬Û¥¬µµµñ§nÅâüü|jávíÐ¡C¡¥%ñ§zÂDVYUYQVeÝ.e'©ùö_üçñNyÃ|>_WW'¢ÑÑQeUVeEYuÍYO81ÿ07C])BYeUVe-Ç§~ºüuÖ¡¡!eUVeEYUYËtóæÍT*UYY:y«¬Êª¬(«²*ë¦¬Êª¬(«²*«²*«²¢¬Êª¬¿äâÅQÅÎd2×®]SVeUVUYµL|ðÁòw0]¸pAYUYQVeUÖr¤R©pÀíììéêê7ã«ê+«²*+Êª¬ÊZfY¡ªªJYUYQVeUÖrôööî[o½&¬!uÇ7UYeUVe-ÿêòè9TVeUVUYõoñ×½=ÄÂ¯YUVeUVUYÕçYQVUYQVUYQVeÝÔeu¥eUVUYÕ"PVPV6dY])BYeUVeu¥ZVWPVeEYUY])eee£Õ"UYQVeUVgEYAYQVeUVeEYu;õñ¿¼ª¬Êª¬(«²nå²^R]XÖuyUYUYQVeÝ.gçææN<¿/_VVeUVUYuÅpüM§ÓÊª¬Ê²*«²®AVóù¼×YUYQVeUÖ5~S½²*«²¢¬Êª¬ks¥ÕB¡ ¬Êª¬(«²*«Ï³¢¬ ¬(+Êª¬(«²n¥²Ò«ªáàÉdUYeUVe-SÈjüNàÎÎÎÒ;uKuózòÉJ[ees5~×RDQ´k×®¹¹9×`RÖ(JÙ(+¦¬á°5;;[ú°²ná²Ífüùàßÿ5êlAÍQÖL&StuuÅemkkSÖ-YV¯³¢¬ÊÊºuzz:Nk.ìÙ³Ç;UYQVeUVºAYAYQVeUVeEYUYUYeUVeUVeUVeUVÊµÿ?ÿåÏ¹MúI³ºßý²*«²*«²²±ìÞýûMueUVeUVeecyúéTVeUVÖðpéuVeUVUYUYUYQVeUVeUVeEYeEYAYQVeUVeEYUYUYeUVeUVeUVeUVUYUYUYQVeUVeEYQVeEYQVeEYQVeUVeEYUYUYeUVeUVeUVeUVUYUYUYQVeUVeEYAYQVUYQVeUVeUVeEYUYUYeUVeUVeUVeUVUYUYUYQVeUVeEYAYQVeUVeEYUYUYeUVeUVeUVõ)ë¦f²*õõ»7õ¯¬Êª¬²-~l#§N²Ã+«²¢¬ ¬(+ÊÊ²*«²²¢¬Ê²²*«²¢¬ ¬(«²*+(+Êª¬(+(«²*+ÊÊ²¢¬ ¬(«²¢¬(+Êª¬(+(+Ê²²¢¬Êª¬ ¬(«²¢¬ ¬Êª¬(+(+Êª¬ÊÊ²>8L¦tsll,¢d2ÙÒÒ222¢¬ÊÊ²®B>onnûYiI6íïï|òÉ©ÅÞ|óÍ;wÚ¨ÊÊ².uøðáÛ·o/,kmmm±XB¡Ïeßï½µ¶¶I­ª¬ ¬(ëÊ5$sÅ±³ÁÊÊ²SÖD"Q§R)eUVPVõW5Nølp+«²²¢¬¿ª¬a~g³YeUVPVuÕ5]8ÎçóuuuD"¢ÑÑQeUVPVÕ"PVPVeUVeEYAYQVeEYQVUYQVPVeeEYUYAYQVeEYAYUYQVPVUYeUVUYeeEYQVPVUYUYQVUYQVPVeeEYUYAYQVeEYAYUYQVPVeeEYeEY­eUVeEYAYQVeUVPVUYQVPVeUVeUVeeEYeeUVeEYAYQVeUVeEYeeEYQVPVUYeUVUYeeEYUYAYQVeEYQV;¼²*+ÊÊ²¢¬ ¬(«²*+(+Êª¬(+(«²*+ÊÊ²*«²²¢¬Ê²²*«²¢¬ ¬(+ÊÊ²*+Ê²¢¬Ê²²¢¬(+(+Êª¬ÊÊ²*+ÊÊª¬ÊÊZ;wîÜ©GõÑÿñÇ[Ã(+Êª¬ÛqºNjjj¬aeUVeUVePV@YQVeeUVeUVePVUYeEY@YUYePVUYeEY@YQVeEYPVUYUY@YQVeeUVeUVex$¯¼òJÅz²UYíåÜ¹sÏ­l6k+«² ¬ÊÊj£ ¬Ê²*+(+(«² ¬Ê²*+(«² ¬Ê²*+(+(«² ¬ÊÊÛ£¬cccQ%É(+Êú¨²Ùlôôôär¹LOOß[l||<JÙ¨(ëRµµµÅb1B&YqÉÑ£G+Ù±c².L&/ùæon,öÙg³ ¬+H$¥qåK¼Î²>ªt:](âs¿a¼âe@YUGGG___ßÙlvÅ%Ê²þ²ÿ§óù|]]]"¢htttÅ%Ê²ºRÊª¬ ¬ ¬Ê²*+(+(«² ¬Ê²*+(«² ¬Ê²*+(+(«² ¬Ê²n©²¦R©ÿßÎ³Ï>»uÊ:55æ¬)¶?±Ø&Éd"°¶ÊÊÊ7nl²²¼øâGµØ&N>ýôÓO[[²¢¬ ¬(+ÊÊ²²²*+(+(+Ê²²¢¬l[.]¶Ø&ÆÆÆÎ?o=(+ ¬ ¬lÃÃÃ©Tª¯¯ïWíöO¶ôñ×®¬ðèÉäÕ«WwÀ®¬°6ÇXONN<x0¶­­íë¯¿ï3ÚÞÞÞ³gÏAÜà±±±ûöG¦ÓéðÒqgù3ÀßÿíáÊëøã/¿ürüÖßë×¯ÃG|×¥K¾ýöÛp	wN8¦åÍÍÍá1a011QSSSzåÏ|ç·++¬cYÃ°¦°ññ%ÅâÂÇÄ©©©÷ß¿££#â%ñïåÏ|ç·++¬oYçææV¼kÉ`ÿþý×®]»÷îãÎgM±óÛÃÖåàòÂ/twwA8¦8pà!Çp|=~üøÂãÎògÍXV¸²ÂÚÝ»áÒÚÚzûöíwÎ?_UUUSSÓÛÛ»ð¸³ü`3Õ®¬²²²ÊÊÊÊ(+(+(+ ¬ ¬ ¬²²²²ÝW_õÜsÏ%ÉÊÊÊçþîÝ»¥».^¼ØØØîjmm½råJéñD"ÉÅB¡EQ¸Z ¬ÀZZZ*Ø¿¼|xx¸b±7nÄwuvvýýýgÎ	îîn«øYq:.ªÆ<_½z5óù|çr¹ø®¹¹¹æææ0aaW«øÙ±cÇB5C_ÛÛÛ»ºº¦§§ãå©TjÉµºººô¯®/¼yó¦uÊü¿ùùùÔ^z)L@C):/O&KÊê[úW~øa¼ðüùóÖ!(+°©©©PÊÔøf[[[¸9<<¼â#wíÚ¼wïÞ07­=PVàgX81moo.³>|8¾ëµ×^7ÂàÄV#(+ð³ÉÉÉl6[YYf«¡÷îÝ+ÝÕßßßÜÜH$jjjÞxãø%Ø[·nÖ××q±XÌd2áfXhM²²²ÊÊÊÊ(+(+(+ ¬ ¬ ¬²²²²«÷¿ñ¨X IEND®B`


steptime


Stem-and-Leaf Plots


steptime Stem-and-Leaf Plot for
sex= female

 Frequency    Stem &  Leaf

     2.00        4 .  69
    27.00        5 .  001233333333344445556667888
    16.00        6 .  0000113334444557
    17.00        7 .  00111122222688889
     1.00        8 .  0
     2.00        9 .  16
     2.00 Extremes    (>=.98)

 Stem width:       .10
 Each leaf:       1 case(s)


steptime Stem-and-Leaf Plot for
sex= male

 Frequency    Stem &  Leaf

     2.00        5 .  23
     5.00        5 .  56799
     6.00        6 .  011224
     4.00        6 .  5677
     6.00        7 .  000001
     4.00        7 .  6889
     2.00        8 .  44
     1.00        8 .  5
     1.00        9 .  2
     2.00        9 .  88

 Stem width:       .10
 Each leaf:       1 case(s)
