# Supplementary material for: Feasibility and benefits of group-based exercise in residential aged care adults: a pilot study for the GrACE programme
Source: PeerJ. 2016 May 18;4:e2018. doi: 10.7717/peerj.2018 (PMC4878364; doi:10.7717/peerj.2018)
Supplement: Supplemental Information 7 [file peerj-04-2018-s007.doc]

NEW FILE.
DATASET NAME DataSet1 WINDOW=FRONT.
GET
  FILE='C:\Users\13133041\Desktop\gait study data spss\everything is in this file.sav'.
DATASET NAME DataSet2 WINDOW=FRONT.
DATASET ACTIVATE DataSet1.
DATASET CLOSE DataSet2.
ONEWAY meangaitspeed steplength stridelength supportbase steptime swingtime stancetime singlesupportbase doublesupportbase BY racf
  /POLYNOMIAL=1
  /STATISTICS DESCRIPTIVES EFFECTS HOMOGENEITY BROWNFORSYTHE WELCH
  /PLOT MEANS
  /MISSING ANALYSIS
  /POSTHOC=TUKEY SCHEFFE GT2 ALPHA(0.05).


Oneway


Notes	
Output Created	21-DEC-2015 17:28:39	
Comments		
Input	Active Dataset	DataSet1	
	Filter	<none>	
	Weight	<none>	
	Split File	<none>	
	N of Rows in Working Data File	100	
Missing Value Handling	Definition of Missing	User-defined missing values are treated as missing.	
	Cases Used	Statistics for each analysis are based on cases with no missing data for any variable in the analysis.	
Syntax	ONEWAY meangaitspeed steplength stridelength supportbase steptime swingtime stancetime singlesupportbase doublesupportbase BY racf
  /POLYNOMIAL=1
  /STATISTICS DESCRIPTIVES EFFECTS HOMOGENEITY BROWNFORSYTHE WELCH
  /PLOT MEANS
  /MISSING ANALYSIS
  /POSTHOC=TUKEY SCHEFFE GT2 ALPHA(0.05).	
Resources	Processor Time	00:00:02.04	
	Elapsed Time	00:00:01.98	


Descriptives	
	N	Mean	Std. Deviation	Std. Error	95% Confidence Interval for Mean	Minimum	Maximum	
					Lower Bound	Upper Bound			
meangaitspeed	darlington	17	.6400	.16186	.03926	.5568	.7232	.44	.96	
	terraces	43	.6570	.17302	.02639	.6037	.7102	.34	1.18	
	ozcare	40	.5975	.21321	.03371	.5293	.6657	.29	1.07	
	Total	100	.6303	.18869	.01887	.5929	.6677	.29	1.18	
	Model	Fixed Effects			.18858	.01886	.5929	.6677			
		Random Effects				.01950	.5464	.7142			
steplength	darlington	17	.4324	.05215	.01265	.4055	.4592	.32	.52	
	terraces	43	.4167	.07686	.01172	.3931	.4404	.29	.62	
	ozcare	40	.3990	.08554	.01352	.3716	.4264	.28	.57	
	Total	100	.4123	.07731	.00773	.3970	.4276	.28	.62	
	Model	Fixed Effects			.07713	.00771	.3970	.4276			
		Random Effects				.00875	.3746	.4500			
stridelength	darlington	17	.8547	.11609	.02816	.7950	.9144	.64	1.04	
	terraces	43	.8386	.15256	.02327	.7917	.8856	.57	1.25	
	ozcare	40	.8080	.16530	.02614	.7551	.8609	.57	1.16	
	Total	100	.8291	.15214	.01521	.7989	.8593	.57	1.25	
	Model	Fixed Effects			.15260	.01526	.7988	.8594			
		Random Effects				.01526a	.7634a	.8948a			
supportbase	darlington	17	.1424	.05081	.01232	.1162	.1685	.06	.21	
	terraces	43	.1530	.05026	.00767	.1376	.1685	.06	.31	
	ozcare	40	.1628	.06891	.01090	.1407	.1848	.02	.31	
	Total	100	.1551	.05842	.00584	.1435	.1667	.02	.31	
	Model	Fixed Effects			.05856	.00586	.1435	.1667			
		Random Effects				.00586a	.1299a	.1803a			
steptime	darlington	17	.6924	.16449	.03989	.6078	.7769	.49	.98	
	terraces	43	.6451	.11185	.01706	.6107	.6795	.52	.96	
	ozcare	40	.6555	.11664	.01844	.6182	.6928	.46	.99	
	Total	100	.6573	.12376	.01238	.6327	.6819	.46	.99	
	Model	Fixed Effects			.12390	.01239	.6327	.6819			
		Random Effects				.01239a	.6040a	.7106a			
swingtime	darlington	17	.4424	.09490	.02302	.3936	.4911	.33	.62	
	terraces	43	.4084	.06156	.00939	.3894	.4273	.31	.62	
	ozcare	40	.4180	.07328	.01159	.3946	.4414	.31	.64	
	Total	100	.4180	.07295	.00729	.4035	.4325	.31	.64	
	Model	Fixed Effects			.07270	.00727	.4036	.4324			
		Random Effects				.00859	.3811	.4549			
stancetime	darlington	17	.9518	.25800	.06257	.8191	1.0844	.63	1.42	
	terraces	43	.8870	.18326	.02795	.8306	.9434	.68	1.42	
	ozcare	40	.9165	.18295	.02893	.8580	.9750	.59	1.45	
	Total	100	.9098	.19682	.01968	.8707	.9489	.59	1.45	
	Model	Fixed Effects			.19743	.01974	.8706	.9490			
		Random Effects				.01974a	.8249a	.9947a			
singlesupportbase	darlington	17	.4418	.09567	.02320	.3926	.4910	.32	.62	
	terraces	43	.4037	.05201	.00793	.3877	.4197	.31	.59	
	ozcare	40	.4203	.07388	.01168	.3966	.4439	.31	.64	
	Total	100	.4168	.07045	.00705	.4028	.4308	.31	.64	
	Model	Fixed Effects			.06983	.00698	.4029	.4307			
		Random Effects				.01003	.3737	.4599			
doublesupportbase	darlington	17	.2429	.07920	.01921	.2022	.2837	.12	.39	
	terraces	43	.2340	.06898	.01052	.2127	.2552	.15	.44	
	ozcare	40	.2448	.06872	.01087	.2228	.2667	.12	.44	
	Total	100	.2398	.07014	.00701	.2259	.2537	.12	.44	
	Model	Fixed Effects			.07067	.00707	.2258	.2538			
		Random Effects				.00707a	.2094a	.2702a			

Descriptives	
	Between- Component Variance	
		
meangaitspeed	darlington		
	terraces		
	ozcare		
	Total		
	Model	Fixed Effects		
		Random Effects	.00007	
steplength	darlington		
	terraces		
	ozcare		
	Total		
	Model	Fixed Effects		
		Random Effects	.00005	
stridelength	darlington		
	terraces		
	ozcare		
	Total		
	Model	Fixed Effects		
		Random Effects	-.00022	
supportbase	darlington		
	terraces		
	ozcare		
	Total		
	Model	Fixed Effects		
		Random Effects	-.00003	
steptime	darlington		
	terraces		
	ozcare		
	Total		
	Model	Fixed Effects		
		Random Effects	-.00005	
swingtime	darlington		
	terraces		
	ozcare		
	Total		
	Model	Fixed Effects		
		Random Effects	.00006	
stancetime	darlington		
	terraces		
	ozcare		
	Total		
	Model	Fixed Effects		
		Random Effects	-.00038	
singlesupportbase	darlington		
	terraces		
	ozcare		
	Total		
	Model	Fixed Effects		
		Random Effects	.00014	
doublesupportbase	darlington		
	terraces		
	ozcare		
	Total		
	Model	Fixed Effects		
		Random Effects	-.00012	

a. Warning: Between-component variance is negative. It was replaced by 0.0 in computing this random effects measure.	


Test of Homogeneity of Variances	
	Levene Statistic	df1	df2	Sig.	
meangaitspeed	1.533	2	97	.221	
steplength	4.029	2	97	.021	
stridelength	2.139	2	97	.123	
supportbase	1.383	2	97	.256	
steptime	2.828	2	97	.064	
swingtime	2.060	2	97	.133	
stancetime	2.368	2	97	.099	
singlesupportbase	3.229	2	97	.044	
doublesupportbase	.283	2	97	.754	


ANOVA	
	Sum of Squares	df	Mean Square	F	Sig.	
meangaitspeed	Between Groups	(Combined)	.075	2	.038	1.058	.351	
		Linear Term	Unweighted	.022	1	.022	.606	.438	
			Weighted	.042	1	.042	1.186	.279	
			Deviation	.033	1	.033	.929	.337	
	Within Groups	3.449	97	.036			
	Total	3.525	99				
steplength	Between Groups	(Combined)	.015	2	.007	1.241	.294	
		Linear Term	Unweighted	.013	1	.013	2.231	.139	
			Weighted	.015	1	.015	2.477	.119	
			Deviation	.000	1	.000	.004	.948	
	Within Groups	.577	97	.006			
	Total	.592	99				
stridelength	Between Groups	(Combined)	.033	2	.016	.705	.497	
		Linear Term	Unweighted	.026	1	.026	1.118	.293	
			Weighted	.032	1	.032	1.359	.247	
			Deviation	.001	1	.001	.051	.822	
	Within Groups	2.259	97	.023			
	Total	2.292	99				
supportbase	Between Groups	(Combined)	.005	2	.003	.771	.465	
		Linear Term	Unweighted	.005	1	.005	1.447	.232	
			Weighted	.005	1	.005	1.541	.217	
			Deviation	.000	1	.000	.001	.970	
	Within Groups	.333	97	.003			
	Total	.338	99				
steptime	Between Groups	(Combined)	.027	2	.014	.893	.413	
		Linear Term	Unweighted	.016	1	.016	1.056	.307	
			Weighted	.009	1	.009	.562	.455	
			Deviation	.019	1	.019	1.223	.272	
	Within Groups	1.489	97	.015			
	Total	1.516	99				
swingtime	Between Groups	(Combined)	.014	2	.007	1.331	.269	
		Linear Term	Unweighted	.007	1	.007	1.338	.250	
			Weighted	.003	1	.003	.627	.430	
			Deviation	.011	1	.011	2.034	.157	
	Within Groups	.513	97	.005			
	Total	.527	99				
stancetime	Between Groups	(Combined)	.054	2	.027	.694	.502	
		Linear Term	Unweighted	.015	1	.015	.381	.539	
			Weighted	.004	1	.004	.098	.754	
			Deviation	.050	1	.050	1.290	.259	
	Within Groups	3.781	97	.039			
	Total	3.835	99				
singlesupportbase	Between Groups	(Combined)	.018	2	.009	1.890	.157	
		Linear Term	Unweighted	.006	1	.006	1.133	.290	
			Weighted	.002	1	.002	.325	.570	
			Deviation	.017	1	.017	3.454	.066	
	Within Groups	.473	97	.005			
	Total	.491	99				
doublesupportbase	Between Groups	(Combined)	.003	2	.001	.262	.770	
		Linear Term	Unweighted	.000	1	.000	.008	.930	
			Weighted	.000	1	.000	.081	.777	
			Deviation	.002	1	.002	.443	.507	
	Within Groups	.484	97	.005			
	Total	.487	99				


Robust Tests of Equality of Means	
	Statistica	df1	df2	Sig.	
meangaitspeed	Welch	.959	2	46.661	.391	
	Brown-Forsythe	1.138	2	78.599	.326	
steplength	Welch	1.602	2	53.123	.211	
	Brown-Forsythe	1.474	2	92.645	.234	
stridelength	Welch	.773	2	50.236	.467	
	Brown-Forsythe	.804	2	88.268	.451	
supportbase	Welch	.760	2	45.508	.473	
	Brown-Forsythe	.822	2	75.980	.443	
steptime	Welch	.591	2	40.038	.559	
	Brown-Forsythe	.726	2	41.382	.490	
swingtime	Welch	.972	2	39.824	.387	
	Brown-Forsythe	1.094	2	42.720	.344	
stancetime	Welch	.556	2	40.292	.578	
	Brown-Forsythe	.573	2	42.533	.568	
singlesupportbase	Welch	1.584	2	38.299	.218	
	Brown-Forsythe	1.484	2	39.126	.239	
doublesupportbase	Welch	.267	2	42.460	.767	
	Brown-Forsythe	.243	2	55.714	.785	

a. Asymptotically F distributed.	


Post Hoc Tests


Multiple Comparisons	
Dependent Variable	(I) racf	(J) racf	Mean Difference (I-J)	Std. Error	Sig.	95% Confidence Interval	
						Lower Bound	Upper Bound	
meangaitspeed	Tukey HSD	darlington	terraces	-.01698	.05403	.947	-.1456	.1116	
			ozcare	.04250	.05460	.717	-.0875	.1725	
		terraces	darlington	.01698	.05403	.947	-.1116	.1456	
			ozcare	.05948	.04143	.327	-.0391	.1581	
		ozcare	darlington	-.04250	.05460	.717	-.1725	.0875	
			terraces	-.05948	.04143	.327	-.1581	.0391	
	Scheffe	darlington	terraces	-.01698	.05403	.952	-.1513	.1173	
			ozcare	.04250	.05460	.739	-.0932	.1782	
		terraces	darlington	.01698	.05403	.952	-.1173	.1513	
			ozcare	.05948	.04143	.361	-.0435	.1625	
		ozcare	darlington	-.04250	.05460	.739	-.1782	.0932	
			terraces	-.05948	.04143	.361	-.1625	.0435	
	Hochberg	darlington	terraces	-.01698	.05403	.985	-.1482	.1142	
			ozcare	.04250	.05460	.821	-.0901	.1751	
		terraces	darlington	.01698	.05403	.985	-.1142	.1482	
			ozcare	.05948	.04143	.393	-.0411	.1601	
		ozcare	darlington	-.04250	.05460	.821	-.1751	.0901	
			terraces	-.05948	.04143	.393	-.1601	.0411	
steplength	Tukey HSD	darlington	terraces	.01561	.02210	.760	-.0370	.0682	
			ozcare	.03335	.02233	.298	-.0198	.0865	
		terraces	darlington	-.01561	.02210	.760	-.0682	.0370	
			ozcare	.01774	.01694	.549	-.0226	.0581	
		ozcare	darlington	-.03335	.02233	.298	-.0865	.0198	
			terraces	-.01774	.01694	.549	-.0581	.0226	
	Scheffe	darlington	terraces	.01561	.02210	.780	-.0393	.0705	
			ozcare	.03335	.02233	.332	-.0222	.0889	
		terraces	darlington	-.01561	.02210	.780	-.0705	.0393	
			ozcare	.01774	.01694	.580	-.0244	.0599	
		ozcare	darlington	-.03335	.02233	.332	-.0889	.0222	
			terraces	-.01774	.01694	.580	-.0599	.0244	
	Hochberg	darlington	terraces	.01561	.02210	.859	-.0380	.0693	
			ozcare	.03335	.02233	.359	-.0209	.0876	
		terraces	darlington	-.01561	.02210	.859	-.0693	.0380	
			ozcare	.01774	.01694	.651	-.0234	.0589	
		ozcare	darlington	-.03335	.02233	.359	-.0876	.0209	
			terraces	-.01774	.01694	.651	-.0589	.0234	
stridelength	Tukey HSD	darlington	terraces	.01610	.04372	.928	-.0880	.1202	
			ozcare	.04671	.04418	.543	-.0585	.1519	
		terraces	darlington	-.01610	.04372	.928	-.1202	.0880	
			ozcare	.03060	.03352	.633	-.0492	.1104	
		ozcare	darlington	-.04671	.04418	.543	-.1519	.0585	
			terraces	-.03060	.03352	.633	-.1104	.0492	
	Scheffe	darlington	terraces	.01610	.04372	.934	-.0926	.1248	
			ozcare	.04671	.04418	.574	-.0631	.1565	
		terraces	darlington	-.01610	.04372	.934	-.1248	.0926	
			ozcare	.03060	.03352	.660	-.0527	.1139	
		ozcare	darlington	-.04671	.04418	.574	-.1565	.0631	
			terraces	-.03060	.03352	.660	-.1139	.0527	
	Hochberg	darlington	terraces	.01610	.04372	.976	-.0901	.1223	
			ozcare	.04671	.04418	.644	-.0606	.1540	
		terraces	darlington	-.01610	.04372	.976	-.1223	.0901	
			ozcare	.03060	.03352	.740	-.0508	.1120	
		ozcare	darlington	-.04671	.04418	.644	-.1540	.0606	
			terraces	-.03060	.03352	.740	-.1120	.0508	
supportbase	Tukey HSD	darlington	terraces	-.01067	.01678	.801	-.0506	.0293	
			ozcare	-.02040	.01695	.454	-.0608	.0200	
		terraces	darlington	.01067	.01678	.801	-.0293	.0506	
			ozcare	-.00973	.01286	.731	-.0403	.0209	
		ozcare	darlington	.02040	.01695	.454	-.0200	.0608	
			terraces	.00973	.01286	.731	-.0209	.0403	
	Scheffe	darlington	terraces	-.01067	.01678	.817	-.0524	.0310	
			ozcare	-.02040	.01695	.488	-.0625	.0218	
		terraces	darlington	.01067	.01678	.817	-.0310	.0524	
			ozcare	-.00973	.01286	.752	-.0417	.0223	
		ozcare	darlington	.02040	.01695	.488	-.0218	.0625	
			terraces	.00973	.01286	.752	-.0223	.0417	
	Hochberg	darlington	terraces	-.01067	.01678	.892	-.0514	.0301	
			ozcare	-.02040	.01695	.544	-.0616	.0208	
		terraces	darlington	.01067	.01678	.892	-.0301	.0514	
			ozcare	-.00973	.01286	.833	-.0410	.0215	
		ozcare	darlington	.02040	.01695	.544	-.0208	.0616	
			terraces	.00973	.01286	.833	-.0215	.0410	
steptime	Tukey HSD	darlington	terraces	.04724	.03550	.382	-.0373	.1317	
			ozcare	.03685	.03587	.561	-.0485	.1222	
		terraces	darlington	-.04724	.03550	.382	-.1317	.0373	
			ozcare	-.01038	.02722	.923	-.0752	.0544	
		ozcare	darlington	-.03685	.03587	.561	-.1222	.0485	
			terraces	.01038	.02722	.923	-.0544	.0752	
	Scheffe	darlington	terraces	.04724	.03550	.416	-.0410	.1355	
			ozcare	.03685	.03587	.592	-.0523	.1260	
		terraces	darlington	-.04724	.03550	.416	-.1355	.0410	
			ozcare	-.01038	.02722	.930	-.0780	.0573	
		ozcare	darlington	-.03685	.03587	.592	-.1260	.0523	
			terraces	.01038	.02722	.930	-.0573	.0780	
	Hochberg	darlington	terraces	.04724	.03550	.459	-.0390	.1334	
			ozcare	.03685	.03587	.664	-.0502	.1240	
		terraces	darlington	-.04724	.03550	.459	-.1334	.0390	
			ozcare	-.01038	.02722	.974	-.0765	.0557	
		ozcare	darlington	-.03685	.03587	.664	-.1240	.0502	
			terraces	.01038	.02722	.974	-.0557	.0765	
swingtime	Tukey HSD	darlington	terraces	.03398	.02083	.237	-.0156	.0836	
			ozcare	.02435	.02105	.482	-.0257	.0745	
		terraces	darlington	-.03398	.02083	.237	-.0836	.0156	
			ozcare	-.00963	.01597	.819	-.0476	.0284	
		ozcare	darlington	-.02435	.02105	.482	-.0745	.0257	
			terraces	.00963	.01597	.819	-.0284	.0476	
	Scheffe	darlington	terraces	.03398	.02083	.269	-.0178	.0858	
			ozcare	.02435	.02105	.514	-.0280	.0767	
		terraces	darlington	-.03398	.02083	.269	-.0858	.0178	
			ozcare	-.00963	.01597	.834	-.0493	.0301	
		ozcare	darlington	-.02435	.02105	.514	-.0767	.0280	
			terraces	.00963	.01597	.834	-.0301	.0493	
	Hochberg	darlington	terraces	.03398	.02083	.284	-.0166	.0846	
			ozcare	.02435	.02105	.576	-.0268	.0755	
		terraces	darlington	-.03398	.02083	.284	-.0846	.0166	
			ozcare	-.00963	.01597	.907	-.0484	.0292	
		ozcare	darlington	-.02435	.02105	.576	-.0755	.0268	
			terraces	.00963	.01597	.907	-.0292	.0484	
stancetime	Tukey HSD	darlington	terraces	.06479	.05656	.489	-.0698	.1994	
			ozcare	.03526	.05716	.811	-.1008	.1713	
		terraces	darlington	-.06479	.05656	.489	-.1994	.0698	
			ozcare	-.02952	.04337	.775	-.1328	.0737	
		ozcare	darlington	-.03526	.05716	.811	-.1713	.1008	
			terraces	.02952	.04337	.775	-.0737	.1328	
	Scheffe	darlington	terraces	.06479	.05656	.521	-.0758	.2054	
			ozcare	.03526	.05716	.827	-.1068	.1774	
		terraces	darlington	-.06479	.05656	.521	-.2054	.0758	
			ozcare	-.02952	.04337	.794	-.1373	.0783	
		ozcare	darlington	-.03526	.05716	.827	-.1774	.1068	
			terraces	.02952	.04337	.794	-.0783	.1373	
	Hochberg	darlington	terraces	.06479	.05656	.584	-.0726	.2021	
			ozcare	.03526	.05716	.901	-.1035	.1741	
		terraces	darlington	-.06479	.05656	.584	-.2021	.0726	
			ozcare	-.02952	.04337	.872	-.1348	.0758	
		ozcare	darlington	-.03526	.05716	.901	-.1741	.1035	
			terraces	.02952	.04337	.872	-.0758	.1348	
singlesupportbase	Tukey HSD	darlington	terraces	.03804	.02000	.144	-.0096	.0857	
			ozcare	.02151	.02022	.539	-.0266	.0696	
		terraces	darlington	-.03804	.02000	.144	-.0857	.0096	
			ozcare	-.01653	.01534	.530	-.0530	.0200	
		ozcare	darlington	-.02151	.02022	.539	-.0696	.0266	
			terraces	.01653	.01534	.530	-.0200	.0530	
	Scheffe	darlington	terraces	.03804	.02000	.169	-.0117	.0878	
			ozcare	.02151	.02022	.569	-.0287	.0718	
		terraces	darlington	-.03804	.02000	.169	-.0878	.0117	
			ozcare	-.01653	.01534	.561	-.0547	.0216	
		ozcare	darlington	-.02151	.02022	.569	-.0718	.0287	
			terraces	.01653	.01534	.561	-.0216	.0547	
	Hochberg	darlington	terraces	.03804	.02000	.169	-.0105	.0866	
			ozcare	.02151	.02022	.639	-.0276	.0706	
		terraces	darlington	-.03804	.02000	.169	-.0866	.0105	
			ozcare	-.01653	.01534	.630	-.0538	.0207	
		ozcare	darlington	-.02151	.02022	.639	-.0706	.0276	
			terraces	.01653	.01534	.630	-.0207	.0538	
doublesupportbase	Tukey HSD	darlington	terraces	.00899	.02025	.897	-.0392	.0572	
			ozcare	-.00181	.02046	.996	-.0505	.0469	
		terraces	darlington	-.00899	.02025	.897	-.0572	.0392	
			ozcare	-.01080	.01552	.767	-.0477	.0262	
		ozcare	darlington	.00181	.02046	.996	-.0469	.0505	
			terraces	.01080	.01552	.767	-.0262	.0477	
	Scheffe	darlington	terraces	.00899	.02025	.906	-.0413	.0593	
			ozcare	-.00181	.02046	.996	-.0527	.0491	
		terraces	darlington	-.00899	.02025	.906	-.0593	.0413	
			ozcare	-.01080	.01552	.786	-.0494	.0278	
		ozcare	darlington	.00181	.02046	.996	-.0491	.0527	
			terraces	.01080	.01552	.786	-.0278	.0494	
	Hochberg	darlington	terraces	.00899	.02025	.959	-.0402	.0581	
			ozcare	-.00181	.02046	1.000	-.0515	.0479	
		terraces	darlington	-.00899	.02025	.959	-.0581	.0402	
			ozcare	-.01080	.01552	.865	-.0485	.0269	
		ozcare	darlington	.00181	.02046	1.000	-.0479	.0515	
			terraces	.01080	.01552	.865	-.0269	.0485	


Homogeneous Subsets


meangaitspeed	
	racf	N	Subset for alpha = 0.05	
			1	
Tukey HSDa,b	ozcare	40	.5975	
	darlington	17	.6400	
	terraces	43	.6570	
	Sig.		.468	
Scheffea,b	ozcare	40	.5975	
	darlington	17	.6400	
	terraces	43	.6570	
	Sig.		.501	
Hochberga,b	ozcare	40	.5975	
	darlington	17	.6400	
	terraces	43	.6570	
	Sig.		.560	

Means for groups in homogeneous subsets are displayed.	
a. Uses Harmonic Mean Sample Size = 28.017.	
b. The group sizes are unequal. The harmonic mean of the group sizes is used. Type I error levels are not guaranteed.	


steplength	
	racf	N	Subset for alpha = 0.05	
			1	
Tukey HSDa,b	ozcare	40	.3990	
	terraces	43	.4167	
	darlington	17	.4324	
	Sig.		.243	
Scheffea,b	ozcare	40	.3990	
	terraces	43	.4167	
	darlington	17	.4324	
	Sig.		.275	
Hochberga,b	ozcare	40	.3990	
	terraces	43	.4167	
	darlington	17	.4324	
	Sig.		.290	

Means for groups in homogeneous subsets are displayed.	
a. Uses Harmonic Mean Sample Size = 28.017.	
b. The group sizes are unequal. The harmonic mean of the group sizes is used. Type I error levels are not guaranteed.	


stridelength	
	racf	N	Subset for alpha = 0.05	
			1	
Tukey HSDa,b	ozcare	40	.8080	
	terraces	43	.8386	
	darlington	17	.8547	
	Sig.		.489	
Scheffea,b	ozcare	40	.8080	
	terraces	43	.8386	
	darlington	17	.8547	
	Sig.		.521	
Hochberga,b	ozcare	40	.8080	
	terraces	43	.8386	
	darlington	17	.8547	
	Sig.		.584	

Means for groups in homogeneous subsets are displayed.	
a. Uses Harmonic Mean Sample Size = 28.017.	
b. The group sizes are unequal. The harmonic mean of the group sizes is used. Type I error levels are not guaranteed.	


supportbase	
	racf	N	Subset for alpha = 0.05	
			1	
Tukey HSDa,b	darlington	17	.1424	
	terraces	43	.1530	
	ozcare	40	.1628	
	Sig.		.397	
Scheffea,b	darlington	17	.1424	
	terraces	43	.1530	
	ozcare	40	.1628	
	Sig.		.431	
Hochberga,b	darlington	17	.1424	
	terraces	43	.1530	
	ozcare	40	.1628	
	Sig.		.477	

Means for groups in homogeneous subsets are displayed.	
a. Uses Harmonic Mean Sample Size = 28.017.	
b. The group sizes are unequal. The harmonic mean of the group sizes is used. Type I error levels are not guaranteed.	


steptime	
	racf	N	Subset for alpha = 0.05	
			1	
Tukey HSDa,b	terraces	43	.6451	
	ozcare	40	.6555	
	darlington	17	.6924	
	Sig.		.331	
Scheffea,b	terraces	43	.6451	
	ozcare	40	.6555	
	darlington	17	.6924	
	Sig.		.365	
Hochberga,b	terraces	43	.6451	
	ozcare	40	.6555	
	darlington	17	.6924	
	Sig.		.398	

Means for groups in homogeneous subsets are displayed.	
a. Uses Harmonic Mean Sample Size = 28.017.	
b. The group sizes are unequal. The harmonic mean of the group sizes is used. Type I error levels are not guaranteed.	


swingtime	
	racf	N	Subset for alpha = 0.05	
			1	
Tukey HSDa,b	terraces	43	.4084	
	ozcare	40	.4180	
	darlington	17	.4424	
	Sig.		.192	
Scheffea,b	terraces	43	.4084	
	ozcare	40	.4180	
	darlington	17	.4424	
	Sig.		.222	
Hochberga,b	terraces	43	.4084	
	ozcare	40	.4180	
	darlington	17	.4424	
	Sig.		.229	

Means for groups in homogeneous subsets are displayed.	
a. Uses Harmonic Mean Sample Size = 28.017.	
b. The group sizes are unequal. The harmonic mean of the group sizes is used. Type I error levels are not guaranteed.	


stancetime	
	racf	N	Subset for alpha = 0.05	
			1	
Tukey HSDa,b	terraces	43	.8870	
	ozcare	40	.9165	
	darlington	17	.9518	
	Sig.		.440	
Scheffea,b	terraces	43	.8870	
	ozcare	40	.9165	
	darlington	17	.9518	
	Sig.		.473	
Hochberga,b	terraces	43	.8870	
	ozcare	40	.9165	
	darlington	17	.9518	
	Sig.		.527	

Means for groups in homogeneous subsets are displayed.	
a. Uses Harmonic Mean Sample Size = 28.017.	
b. The group sizes are unequal. The harmonic mean of the group sizes is used. Type I error levels are not guaranteed.	


singlesupportbase	
	racf	N	Subset for alpha = 0.05	
			1	
Tukey HSDa,b	terraces	43	.4037	
	ozcare	40	.4203	
	darlington	17	.4418	
	Sig.		.108	
Scheffea,b	terraces	43	.4037	
	ozcare	40	.4203	
	darlington	17	.4418	
	Sig.		.131	
Hochberga,b	terraces	43	.4037	
	ozcare	40	.4203	
	darlington	17	.4418	
	Sig.		.126	

Means for groups in homogeneous subsets are displayed.	
a. Uses Harmonic Mean Sample Size = 28.017.	
b. The group sizes are unequal. The harmonic mean of the group sizes is used. Type I error levels are not guaranteed.	


doublesupportbase	
	racf	N	Subset for alpha = 0.05	
			1	
Tukey HSDa,b	terraces	43	.2340	
	darlington	17	.2429	
	ozcare	40	.2448	
	Sig.		.835	
Scheffea,b	terraces	43	.2340	
	darlington	17	.2429	
	ozcare	40	.2448	
	Sig.		.849	
Hochberga,b	terraces	43	.2340	
	darlington	17	.2429	
	ozcare	40	.2448	
	Sig.		.919	

Means for groups in homogeneous subsets are displayed.	
a. Uses Harmonic Mean Sample Size = 28.017.	
b. The group sizes are unequal. The harmonic mean of the group sizes is used. Type I error levels are not guaranteed.	


Means Plots


¡îá£§dòÉäää#GJKKWe7ê"R©.¬B>9sæÌæÍÃyïÞ½«²ugÏµdòÃÀÀÀöíÛC·Z[[sö÷Ûñd¬Bîkoo/..®««a.ÿOö°ºèÈ*ä¨ÃG»Qßzë­ÜÙºúúz¬BÎ¹páB*Cê=&&&òåvt[3gÎxAV!'~½¥¥%µ­­-ï¾ýÏ#È*¬²ñññ0&ü=ÔXAVaEg£Fõÿý÷ób7ê"Âö°¬ÂªwõõõÑnÔ53ÑU²+*:55ÌvW®ÿ×¬ «°B¢úehOÿÚû?6VUxæ&''ßzë­²²²d2yìØ±|ßºXAVáÙêíí­©©YK»Qá¢K «ð¬Fg£®½Ý¨VUX9ÑÙ¨kx7ê".``YìHßuÍïF]Ä¶mÛ¬ «°Î©««+Ý¨£jX	VUÈPøÍijj-imm-Ý¨p+È*d"L¥Ñ½Qê¸¤ouöìYXAVa	&''8PZZZUUU°»Q±ûv+²*«ðTÎ9SSSºwïÞ<º7êJrH0È*|»+W®!,£­­mttÔY=¬Èª¬ÂÍÞF1+ä[E÷a5°"«²³Õyz.º¬Ê*<&tÔnÔmsXUY?v£*ØºXÈª¬R¸BDCJnk¬Ê*+½µ¦¦ÆnÔl±YUQz7j(«Ë;XAVeÙú¬9YUÝ¨+ÃUUYe³ÕÀ²*«dÝ¨+ÏVdUVYw»QWsXUYeí°uÕQÕEUY%ï×ÃÞ½£Ý¨gÎ±BVû°"«²J~KßÕnÔXíaEVe¼Ôßß____\ÜÞÞ>>>näð¤XUY%G»QìFÍ5aCc`EVeü¦Ò=ö$èlTïúæ&ç°"«²J®ýðÃ+++ËÊÊÞzë-AÍeö°"«²JNëëë«««+..îìì´5/8YUrÑàààöíÛCPÃÇ+ÄÀ²*«d"L¥»víAÝ¼y³³Q¬ «²J&''zzÉdeeåûï¿o7ªdUVÉÐÙ³gëêêÂóÛÕÕ511aXAVeLôõõmß¾=Ì7ÍÍÍ×¯_·B¬ «²J&ÆÇÇ;::Âsºyóæ.X!VUY%C===¥¥¥DâÀÞõ5°¬Ê*:út*ÛÜ]»v[!VUY%áµ¶³!¨VdUVÉDJ;;;«ªªN8áä+Èª¬PÐ#G$ÒÒÒ»Q¬ «²JN>ÆÓ0¤vttY!VUY%Ã1¥®®.L*Û¶msoT«YU24::fÓ°%Ý´iSoo¯YU2199¹wïÞD"áÞ¨XUYeYAýðÃkjj¢£ÚYU2Ôßß___o7*VdUVYÑÑÑ0¡nÚ´É½Q1°"«²JÂsa7*VdUVÉcÇÙYU+¼¢«äÛYU27<<ÜÖÖf7*VdUVY°Ú÷íÛWZZzôèQ»Q1°"«²Jz«ªªBS>Ö¿YU2Ñ××J¥ÛÛÛGGG­¬ «dâúõëÑnTÇ%a`Y%sa>|8H¸J>VUÖÄÄÄ[ZZ9tìØ±d2i7*VÈ¿¬>xð`÷îÝñx¼ººº¯¯/ýøùóçÃß°²úLa´µµõìÙ³§O®¯¯ßºuë/¼`7*VÈ×¬îß¿ÿÐ¡C333¡©555ÑÓÓÓßÿþ÷eõYÛ±cGGGG´"úWõWaÿÙýÝ¨X!_³ºeË7nÌyðàÁ¿øÅ/ÒYý§ú§?nçÎÏ=÷'u*++ÇÇÇÃÂDø´«««¹¹Ùa=ÖÀ¬fG,çwJJJÂ¨zíÚµðÈíÛ·S©T_ÓYýÇüÇíé¥d5+Yø¯G7I=|øpHì3gdU~¯¬hV×-ª¨¨hI?-|ÿÑ£GÃÂÍ7CMÃBØ¬þùçÑÈÀÏÔ;öíÛ7gÓvàÀk6OV4«E³ÌÏj>ôÓ***fO®ó³-«ÏÎõë×ÃÀÚÝÝ=88Øßßþ 	ÙDó+¬¼¦¦&+Õ´·ß~;dïç?ÿù½G~úÓOßï½%ýÎÎÎS§N¡¡¡9c±iõYÞµkW]]]jWW¦²úúú¬tVãñøòOÄÓ¿>Þ½7IaNõYBf+²º.8SSS<hii	¸``EVìþáæï[ýàdÈØ;¬hVÞÞÞ7Æ©ªª$Y2¶SÅÅÅ§O¶*(Ä¬º&0uMMMuuuÑªAVeÈÂÀzâÄ	«ËêÐÐPmmm"®]ÏAVejnn6°RpYn23ûâJa!ü2È*°Lýýýab`¥°²ºaÃðº¿qãÆì¬>ýy«²,bÇV+«éFY.¿ÔkË*° è*ÁV(«Ï?ÿ|xÑ_¾|9ªi´0û¿²,=¬VVO<9ÿr½½½²dÅàà C) ¬.]ªªªÇã±X,L;wÎÀ@mß¾ÝÀJeÕy«À3500``EVe0°"«.Xaµ²êrÀÊhii1°²ö³êrYu9 ÿ´µµ¥R)ëµUVx`ýôÓO­ÖlV]Xáµ±±Ñz`ÍfÕå 488þv7°²³ê¼U`%577XUY²ÃVÖxVO:L&b±ØÆûúúdx¦ìaeÍfõí·ßÈÒÑ£Gex¦køõ7°²³ÇCG»»»§¦¦îÝ»÷æoOKJJdx¦:::¬¬Ù¬Î¹@«,ÏZtV+k-«¿üå/CGúÓQõÎ;íííÎ[V=¬¬Á¬®[Ô3½¡¬ÕÀÊZËjÑ¢b±¬VdÕy«d0°BþfõáÃUUUÑò÷¾÷½uëÖmÜ¸QV+²ÐÔè¸¤îîîôJµµµ²XÕYÉdòùçðàAÈê3=RIV+k9«¡£SSSácôn°¬Vd5C7nL¿÷ûæoFYÝºu«¬VduÉîÞ½[QQfÖW_5z¤¦¦Æ!KYu``UÍê+ÔÕÖÖVWWË*``EVÕééi,VduÉÂHú¤+ì»1°k½õ@þeõöíÛÑÙ5ÑÀVRRòî»ïÊ*°ë~hUgYM·º2oùÊ*ðôkMMÍää¤UAþeÕÀY]nVÓG*­üÝËe0°²Ö²~ïwåï^.«µUoVX¬NMM½þúë²XÕ%-//Oicß*``EV3Ïêæ¯Çïß¿/«YÍð~«wîÜI&áãÕ«WÃ§²XÕ³ÚÛÛ÷ïß^»Þ¬Èjª««£Þ¾;ý>pEE¬VduÉ¦§§Éd´|ðàÁx<^ZZ:22"«YuÞ*``Y0°²²ú¤k¯_¿>üy(«Y]ÚÀó¯^niiU XÉ¬üìg?»wïÞýû÷ßxãòòò©©©K.¬Ê*#ÚÛÛ¬äzVDtÞêì·CM§§§ÃÂ3½¬K2886YVr:«ñx<ä³»»;L«wïÞáÓ0¿Þ¼y3,W°¬¹c×®]Vr:«ï¾ûîüC>ûì³háW^U wDX>lU£YÎ;WUUÆÖX,¶aÃ>ø z+øÅ_t$0kZ[[+++'&&¬r4«Î[òHtkOOU¬Ê*---Vr4«·nÝJ&ncä×À¶Ç³*È¹¬Î¾yÑÿy¦çÕÈ*°|uuu	&ç²õòåËÞòÈõë×O8aU[Y-//s9YòB»ËêÅCV»ººîß¿/«@~¬.ºDÎeõIw°U ÷¹è9Õ¢8d	ÈÑ!ÁVr(«Î[òû°ëYzýõ×eÈÃÃÃVr(«£££ååå.ä¯ÖÖV+¹ÕÙÄãñ9*XVl¬ÅÅÅVr"«ÑzçÎd2>^½z5|ÚÐÐ «@iiiÙ´ijXhooß¿xQzÈÇ5lOÕÏjuuuÑÛ·o§ß®¨¨U ¿´µµ¹è«Õéééd2-<x0È*_UÎªóV5£½½Ó¦MîÃ¬dÁõë×ÃV¥§§Çª`5³:44T[[H$¢¬©TJV<ÕÙÙYYYi`eÕ²zþüù9WÍÍÍ²äéÀZ\l`eÕ²]âÆ³³&WYòTkkkXÃÆª`²Í©ÑBÈêÌÌóV¼600à¢K¬ZVþùÐÑË/G5·ä5·µaÕ²zòäÉù·1ïííU ¯V·µau²t©ªª*Çb±d2yîÜ9Gù®££ÃÀÊêdÕy«ÀÚ388h+²5ö°²Yýâ/ÜÆ0°"«ÙÉj"H§4-É*``EV,¦×7+²ºÜ¬~üñÇ!«|òÉôô´¬VduYY/µõë×Ï9oÕ¾UÀÀ¬Ú·``eU³jß*``EV³Õwß7dõ³Ï>³o0°"«Ù¹«YÍBVbß*``EV]¼ÀÀ¬XUY¬Èjö=xð`÷îÝñx¼ººº¯¯/<rùòåïÿû±X¬®®îêÕ«²XYËY¯ª-[¶LMMe%«û÷ï?tèÐÌÌLhjøÉáïï.]'O¬­­nóÑã9JìI¬äV£«@D'Ø,ÿtPè7n<é«%%%áãßþíß~÷që×¯ã¬'0°÷Yn°úàÁ¬d5ÔñwÞ	ù¯Ôk×®ÍþÒ_~ùÚk¯y0°²³L&×=ÙRC¾ÿèÑ£aáæÍ©T*ýø½÷ZZZôV³¬VÖHVÃYZZÍ¬s®³Áå ***fO®ÑÂ×_½÷îo¾ùÆÀ5ÕÙæò¯©ÔÙÙyêÔ©°044ÔÐÐúúú^zé¥;wî8Á0°R@Y]¦è¸§»wï677<§R©ðÈÆg¿«,«BÉêåË«ªªbTWWÿö·¿u9ÀÀ¬f¢··wþ!KçÏUÀÀ¬.YyyyèhWW×ÌÌÌôôtXÎ>IV+²º´û­ÎyÄýV+²ù´ÚÙÙ9ýHXnØ°AV+²ºdÿò/ÿ2ßê¹sçd0°"«¸víZuuu</))	/_v$0``EVÝoÀÀ¬XUYÖ«¬Ê*@Ö´··XeUV²cpp0H9rÄªUYÈ]»vUVVNLLX²¡[·n%ÉôW3»¹¬kf`Û¥«BV3´aÃÙ5Íì6æ²¬mmmVY]î5Wì²ä¸ááa«¬.÷Àö­¤µ¶¶Xe5C/^nwÿþYH¬ï¿ÿ¾U!«¾	<C·gÏç°Êj&â%ÀÀH$·``%²:55õúë¯Ë*PàFGGKKKíaÕ¥	¯òòr¯³³Ó!Á²ùå "ñx|eU /Vç°ÊêÒY)½sçN2¯^½>mhhU ££ÃÀ*«KÎjXhooß¿ÿää¤7ÒÆÇÇ¬²ºÕÕÕQDoß¾~¸¢¢BV¬²ºdÓÓÓÉd2Z>xð`<È*@dll,l¬ÜUV·µÕ9¬²*«Ù1>>î¢K²ú´jkkÃ+&ÚÉJ¥d`®®.«¬~»óçÏÏ¹DXhnnU9kii©UVêr7nÜÕ0¹Ê*ÀüµªªÊ!Á²úí7BVgff·°ÈÀêVY]ÌóÏ?:zùòå¨¦ÑóV4°:UVsòäÉù·1ïííUùÆÆÆ¬²ú-.]ºTUUÇc±X2<wî#¤»»ÛÀ*«Î[ÈñññU«¬Ê*@vìÛ·¯ªªÊ9¬²:÷à'q$0ÀâkYYsXeunV É*À"zzzçf5§R©¹_¬kl`uÑ%Yì~p/^¬®®N¿ñ[[[Û××÷ðáCYx÷î5°Êêïß?~üøÆÓ­È*UV3wëÖ­ÆÆF,Xe5ó¬NMM½÷ÞÉd2=­È¯ýkY0°ÊêÓzøðá?ÿó?ÏÞ·ºyóæééiûV¬²ÉÀ%%%/½ôÒèè¨#¬²êrVr «Er9«¬º&0Y0°"«²``UY0°"«VdUV+²*«¬UUUV¬Ê*@vÖW«BVe ;k(kè«U!«²``UY0°"«²XUY0°Êª¬XU+²*«ÕÀ*«²``UY0°"«VdUV¬²*«VdÀÀ¬Ê*UVe ÆÆÆ¬²*«YsàÀ«¬Ê*@v VVVvvvZ²*«Y°oß¾°Ý¶*dUVkbb"¬°*dUV²3°²¾Z²*«Ë5>>H$,«²ÎaUYÈæÀZVVf`UYÈæÀ:66fUÈª¬,×ÄÄDX;::¬YU,èîîÛÀÑÑQ«BVe`¹Â60¬===V¬Ê*@¦:UVe ;BPÝÖFVe kºººÃ*«²!¨VYU¬Ù·o_YY=¬²*«YíauH°¬Ê*@vtww²:UVe ¢=¬.º$«²===aè*Á²*«Y6	UYÈç°Êª¬dÍÄÄDee¥UVe ;¢û°XeUV²`llÌ9¬²*«Y]tÉ9¬²*«Yíau«¬Ê*@ÖVç°Êª¬dÇøøxYYC7«<Ø½w<¯®®îëëz5LÆb±-[¶xQV¤§§Ç!ÁÕýû÷:thff&4µ¦¦&<ÒÒÒrüøñ°ðÞï½úê«aabbbøqýýý²ð¤ÕE7«a$½qãÆìGÊËËCeÃÂÃ7nÜvíÚµn0ÎzRH$ÂbUßyç0ª^»v-zdöWÃÇ¯¿þúß÷ßüÆ´°øÀÚÕÕeU=nÞ¼J¥¢GÒ_Çãö­d6°VVVNLLXÕ9³ixäáÃÑÀ³¿*«KXía-¸¬vvv:u*,544;w~ôÑGa!|liiUVPV×­ûÃúîÝ»ÍÍÍaNM¥R###á/¾øbÃEEEÉdòòåË²°uÏ=VEM«.ðLÖ°µt`YU,K$nk#«²öì±UVe ;FGGÃÀê`YUl¬¿ùÍoúûûÉ*«²°,!¨ßùÎw¾ûÝï666¾vwwONNZ-²*«KÆÓM6mÝº5ºèÒèèh*êìì´fdUVìÄaH½upp0Ì¬VYU%;|øpTÓð1¦MUUYeUV2V·oß>ûÔ²²2Óª¬Ê*ÀMLLÙôÈ#Ñ§aû*ÛÑÑaÍÈª¬dâÊ+Éd²¡¡¡­­-,´´´­¨Õ"«²¡°ÙüôÓOO8100`mÈª¬ «²¬Ê*È*Èª¬ «²¬Ê*Èª¬ «²¬Ê*È*Èª¬ «²¬Ê*Èª¬ «²¬Ê*È*Èª¬ «²¬Ê*Èª¬ «²¬Ê*È*Èª¬ «²¬Ê*Èª¬ «²¬Ê*È*Èª¬ «²¬Ê*È*Èª¬ «²²²*«Èª¬ «²²²*«Èª¬¬¬Ê*²*«Èª¬¬¬Ê*²*«Èª¬¬Ê*²*«Èª¬¬¬Ê*²ºÚYýVÉ/¼°v²úÿùÏ=÷,)***..¶JxÆÃón=xÒÉXîúûû×HVÉ®úúúîînë¡ tvvþÅ_üõPP;¶~ýzëáYUdUVUdYEVUYEVUdUVUdYEVUdYUVÒñãÇÿõ_ÿÕz((öÙ©S§¬ò»ßýîí·ß¶ddd<~.×­Êïyï477[±¹Ì²Êêg5»?ÒYåiõÕW?øÁÖ¯_ÿÉ'D[Ø«W¯¦R©X,VQQqòäÉhËûÁlÙ²eÎ´>:t¨ºº:|ógùúë¯ÃO+//ß¾Ç×=ý^|ñÅðácX^ð³Âf?Aß|óÍøÃðÝºõÖ­[ó÷ôòüWHø·Û¶m?­¶¶öË/¿§?>º|t]]Ý_|aåçÈ¯ÿüßÊÙ|rßDÌêÕÂÒÜÜÚ<ýmÞ¼ùóÏ?£££¥¥¥ÑïLØ&ÎÌÌÌÏêÛo¿Q¿Eáè§íß¿ÎÛÅ¯¼òJô¥ðñå_^ð³Óê~ô£ð,ðìÍâüç=½<ÿþíÏþóðÕðÇYøê?-lÃk,,x±¦¦ÆÏó+Ó>úè£?þxÁ'wñMÄü§Y-¸y%úe¶°áwlçÎá'ÆèæguÎãóZz«þRøüç¬bVÓký3ç	J/Ï¤Üùspú§=z47?ùÉOnÞ¼iµçÚ¯ú·2ÝÔ_üâs¾'mñMÄü§Y-¸ß«À°ððáÃè7ä?øAooo__ßW_5ÿ¥9YóøÓd5|©¤¤dÁÎêfõÁ~iÎòüWÈYóÓ¢95lÃ¶8lµ­ù«:ú­þêêêÿ=¼f¿<|êÕÒÒÒòÆoUWW§7£££SSS¯½öÚR³úê«¯íæïÿûþðÑ#EEEÑïØË/¿~·9ý&°¬®ºôôÊ+¯DoÒ-fCCÃ"OÐüWÈ_þå_FÿöóÏ?Þ'ÿÓÂãÑÇ///·æsÁüßÊ_ýêW?ùÉOfÏü'wñMÄü§Y-,ß|óMcccmmíÐÐPô»~¯Diié/ùË¥f5ü´ðìÆÃ4zO):ØáþïØ¨X,þs_ýµ¬æô°Ëuuu###<Aó_!áyojjOî-[®]»¶àO»zõjøjtì¨¯¬º+ç²4çÉ]|1ÿ©GVÉðj*²dÌuvv®_¿>ü¡ºuëÖ0þZ!²²²È*È*È*È* « « «¬¬¬yãóÏ?n:VRRÒÔÔd¬K&éÛvîÜ¹ÓY±ixdrr2,?|øÐYBâÇ¼~ýú_|1<rûöí¦¦¦X,¶uëÖ/¿ü2ýÍÇ³i<oooNÿó4ëddõýèG?Î~°¶¶6úÎ_ÿú×³óÍ7edX «]]]aylllöÂ§áKa6>mhh~òÉ'###a¡¦¦föO°&AVÿâW_5ûÁîîîT*5;±XlÁ|Ê*È*ðÄ(~ðÁá7Þx#JçduffFVAV§Íj<Ü¾ûâÅ³¿½	|îÜ¹7nÒÒRYY¾%«ÕÕÕéCDøxïÞ½ÿwÈÒ=ddø¬~ùå[¶lÅb¯¼òÊ­[·ÂWö³E_úÕ¯~UQQ`sÿþYYYYddddUUU@V@V@V@VYYYfùúÿ7!ÊIEND®B`


vvÖLdlj:îïï/JBáÀÙlvnnÎddÖÔÔºøÞÞ^UXeee¸Náãàà`ø8::ÚÚÚj2²ÊËd2ÑC¾ÃÃÃaûöí;qâD[[ÉÈ*¬ëÑv¸N#ýó?ÿó·ß~ÛddV(¾ÑßßäÈpñ¦ú7ó7µ «<è¯ªû÷ï?pàÀÈÈÈ+Wª««Ch@VÁÌÌL.«««5Y%²kÖÊÊÊÉÉIÓUVªP(Ô××²;iÈ*+ÜÜÜæ<<<l²ÊJJ¥®®®D"144d²JöïßÊzäÈ£UbL&C_@VÁàà`´Àa´0¬Ê*+222ÆÞÚÚæo¬Ê*+5>>^QQÑÜÜMUYe¥¦§§³Ùlcc£YUb055U]]]WWk¬Ê*+533SóedUVA¡Phll´À! «²J<Âhii	ûbllÌ4YUV*Zà0L:uÊ4Y]Y=öl"ñíÿÉÕ«W÷îÝ[VV¶gÏ?üPV×²º&²:??ÿÔSO-d5Ôô·¿ýmØ¸páÂ;du½8vìX¸f=|ø°Q²º:tôèÑ¬.xÿý÷s¹×@?ú®¿ú«¿zâ'ìÔµfpp0Jíß¿ß¬®ë×¯çóùÛ·o/Îj¸~Ý¶m[¸åäÉáÓ÷Þoÿw½òÊ+²º6²¶¶¶ «« ­­íüùóÿûÿ±ìjõìÙ³;wîô ðº3>>ÊÚÒÒbC@V·Äw-ùjYY¬®G¹Ö4Y]¾FO>ùäÕ«WÃÆÅî9Y]§¦¦¦²Ùluuµ²²úX;ºäÓË/ïÙ³'>ûì³þ¹¬®_ÓÓÓuuu!®8dÕrÄÀ¬Ê*q²À! «²JÌÚÚÚ,pÈª¬¾¾¾D"> «²J<®Yzz,ÃÈª¬PÖÎÎNedUVA´tp[[[Ø¦Èª¬²RÑÒÁõõõ8dUVÁÄÄDee¥YUâ-pXSSc&@VeÌÌÌÖL&c&@Ve|>N§ÇÇÇMUYe¥Åb[[[Ø§adUVA©TeM$Ç7@Ve8p õðáÃFÈª¬hé`Ë0²*«ÄàØ±cÉd²­­MYYUb044vqkk«eYUb0>>nC@Ve8ËZQQQWWg&@Ve VVVf³YedUVÁììl>qµ «²J,pÈª¬§°Ó[ZZÉäÐÐi²*«Ä ­­MYYUb³ÿþD"qðàA£dUVAhj¸fíéé± «²JBY;::UY%©Tª½½Ý2L¬Ê*1÷|>?77g¬Ê*+uæÌÊÊÊ§¬¬Ê*1©®®®©©±À! «²J¢eÂeëÄÄi²*«ÄPÖÆÆÆt:îè¦Èª¬²RáÑÚÚÊzæÌÓdUVAooo¸ «²J;î!á£Q²*«ÄàÈ#D¢¯¯Ï(YUb044î'ÝÝÝ8dUVÁøøx:noo÷ÓdUVY©±±±T*ÕÐÐP(LUYe¥&''3YUâ1;;ÏçUY%Åb±©©)ÍZ	UY%²644XàUY%¥R©££#J «²J¢O:e¬Ê*1èëëK$o¼ñQ²*«ÄàðáÃ8dUVÍÐÐP*:pàY#µ©©©X, «°RcccétZYYxLMMe³Ù|>o&@V!³³³õõõ555ÓÓÓ¦È*¬T¸k566ËÖpñj¬ÂJÅPV²ñ(J©TjxxØ4Yôôô$£db°oß¾PÖ×^Í(Y>|8Lîß¿ß2L¬B?îoÊÈ*ÄàÌ3étº££CYYWTTXàUÇÄÄD6­©©¹råi¬Ê*¬ÔÌÌL.Ëd2!±¦²*«°RB!ÏWVVNNNÈª¬ÂJÅæææt:=66f «²+î---ÉdrppÐ4@VeVªT*uuu%ÃÈª¬B8ÊzäÈ£YUÁk¯½ÊÚÛÛk «²1H&áÎi «²+5<<J¥,Ã²*«|>?77g «²+555Ífs¹²¬>R©TUUµeËÄ"áSYe©ûÆìì¬i¬Þ¯ð+ùâFÊÊÊdB8FÂÅ«i¬Þ(¨ôa¹p/85ÕïW]]²êo«p7¥R©¥¥%ÜWO:e «ßoûöí?ýéO¿þúkY»µ³³3ü:44d «wì÷n<e	;pà@20Õ¥¶Ü§,Á½öÚk¡¬á£Q¬zÝ*Ä Zà°«««T*Èê^üoH]mmmUU¬ÂÝ¥R©ÖÖVK¬~OVçççÃ-ÇaEEEss³²¬~+Þí)Ká7qY8´(?Èê·®_¿¾°fáâç+mÝºõ­·ÞUø^ÓÓÓ555YýÎSÏC¾²ÊT(òù|uuuH¬i¬z&0¬T±XlnnÎd2¦=«w[bÛ¶m²÷#Ü[ZZR©ÔÈÈiÀ¦Îjô÷Ô%M]ØnooU¸Ñá.mCØÔY½zõêÖ­[ö³õÕW7oÞ|õÕWwìØqãÆ.¬¦ÓéûÿQgÏ]XµÿâÅO=õTYYY.»|ù²¬²Itww£ ¿¿ß(`f5J-y¨¦úÖðý¡£?êÉ'a'O¬­­¿þõ¯ÿßwýýßÿyy¹ÊL&ûúú6cVCØBÃÕêÿøÇ°>×¯øÃè¬:zôèßc.ü´ðñõ×_ú»òùüO<a§²ñþð?ììì´À!lº¬¾õÖ[Ë²ôÁD/¼ðÂ¾64òöíÛË³zéÒ¥ÿøÇf³UVØYN>ÍfÃekYYÙ®]»Þ÷Ýè¡àgyæ>B[[Ûùóç£µøöpÜÞÞ~ãÆYeÓµ©©©P(l¢¬®ÜÝèÆÏ>ûìW^ùüóÏ=Mkll¬²²Ò2L «ß×hc||ü¹çûòË/½ÀMnff¦ºº:5llü¬^»v-É,éêvtÉ§»wï^~ý*«lNáR5Ï§Óépôlð¬îÚµkqM#ÞâU(êëëS©Ôèè¨iÀFÎjÔ/Z©hÃd2yüøqÓÕ;vÜíAZYØËÚÚÚª¬°³zîÜ¹Õ7oÊ*<ápÝ#G6`Vïö6²ÎáÃÃ5kè«QÀFËê;ñ%xÔBY-Ã-«ÞÆVËÐÐP8Z[[Ã±` «²+-pØØØh&Ø8Y½zõjmmm*þ¤Ïçe±±±ºººééiÓuÕè½Ç?S)l´µµÉ*<6Ùl6É(+¬û¬F«,üñÇ³zÿo³*«hÃÊÊJÂúÎêÂ½QV£÷LõxüÅbsssø¥vddÄ4`½fuûöíÑâQM£;wÊ*<~Ñá¸PVX¯Y=yòäòå eVE©Têììa]f5¸páB6-///++Ëd2§OöL`X]ÝÝÝáÜ¾¾>£õU¯[5(45uß¾aYUA8FÖAV÷äÀ°FDË0µ··[àÖtV·Ü¥öaí8sæL:¶À!¬é¬zÖ§¬ «²1ª¬¬¬««1XÓYýÕ¯~Éd¢Ç~wïÞ=>>.«°]¹r¥ºº:ÍÓ5Õ7ß|sùSeÖ B¡P__-p800ÐÑÑÑÖÖÖ××n7XY-//ííí½qãÆW_õúë¯O·nÝ*«°6J¥üàO>ùä©S§FGGCYs¹X4X+Y]òÚï`kÙÛo¿½ûöd2988Ý.[zzLV?«ï¼óNèèOúÓp©úå_FK¦YÖ²ÑpÚÕÕÖþþþÿþæ©Âù|Þd`õ³ºëBÈ*<öööè-nÂjôd+W®är9ÕÏê*®!«ðp<ØÒÒm÷õõ%É_|1¬~V½nÖb±X__®Y'&&Âuê3Ï<óüàwÞ1UYpÚÐÐËåÂuêßýÝß£ixxØd`õ³zõêÕÚÚÚT*ý%5ÏË*¬;===á5XÍ¬=vÉÂF[[¬ÂºÓÝÝ~?ç£UËê®]»BG?þøãÅYõºUX§ººº***¦¦¦V'«Ñuj´²zûömï·ëZ:¶n0¬NV·oß:zñâÅ¨¦ÑÆÎ;eÖ©pd577[V'«'OU`]+J¡¬LfvvÖ4à±f5¸páBøÅ¶¼¼¼¬¬,§OöL`XïÅb>¯©©ñþ¬ð¸³êu«°!PÖ777gð²ºä	J!uµµµUUU²Ê²ºÒ¬ÎÏÏ[éRÀ²Óììl6mhhðVçðh³.IïöÆ5^·ÉÌÌ²Â#Ïêõë×ÃuêÂúJ¶nÝúÖ[oÉ*lÈ²ãÎ4àduááÏC¾²«kjjª¢¢¢©©IYáfÕ3aóemmm-J¦$«·nÝÊf³ÑöO>H$vïÞ-«°ËN§[ZZIVCS£g÷öö.<e©¶¶VVa£^gg§²Â#ùÛjÕL&³ûö¯¿þÚl@YAV>«¡£7nÜ£Ge6OY÷íÛ§¬ÈjYÝ½÷Âc¿¯¿þzÕ½÷Ê*lxÉd²§§Ç(ÕØ²úÇ?þqçÎáõ¥^n©®®ö%Ø$BYûúúYõ GI$½½½F¬Ê*²;vÌ(UYbÐ××ÊÚßßoÈª¬1^¼>00`ÈêgµººzÏ=7nÜU`AWWW26dõÁ_K¿ßª¬²sddÄ(Õ^"ZSIVÅ:;;Ã5«²"« É$îîñVVam*JÍÍÍáð3dõ¾t)NßñmÌÛ;°Ê*¬YáðegpÆ1dÕÛ1µ©©)ubbÂ4U/°VªX,æóùÊÊÊééiÓ@Vï×Å³ÙlÙ7ªªªþýßÿ]VH¡PeÍd2Ê¬ÞáááåOY:ö¬¬eÍåráïÙÙYÓ@V¿Ç;BGzznß¾=??6Â§;wîU`ÁÌÌLÈjëÜÜi «÷].¹Ål%¦§§£²WÓ@V¿çjuÿþýóßáÓ]»vÉ*°ÄäädEEESSS±X4dõÎþå_þeùßVO>-«ÀrSSSL&Ï++²zWôQUUUyyùÖ­[ÃÆÅ=øÞkÖp²êu«ÀJMLL¤ÓéÖÖÖR©dÈª¬+uæÌpüvtt(+²*«@FGGÉd»²"«²Ä`hh(ÅÊ¬Ê*Û5kOOQ «²Ä`pp0µ··×(Õ?]»v-É,¼ñª·1ÂáÃÃ©CYÕ?íÚµkqM½9ðp<N#Ç36uV£ >¶% d6°hõÓþþ~£`óf5ZØßVXôõõ%ÉãÇ4«çÎÞîæÍ²¬Ü¾ûÂYe``À(ØYMÜ§,+ÑÕÕ®YGFFMÕ-wâ)KÀµ¶¶üÌ3FÁæÊª×­B8À[ZZR©Ôèè¨i°©³zãÆøU`åemnnN&ããã¦ÁfÉêììì;,<ºkÖt:ÎÁ¦Èêâå "åååçYÁ²¤¬ù|>õÊ+¦ÁÆÏjtúå_f2ðñòåËáÓYâR(BY³Ùìôô´i°)²6º»»þóJ%±«®®e16rV«ªª¢^¿~áqà;wÊ*¯ÔºoÄ6«óóóL&Ú>tèPyyy:þäOdxD×¬¹Ù°YõºUà1_³f³YeEV¿ßÙ³g¯Úÿå_îÞ½[V%®N§Õ«W¯ÖÖÖ¦R©è¬ù|þáL~ê©§²úáÖÕÕ-®ìäää¯¿ëÿùËËËíTØ&&&***Õèsñ*a£­­íAÎ¡C=ºÐÑçþO>YÕW^yåÛ©°ËZ___,MÕh9?þxqVÃëýë×¯kÜÛ·o/yëÖ¿«AYCYÂÙÀ4Ø Y®S£Õ(úºÕpuþüùåUàËN§K¥i°²ºûö¿/F56ôu«K?Uàþ9s&:::Õ'O.óááá¾öuµ<¨¡¡¡d2ÙÙÙ©¬¬û¬.f³åååeeeLæôéÓquTVû~eÝ·oQ°î³j9`-eííí5dUVeeg5qOÞÁX-o¼ñF8<xÐ(XYÝreee²¬nYûûûuÕòòò|>ÿxÞ¯FVû×××§¬¬§¬ÎÏÏ;w®ªªjáßÚÚÚñññ[·nÉ*°8p@YY7Y]póæÍ'NìÞ½¡¯¡µáYV]WWW8)ë&«®]»ÖØØè)KÀÒÝÝL&GFFõÕ7n¼ýöÛLfájµººú7¿ù¬kDggg8W9sÆ(X»Y½uëÖoûÛÅ[­««÷·U`M)J---étzbbÂ4X£YRºuëÖçnvvÖ3µ,.+++§§§MµUËAë¨¬á5Í^¹rÅ4XsYÝrOÖ R©ÔÐÐ ¬¬Å¬ZXÅbSSSMMÍÜÜi «²ÄPÖ|>ËåYU B!÷eEVeÁììl6UVdUVxÌÌÌ²666i «²¬Ôôôt&ijjRVdUVLMMkÖ¶¶6eEVe§¬­­­¥RÉ4UeíììTVd ²¦R©ûö²ñññt:­¬È*@<FGGÃ¥¯¯Ï(U¥R©Ã²ãÇ'Éþþ~£@Vb000ÊzìØ1£@VbðÆo$S§N²[YYA___8Õ²ÞÞÞp¶9sæQ «1èêêJ§ÓájÈ*²"«²¬1¡¬W®d`¥J¥Rssseeåää¤i «+Î<¡¬LÆ5+²OYjjjfggMCVe`¥Åb>Ïf³sss¦!«²°RB!5Ë)«¬Ê*@<eÍ#¬Ê*ÀJÍÌÌd³Ù¦¦¦pF2YUemnnVVYUxÊÉdZ[[K¥iÈª¬¬ÔäädEEEKK²Êª¬Ä`bb"µ££CYeUVâ)k*êììTVYUSÓ¾ûBVe ###áìÔÛÛk²*«ñ5L*«¬Ê*@<CY9b²*«1eíµ×BVe ýýýD"|4YU<x0õøñãF!«²¾¾¾p¾6YUtww+«¬Ê*@lºººR©T8¬Ê*@le1YU´´´s×ØØQÈª¬¬T8w577§ÓiËª¬ÄSÖÆÆFeUYG¡PÈçóÓÓÓ¦!«²OY³Ù¬²Êª¬Ä`vv¶ººº¦¦fnnÎ4dUVVjff&ær9eUYÁôôtee¥²Êª¬Äcjj*É²EÓUYá5µ©©IYeUVb099YQQÊZ*LCVe`¥&&&BY;::UVe cccáäÖÙÙ©¬²*«1QVYUØ'Éûö¬Ê*@BYûúúBVe ¶²<xÐ(dUVb+køh²*«18vìX"èïï7YU<xPYeUVbÓ××Ê:44d²*«1èîîç½ááa£UYAWWW8õ¬Ê*@Z[[Óét8¬Ê*ÀJ³²Êª¬Ä©££CYeUVâQ*+**®b²*«+N¡¬ÙlvzzÚ4dUVVªX,æóù¹¹9ÓUYX©B¡PWWÊêÑ`YUÌÎÎ²f³Y×¬²*«1A¬¹õ[gÏM$¾ý?¹|ùr&)++Û³gÏ¹sçdàMOOÖ|>_,Mc³gu~~þ©§ZÈjû'ÂÆÛo¿ýÒK/ÿüÏÿý®û·U%e×$BÁ46uV:tôèÑ¬îØ±ãöíÛaãÖ­[»wï]]]eÂå¬°ØÔÔT(k.sÍºy³zýúõ|>:ºÕÐË¯FÛ~úéï¿ëw¿û«U;^³VVV677ñÅ¦±³ÚÖÖvþüùÿýÿø¿¬nÙ²eá«åååþ¶ð@&&&Òé´²nÒ¬.yh7Ü²sçÎ[·nEmYxPccc¡¬ííí¥RÉ46WV÷5ÚxùåùË_ð1Ü'dà!ódgg§²n¬.ttÉ§~øá®]»¶lÙÉd.^¼(«çÔ©SÉd²§§Ç(6ãÕªå b722J¥º»»§§§9nqý*«²ð?¾eËòòò¾¾¾|>ßÐÐàµ­²*«cnnîÏþìÏDoootKggg[[ÉÈª¬<°¡¡¡púÆo$Éþþþÿþæo***<,«²ðÀ9]§èÆl6;;;k8²*«fxx¸¾¾~ñµéÔÔ«UYUòÏç»»»£§)MLLär¹Ã¬Ê*ÀÃAmmmM§ÓÙl6É;vÌLdUVVzÙêÎeUVUY@VeddUVUY@VedUVUY@VeddUVUY@VedUVUY@VeddUVUY@VedUVUY@VeddUVUY@VedUVUY@VeddUVUY@VedUVUY@VeddUVUY@VeddUVUYYYUdUVUYYYUdUV@V@VeYUdu]eõSX%ù¹q²ú_ÿõ_O<ñD1Ù²eK24M%ìñ°ßÍÁNç¡«»UâU__ßÛÛkÊþýûô£Ã¦rüøñmÛ¶Ã£&«Èª¬"«È*²¬"«²¬"«Èª¬"«È*²¬"«È*²*«<N'NøÝï~gÊ|ð«_ýÊ6ßÿþ÷o¾ù¦9È*È*È*ëx_&÷ù=÷ó¶¶6]Ëì UV?«ñþLÖøNdûõé§>ýôÓÛ¶mÿý÷£3ìåËóù|YYÙÎ;O<yß÷Ý=ö,¹Zÿéþ©ªª*|ó|nùì³ÏÂOÛ±cGøþðÕòòòÄ7¢ÿÐ3Ï<¾3|Ûwüç<fwÐçþì³Ï±wïÞk×®-ßïÛËï!áß677V[[éÒ¥;þ´³gÏFËGçr¹?üÐð×Èá¿ü¨;÷Þ§å»YÝÚÚBÛÂÆ¡C¢£¨®®îüùóacvv6NGÇL8'Þ¾yVß|óÍpb8Â-íííÑOûùÏ¾äáâ^x!úRøøüóÏßñ³W«/¾øbØËa#ìýpZß¶ßCÂ¿ýÇüÇðÕðËYøêZ8/ûXØ8wîµÉ¯ËÊ¿üå/ßï½;îÜ"ïzduÓ]¯DÃüü|t-JáùåÃÁ³ÆèguÉíËÚÂYáKácØ¾ã?g³ºpåD¿å,ÙAÛËï!;wùuðÂO7?ùÉOþð?ûZ;üÊ¦=ztÉ÷,¸÷)bù®GV7Ýq6nÝº!O?ýôðððøøø§~ºü	JK²ºäöûÉjøÒÖ­[ïøÏYÝ¬~ýõ×wüÒíå÷;fuÉO®SÃé8ÃYÛä×ÚoÕÑQýÔÓÓ³üîqX|÷¸ã®GV7öööW_unn®ªªjá9;;ãÆÿøÇÕ^z)7¿øâg6ºeË-Ñ1öüóÏ/<Ú¼ð °¬®ºôÂ/DÒ3fCCÃ=vÐòÈßþíßFÿöüùóÑãËZ¸=zlðÄ;vì0ùµ`ùQù_üâ'?ùÉâïY¾sïX¾ëÕÍåóÏ?oll¬­­½zõjtlã*J¥ÓéwÞyçA³~ZøMv÷îÝá=¦=ÙáOÿ÷Ü¨²²²ðûì³ÏduXØAa§`ØÎår|òÉ=vÐòHØïMMMaçîÙ³ç£>ºãO»|ùrøjô&ØQ_Yuw<*?eiÉÎ½÷)bù®GVAøE5Ï¬òðöïß¿mÛ¶ðêÞ½Ãå¯È*È*È* « « « «¬¬¬²²²¬çÏÞtlëÖ­MMM²<¼L&³ð¶/¿ü²¬+85|£T*í[·nÈ*lö(¾÷ÞÛ¶mægÂ-×¯_ojjÚºukYYÙÞ½/]º´ðÍ'N×¦åååÝÝÝóóóÿ|y¬¬~ëÅ_·.¾±¶¶6úÎßüæ7oýõ×ed¸CVzzÂöÜÜÜâ/OÃÂµiôiCCCøôý÷ßÿäOÂFuuõâ` «À·QüôÓOß855ÕÛÛÏç'³¬¬ìùUUà®Q|÷ÝwÃ-¯¾újtUº$«·oßUUà~³Z^^n¹~ýú¹sç5zøôéÓüqØH§Ó²²|OV«ªªJ¥ÂÇ¯¾úêOË²tàÀYY¾'«.]Ú³gOYYÙ/¼píÚµðÕýìgÑ~ñ_ìÜ¹3zÍÍ7edddUUUU@V@V@VYYYYdddXäÿõ1ÅTã©IEND®B`


áÆ±cÇ¬¬2gD"zË7L¨ñx|llìÐ¡CÍÍÍV@V³Í_7c±Ø¯~õ«d2ÙÓÓced9ãiý×Þ÷Ýþô§ßûÞ÷~ñ_Do «ÌYè¾ûÚÚÚ¶nÝÖk «dÀ-[ÂªYYe¾BPÃÀÆVK «d@»@VÉè`XdÌM-//¿û¶¥Uækll,ïÚµËRÈ*ÐÕÕe`U29°nß¾ÝRÈ*ÐÞÞ%ØRÈ*óÜÕÕe)d+¬Êª@Ve5_¦XdÌ	6°È*VYÕ,sûöíÕöövK3Y=uêTAAÁÃïÕÅâ²6¹ÕÉÉÉµk×NèÌduE»+@nduçÎöìÑi÷ìÞ½û¹oúñüÄOØ¨¦££ÃÀYM&<HEtæ=lû¦W^yEVCr#«ÍÍÍçÎû¿ÿÿFtæ=ÞÎ.kY-ø¦YïÕ,XKKK] «³:µ¯ßy¬.®îîn+@fuZ5e5û Æb±mÛ¶Y§ @VÉÈ*ã,Á²JÆDaíèè°¬Ê*XÃúXYU26°ÚÃÈª¬±5uddÄR²*«XdUV³l`---µUY%c«CYU26°:$UY%«=¬¬Ê*Xc±ØÐÐ¥dUV1°Èª¬fÙÀê`@VeL¬	dUVÉäÀzûömKÈª¬ÕV@Ve¬	dUV1°Èª¬fåÀÊê`@Ve¬	dUVÉØÀê`@VeL¬ö°²*«dl`uH0 «²@VÉÊÕ!Á¬Ê*XÛÚÚ, «²JfV²*«dr`µUY%ck,¶¬Ê*XwíÚe)YU23°²X@VeÌ¬íííUY%3kØXV@Ve¸ûv"pH0 «²Jftww;é «²Jfíe`dUV1°È*VYÕü¶wï^+ «²JfWUUXYU2£§§ÇÀÈª¬b`U¬²*«VYU¬¬Ê*VYµQ¬²*«XYUÃÀ:<<l)YU20°VTTlÚ´ÉR²*«d@wwwaaá+W, «²Ê|Eµ1°²*«XYU¬²*«VYU¬¬Ê*VYÅÀ «²UYÅÀÈª¬zzz¬¬Ê*]ÖÆÀÈª¬b`dUV1°Èª¬XdUVyÔµ¥¥ÅR²*«dl`²¬Ê*XíadUVÉäÀ:00`)YU23°nÝºÕR²*«dl`uH0 «²JÆÖÖÖVKÈª¬5lèk×®Y@VeÌ¬ÍÍÍUY%3kAAXY½zõjuuu,[¶lYø2LÊ*c`õV ÿ³zêÔ©ÿ²n477Ë*c`¿¾Èç¬®2<Ùúé§S³&WYåq¬7o¶@>g5S£!«<HõUVÉøÀZXX888h)¼ÍêO>:zñâÅ¨¦Ñ+VÈ*i`mjj²@ÞfõðáÃ3ôööÊ*o`uÒ% o³p¡¢¢¢¸¸¸¨¨(H?~ÜÀ<ÖÕuX|ÎªÏ­²ð«ë°²*«dl`õV ?³zþüù²²²eËMÝ·êH```uV ³ÅR)M)**U÷Àê:¬@fµ¸¸845<ÍyXä[V<²zâÄÉÉIYeV×aò-«áÙmùòåÓ>·jß*V@VyßjtÖþÔUqÖ­[~Î5kÎ?/«|ÛÀê:¬@^e5SûV'''×®]Êj¨éÇk¢¬¬,ÜøÃþð?ßôË_þ2ü¯Û¨K|`uV ¯²úÎ;ïçµÓ§OÏsßêÎ;÷ìÙÊjÊ'jkkÃ;v<ýMÉdò'°Q¬>ÃäOVf3×«£££¡ÑÕo¦Î¯Ñ^ÛÃ¬®ÃäIVÍf®ûVÏ;Ezæ×o»¬òO×aò,«1mØöÝo´¬XÈêìn<õÔSW¯^7.^¼øüóÏË*ß9°:$Èá¬¦v Îsßê´Ù4õååË×¬YæÔgöÎ;²Ê£¬	r5«©¨Ù·êtdd`uH0«Yua8²s`µÈí¬NË7¤®ººº²²RV1°²:ß¬NNN¼	Õ¹	#iÁ·Åb²Õ¹)Ì©3Ï³_RRòÎ;ïÈ*V@VÓ9ËÒÂ¼å+«XüÏª#1°²úØOµ¿|ùò01È*2°ÖÖÖXÌj´?uZSS·[ZZd×ÛÛk`r2«W¯^-))yýõ×¿üòË÷î½ùæeee.YÇã²Ê¢H&V ÷²Åf×7Ôt>À*«X|ËjqqqÈggggV¿øâp#|æ×Ï>ûìqUV1°ùÕwÞygæ!K§OnlÜ¸QV1°²:Ç¯¨¨ckQQÑÊ+ßÿýè­àgyÆÀXYõ¹U¬²3TTTXÉê7ÄÌ®Ê*Y¢»»ÛÀäLVW®µ¦#ßðÊÏÀäFV£ ^¼xÑÀXYoVËÊÊ¦BV1°²¦³gÏ¬vttÜ»wOV1°²úX®`#«XYMó6Ó8d	+ «>·J>¯ªª2°²*«dFOOÈÆ¬¦v Ú·Õùf5µÕ¾U¬¬z+@öeuÅííí·nÝU¬¬fìs«Ë/ojjU¬¬¦éîÝ»~øaxJõµ¨¨híÚµ²ÕôñÅ'N.hãH`rb`mii±@veuÇÕÕÕ±X,Uãñø5k>úè#Y%'Ö¡¡!KdQV£ôöö:dÜX[[[-EY5ãiRS.ÕÔÔ|ðÁ²JN¬á¶¿¿ßRÙÕ'N$	ûVÉ­uóæÍÈ¬¶½÷ÞSÏ[[+«äÊÀZXX800`)¬ÈêÔÏ­¾øâ7nÜ°oXdKVW®Æo8Ë¹>°ú+YuN`òc`mnn¶ÀâgõþýûÑí§zª  `ÕªU²JÎ¬áWwppÐRÕÐÔè¸ßÎÎÎÔ~ÖêêjY%çVXÅÏjtÕp#H<ùä_õUtZ`Y%Öðð°À"g5<MLL?£weÜXt	Xä¬®Zµ*õÞï;¢¬®[·NVÉÅµ°°ÐIÅÌê_|±bÅ0³¾ôÒKÑ=á%¿CÈÝµ©©ÉRU°!ÿV']d26°nØ°ÁR²Xt	UÈØÀê¤KÀf5<ï¬Y³fbbBVÉ×Õ!ÁÀÂeµ¸¸8<ïD§Y««Ê*<°:é°pYÎSIVÉ×ÕIÊj"(øvZYåq¬>Ã,PV/]ºÇ£5êèTN^H~¬Nº,PV§¾¼0Ue`õV`A³êH`ò`uV`¡³zñâÅ¢¯UVVþå/Uòi`µX¸¬öööÎ<déÔ©S²J>¬'O´ÀBdµ¬¬,<éttt<xð`rr2Ü_®X±BV1°²:gÑx:í°!ÿVanZmkküZ¸¾r¥¬b`duÎþøÇ?ÎÜ·züøqY%ÿVµVO>ù¤²²²¸¸¸¤¤$Ü¸xñ¢#É?ÉdrÓ¦MÖxìYõ¹UÞÞÞÂÂB+ «``dUV1°²*«XYU0°²V o²zãÆD"ºðªËc`d5ý¬®rjM]Æ+ «ó='ðBV1°ùÕèÀö­b`d5Y=ölta¸÷îÉ*V@V3ð&ð4YÂÀÈj:ÍÆ!KXYõ¹U0°YÕ7ÞxCVY:ëÀÀ¥YÍLVËÊÊ%«¾¾~ýúõÖd5ó§/ÌQÁ²J68yòdøµZÕÌ²SîÞ½H$Â/__×ï²ÊÒ±þkÖd5cY7¶lÙòÖ[o+ «iª¬¬":::zxÅ²Õ9L$Ñí;wÇãñë×¯Ë*V@Vn¬¬È¬^½zµºº:E;YÉ¤¬b`d5§Nvp£¹¹YV1°²æé >ýôÓ©YkÚn_¼xqíÚµEEEµµµ/_U¬ÀÈj4§F7BV<xÞçV'''CGSYê©§.n>|¸ºº:ÜøÃ7ýîw¿+..¶Q1°ùÕ'|2<á2ªit#Ï­îÜ¹sÏ=©¬NURRþ|åWf½È¬iræeÌçôCFGGÉd4éNûÖ¥K^õUoc`DV.TTTÙ1H?~|®?¡¹¹ùÜ¹sÑ;ÉSïÿòË/[ZZ&&&d+°T²:ÓÝèÎ[·n½òÊ+wîÜq$0V@VÓìkt£¯¯ïùç¿÷®Ø»kýøø¸¥Yó|9Í£	<í]ßÔ«V­9¿Ê*97°dõQ³ºì[9XAVçÕâââd2¹0×«Urt`=rä¥Yýî³7=¶²²2õ>muuu__ßýû÷e¦¬ÖdõQÝ»wïÀSwÖdRëÑ£G-ÈêÜÜ¸q#¼0ë!K²Õÿobbâ·¿ým"HM«UUUô¬ÂÔõØ±cdõ[Ý¿ÿã?ºoµ¦¦fppprrÒ¾U9°:éÈêw	òüóÏ;¾s`=sæ¥Y¼§UÎÀL&duvËÊé ÀÀ²cdXtÅµ÷îÆÆÆÚÚÚþþ~"«²JÎ¬.kÃbikk«©©9vìØµk×º»»ãñ¸·OdUVÉùÕ!Á,0&Ô=Z½zµUY%çVaeáíÝ»·½½=ÜØ¿V£;ËËËoß¾mqdUVÉíµ±±Ñ:°ðYmmm¿~áisß¾²*«²J^¬½½½ÆÓ7_¼çnpp0º³§§ÇÀ²*«äÉÀj+c|||ûöí±X¬ªªê¥^ª¨¨x÷ÝwÃk»öööD"z`dUVÉÕ!Á<na5Çã!¥Ñ'»9²iÓ¦6ttt¤ö°"«²J>¬õõõÖÇ$<Ñ'Éðê­³³sllÌÈª¬b`tr%Ì£a$õ¯¬Ê*Kk`­««³dJxJìèèÏµµµGµ ²*«,ÅÕ<Áü÷ôô$ÒÒÒýû÷;A¦¬Ê*Kw`u`æ)¦555aTµUVek÷ëHOCCCøjmm½råUYa%CCC!¥aB­««sº|YU0°¦ñññÎÎÎX,V^^ÞÝÝm¬Ê*Ì2°644X¾SooïêÕ«Ãó^(«sùÊª¬ÂÃVaå!ÂwxùUXX¸uëÖ¡¡!"«²ß1°ÚÃÊ¬®]»ÖÒÒ~C|KVe¬¤ill¬½½=:K¾KÉª¬4ïÞ½;¦nß¾=<×YYU0°£GÖÖÖnÞ¼ÙEfdUVÁÀJ¢Ó;?Ãm"«²VÒ122²eË0¡®^½ÚeUVÁÀJÆÇÇwíÚÇDww·Ý¨²*«``%MûöíãiRÛÛÛ%_Ve¬¤éÌ3uuuá%TØÖv£Êª¬4E§w¨­­íííuR_YU0°ðµmÛ¶ð¬%ßnTYU0°0öôô$	»QeUVÁÀÊ|_$ÕÔÔ×I6mr±qYU0°¦ÑÆÆÆ°ëêêúúú,¬Ê*XIÇØØXtzÇ%Éª¬tnß¾=þµ®®.UY+i:zôhUUUR7mÚ4<<lAdUVÁÀJ:Â §wUY+ó¦Ò0	5Ì©Î/«²VÒººº¢Ý¨÷îu¬Ê*XIÓþýû£Ý¨Nï «²VÒÔ××íFmiiqzd¬¤éÚµkÑnÔºº:¯rU0°¦±±±®®®X,æôÈ*XI_tüòòr§w@VÁÀÊ|×¼¦¦ÆéU0°2/W®d¬ÌËØØXGGÓ; «``e^ÆÇÇ÷îÝëôÈ*X¯ÔYòÞY+éÜ°aÓ; «²y	Si[[[x©©©±Èª¬b`%MÑnÔòòòÒÒR»QUYÅÀj`MßÑ£GÃx=ÚÛÛíFEVeþ3°Ö××³æäÚµkÍÍÍÑnÔpÛ «²ßXÏ9c)ÅÈÈHMc±X]]]__AVe¬éëÓÝÝ]^^^QQ±oß>Ë¬Ê*<l`=rä¥ø6ªªªCêöíÛ%YUøîÕ!Á³ÏtáeÇ-[%YU0°¦)D´µµµ°°0¼àèïï· Èª¬ÂÜÖ0Y <'¤.6^jØ¬Ê*¤9°ööö.ñuv£çmÛ¶Ù¬Ê*Ìk`]ÊXûúúÂÿýÂÂÂööö¿Èª¬BÖ%xqÐÔnÔ¦¦¦ÁÁA¿	Èª¬BÆÖÚÚÚ¥³7qll,Ì¦ÑYò]lYU0°¦)¼tèééq|dUVÁÀ:_½½½Î¬Ê*Xç«¿¿¿©©):KþÐÐÍ¬Ê*,ÄÀg	Þ´iSaa¡³ä#«²0°æÍgX£Ó;Äãñýû÷Û¬Ê*,´¦¦¦üXzzªªªBS%YUX4ýýýÊéÿ6lÿ/¶nÝêôÈª¬Â"knn®««ËÅÿòááá¶¶¶Ôd2900`S"«²Ù2°æÖem¦%ßnTdUV!ëÖÚÚÚ¯íééI$ñx|×®]v£"«²Y:°fÿ!ÁÇK&ÑnTGVe²z`­©©ÉÚÿ¼ÁÁÁð_ºaÃgÉGVerc`ÍÂ.¥>zæÌ[YUÈ5«Î%¿´´Ô§QUYX³ä3¬'OzµO£"«²Öy¹råJCCCAAASSS¸m£ «²Öt¥Î¦UY]P§N¯gS_Þ½wÕªU²óXÉä¬·oßN´oß>§w@VÚäääÚµkSY=þ|MMÍÔÊþýïþ¦?ÿùÏ²8°.äI¢ÿÑ¶¶6Ç%!«cçÎöìIuô^¸~ýúÔ¬nÞ¼¹`¢¢"e`­¯¯_766Ç¦O£"«itt4L>xð`jGÿï?kÊ7oÞüë7ýéO2­Â£¬uïæððð-[¢³äû4*²ºÈY/¥Ï;7­£3¿´oÒ÷®ÃÛ¶mÅb¥¥¥ÝÝÝv£"«ÕioíÊ*dÜÐÐP%3>G:t(ºØxWW×ØØuFV³ë6¦Ux|6mÚÁë°B¯_¿>¤zóæÍNï¬fKVÞQYºråJxÈ§ù¾¡ÐáG566:½²êt°¤ÖùìaSi,«ªª:tèÝ¨ «°¤¦·5´»»»ôk.6²üÇ-[êêêæ4h;v¬¶¶6<Ü%døèàGüëÀÀ@t±ñ¦¦¦ð­È*0Ý£ìaSi[[[jUUUoo¯EYfwíÚµX,ömXÃ#«»»»¼¼<;½È*ðHk]]ÝÇ&ÑPÙÔýÑéÂÚÑÑáô «À#Ù½wAAAkkkJ·mÛvòäÉÚpg¸Ç§QAVGÕßß_^^þ³ý,ÚÃÚ××÷ü 5dÕYòAV¹ÙºuëöíÛBJ×¯_Ga`­®®¶dUVaÎ¢Ø´¶¶²vvv^¾|yõêÕVdUV!iu×®]ÿüú3¬Ñ§Q9ÒØØhe@Veæ,Ú·Ú××9<<èÐ!+²*«ÑÒÒÒ¦¦¦ÖÖÖp£««Ë¬Ê*¤ïöíÛÇ;räÓ¬Ê*²*«Èª¬¬¬Ê*²*«Èª¬¬Ê*²*«Èª¬¬¬Ê*²*«Èª¬¬Ú¨Èª¬ «²²²*«Èª¬ «²²²*«Èª¬¬¬Ê*²*«Èª¬¬¬Ê*²*«Èª¬¬Ê*²*«Èª¬¬¬Ê*²*«Èª¬¬Ê*²*«Èª¬¬¬Ê*²*«Èª¬¬Ê*²*«Èª¬ÀÉêMX$?úÑò'«ÿøÇ?xâ"2dÙ²eÖaI	[<lwë`£¶0Ýõ÷÷çIVÉ¬ºººÎÎNë°¤´µµ=÷ÜsÖaIÙ¿ÿòåË­Ãã&«Èª¬"«È*²¬"«²¬"«Èª¬"«È*²¬"«È*²*«,¤üéO²KÊéÓ§?üðCë°¤üõ¯ûí·­¬¬¬ÃÛ² àÿÎ£üÍHss³Íf6È*ÕÌþL²|£²Ê£ºyóæÓO?½|ùò'NDÏ°/_N&EEE+V¬8|øpôÌûþûï¯Y³fÚ´þüÍo~SYYþòéÓ§Ã=·nÝ?­¬¬,üýðÝâââ¯EÿCÏ<óLøáÏpÖÎºîÜ¹óì³Ïm±nÝº7nÌÜî©Û3CÂ¿mll?­ººúÒ¥K³þ´S§NE§®­­=þ¼ÅÏÿÌGåT³nÜ?EÌÜôÈêÒÒÜÜÚnìÜ¹3zÕÔÔ;w.ÜÇãÑc&<'>xð`fVß~ûípbxZZZ¢öÖ[oM»xãÆÑ·Â/¼ðÂ¬ÿEV_|ñÅ°Ã°õÃÓâÌíº=ó7$üÛ_ÿú×á»áÅYøî¬?-</ß±pãìÙ³UUUV>Ì|T¦üþ÷¿?xðà¬÷áO37=²ºäæèÁ099=ÃÇØË/¿<©4FgfV§Ý?ó§¥µSßÛ³þs1«©É5^åLÛ@©Û3CRwæúiûöíÃÍk¯½öÙgYölø§©¦îÙ³gÚßIyøSÄÌM¬.¹ÇUH`¸qÿþýèòôÓO÷öööõõÝ¼ysæJÓ²:íþGÉjøVIIÉ¬ÿÅÍêW_5ë·¦Ýù2kV§ý´hNOÇá¹8<k[ùlU=*£@3ÿÎC~¦þzÌºéÕ%¤¥¥åÍ7ß©¬¬L=EOLL¼úê«sÍêK/½7?ÿüóg6ºgÙ²eÑcì^H½ÛzXV]jmÜ¸1z6<cÖ××?dÍüùùÏýÛsçÎEïÎüiáþè½ÁYùl0óQùÁ¼öÚkSÿÎÌûð§Y]ZîÜ¹³~ýúêêê«W¯Fð¸Åbñxü½÷ÞkVÃO¯dW­Z@£÷¢þýßc£ÂÿÜ­[·d5K¤6PØ(áI0Ü®­­½~ýúC6ÐÌß°ÝÂÆ]³fÍ'|2ëO»|ùrøntì¨¯,ºY3Y¶qþ1sÓ#«d@x¡L&­¬¾¶¶¶åËªëÖ­ã¯UUU@V@V@V@VYYYdddÈçÎ.:VRRÒÐÐ`A@Vô%Ôe;_~ùe²Ìã©ákãããáöýû÷-È*,õ(<xpùòåÏ<óL¸gtt´¡¡¡¤¤¤¨¨hÝºu.]JýåÙ´¸¸xË-©b=AVAVÿãÅ_÷N½³ºº:úôÑÔûwìØ!« «À,Yíèè·GFF¦~+|¾fÓèËúúúðå'®_¿nTUUMý	VdøOoÞ¼9õÎÁÁÁÎÎÎd295EEE³æSVAVoâûï¿îyóÍ7£©tZV<x « «À£fµ¸¸8Ü3::zöìÙ©ßÞ>~üø§~nÄãqYY¾#«©Cb±XøóË/¿ü÷CÚÛÛedø¬^ºtiÍ5EEE7n¼qãFøîë¯¿ë>X±bEô÷îÉ*È*È*È* « « « «¬¬¬²²²²È*È*È*0ÅÿYà¸ø^Ãþ¶IEND®B`


²ºIwîÜiiiá%`%îp^Mmkk½ýÕÍ¸ÿþ~¨ëz~¶*¯Í?ýôS²0a­@étZ&¦q´YÝGýñÙªÛíYZZâ³U`¥T*¥ªêÀÀCazSSSúcHCV7y$puuõk¯½&¯Î8xîp^	ÆÇÇ«çsG²Êå íÅÎMo``@UÕöövú%«ÌjÕSq9`ÞÞ^)ëÂÂCa>===2èêêât²Ê5"ÍÕn·G"Âd«5JSùì¬U Øúúúdå7ÓÈd2ÍÍÍªª3d¬;3aíèè`(L`ff«ç¬;lppPQÙ#3eMv£555UnV²ì0·Ûò522¢ªjkk+gL¬;ottÔb±ðÎaêííÝNgg''Ò¬¥ÂãñÆ¡ìÃayIÔÓÓÃP¬%7aM$E¹Èd2­­­²Ãá6 «@)jnnÝ4W(¦z½^MÓx%²(ÙA+rþüy¢Ä'Ò8>YJßï9ÖR&¯dêóùÒé4£²4ýÈô¬x<.®²Ó;¦yÑDX; «@ÙáàÒ¥©'Od(@V2ÓÙÙ©ë:o3L&ãóù4Mg4@Vò3??¯ª*£RJ¥Ýnç _U E£QÙg³Ybéºîõzå£²ýKâí ¡¡!Ùøý~^Ü¬UAww·;¢ì#G(ùd¬Â$$¨¦Éþ¡(2ãêùÈ*Y©Äb1°òÁ^ÑÈ¤­­MÆüÜ¹sÈ*YÙd³Y]×;;;"H¥Rn·Ûn·sõ|U²ÓêïïMËÏn·		ªdUâÊh¬UlÒLX·Ûèè¨ªª~¿Ä@VÉ*Ì/+233ÃPlÁÁAij(â _U²ËåNç3N¤F£d¬¢²fT²÷O&E_¬WÏïïïg4@VÉ**×ëeÂZ(ÙlÖ8fddÑ@¥dõÖ­[²±Ùl¼wï<2==­ëºÕjÝ·oß+WÈ**Àb±1[4??ïr¹dgÂÕóQYYmjj¹¹¹H$"Á`ðôéÓ²ðá¾ùæ²pûöíkOúì³ÏÈ*L<ammme¶Â8«ç£³*³Òü²¦iòµ¶¶vyyY=zT__/5ä/²RaJ2U-»~neÆ¯ªj àêù¨Ä¬z<ÙÙYY8sæØ¡5ÿùÏ~ý¤[·n1[µ<Æ8lÂÀÀ¢(]]]ôÍêÍ7«««?._åªªªüwm6­¢b'¬|ÂºQRS7úEEg5/N»ÝnY¨««ôèñ&°,UT¦`0(Ï¦Iö	2b²[àêù¨ô¬îÝ»wzzzyyYf«GGÞzë­?þXä«<OÈ**S2T%3ÏÉdZZZ4MãiTtV-ÿü§Ãá°ÙlZZZG®]»¶gÏªª*]×É**S(r:LXÎ8Æãñpõ|0[årÖ-1N¤immåêù «dx¶p8ìp88Qd]Æ4íííÈ*YLÂ4MÅbÅ*2&H²JVÍôCÊÊyòôª4õäÉÈ*Y°ni4ZZZ¸z>È*Y°nU*r»Ýv»]öel «dØ$ÙàG8®äA0úu¹33l «dØx<^Éw8UU5rÐ/È*Y Ë9ÎP(TÿïÆÕó#ý¬U	ëôôôX,ÞÞ^6U²xÂêv»Á`üÿæ¯?44ÄÚY%«@áUÎã2Lss³¦iDõ²JVíR	w8O¥R.K¦æÈ*Y°ì§t]étÕ²JV&¬744¤ªêä9ÎY%«@1$	EQFGGMöÿÕ××'ÿ_ápSAVÉ*PT&»Ã¹üD£QÅ200ÀÊY%«@±éç27õûýªª;w5²JV&¬·°°àõz¹z>È*Y°nÕÔÔÃáðx<ô²JV×ÕÕ%ó¼2½aÜùóç~¹áÈ*YJBùÞá|ppPÚÙÙÉÕóAVÉ*PBÊñç===¢ôõõ±ú@VÉ*Àuó«çKSûûûYw «d(EG²þQ?ò µµUþ©ããã¬5U²¨²¸Ãy2ôz½.kffU²JVVâw8O$555ÍÍÍ¬,U²ÁU&¥ysãêùòoãêù «d(###¥Ô®UÔßßoi@VÉ*PfÜn÷JgÝÕÕ%¥?yò$«d¬ågllL¦;þ/Éd2>O¤ÃÃÃ¬U²«ÖÖÖ¶¶¶ý7¤ÓiÇc·Û9d¬åMäE¦­;õ¹²®ën·;J±:@VÉ*Pöü~¿×ëÝC$ç2I3WÏY%«ILMM)222Räÿîàà <+C¡'Ò¬UÀTÚÛÛ9aíìì´X,ÝÝÝ>È*YÌÆ¸ÃyqÂÍ_=``Y%«9utt8Îí°f2k6::Ê¬UÀ´Òé´<G¶õ¦óóóGâÍÕóAVÉ*`~ápØårmÓu||Ün··´´þé²JVXXXPUu;®844$OÀöövúY%«@ééé9e6-ìïT%rõ|U²T	ª¦i½½½ú%1¶Y*4UÊºõO@¥Ðmmm «@E'®ë]]][ù%ÆA¿v»½ÔîçU²Ûàà LX7Þ§Ó)Y¸2YðÃD6ñÇÆÆ$ÉHUÿ_<W%Lnèo'ÒÃaN¤È*'x½ÞP(ô?ËådvËÕó²`ccc2axæOÊó«­­M~x;.%U²gÂ*½|úÏ,,,ø|>MÓÆÇÇ1¬xÚÕb±<å$Ëe·ÛgRU²Tº|ÛUv5552£åD¬x.SSS¢;wnÕãÃÃÃªªÁÂ^@ «d0¹@ ÐØØxâÄþþþ±±1yäÈ#ÒÚîîn®UÓÕÕe±XÞxãé¨ÃáxñÅåôU²lLO%¥¡PÈív/,,477ç;ßùùÏÎÈd¬ÖÙÙ)ÓT*ey¬¦¦¦¯¯¯µµÈ*Y6ìÀçÏp8,O¢I¬Ëåbd²JVF£ÆõÓé´qOx<þÌkD «Ö!sS»Ý¿ùÄÄ®ëÆñÀÈ*w8Ç¸H!Çd¬[L&eªÊÅ²JVd¬È*Y¬@VÉ*¬UY%«U² «d@VÉ*d²JVd¬È*Y¬²Rd¬È*Y¬@VÉ*¬UY%«UÈ*YU²Y¬UY%«²ºÛí¶Z­ûöí»víÚºUY.µµµ² _ëëë×äáÃ÷´°°@Vdu5§Óy÷î]Y¯²¼î#5d:ËJÕ'LOOWWWK&åë7Ö$J]~ÒþðÍÆJÕ'455ÍÍÍ5ùå×ÏVdõ¹X­ÖUËk!«²ú>:;;+þ¹ÌS×¬Èê3X,ÿùO§Óii§ÌJå«,¯ûYU. «d²Y%«²JVd¬@VÉ*¬UY%«UÈ*YU² «d²JVd¬Èj©gõvÈ~ô#ódõïÿû/¼`ETUU)Â8TYã²ÞV:6Mfw&É*«¹¹¹»»q¨(Hä§?ý)ãPQvíÚÅ8l7²²JVAVAVAVAVAVÉ*È*È*È*YYYYYYYY%«(¦Ó§OÿéOb*ÊåË?ùäÆ¡¢üå/ùà²Y¬¢×¥Åò?ó<?il)cd;ÕÂþNøJ@Vñ¼¾øâ_~y×®]/^4ö°ÓÓÓ^¯×jµÖÕÕ=ÖØó~ôÑGûöí[5[¯ï¿ÿ~CCüðåËå»wïÊo«­­ïÚl6ËcÆèW^¯²¼î_G­_~ùå«¯¾*ë¢©©éÎ;k×~yí"×ï÷Ëokll¼qãÆº¿íÒ¥KÆå£=Ïµk×üyú¯V®´îÊú.bíªY­,@@Ú&Ç3En·ûêÕ«²N§5M33²O^^Õ>ø@(Ê³H	Æo;|øðª·÷ïßo|K¾¾þúëëþuìàlõàÁ²eAÖ¾ì×®÷üòÚ-Dþîo~óù®¼8ï®ûÛd¿,Û,rÅét2ò¥`í³2ïã?>sæÌº+÷é»µ«dµâæ+ÆaiiÉØÃær9y½õÖ[òäÉ§ÑøµY]õøÚßßkç¿%_eyÝ¿Ìj~æ*W9«VP~yí_¹kçÁùß688(wÞyçÖ­[©=ýóÏÊ|S?¾êgò¾X»êAV+îy%	GÏ_~ydd$H|ñÅkPZÕU?OVå[ÕÕÕëþuìlV>|¸î·V-¯ÝBÖÍêªßfÌSew,ûbÙk3ò¥öªÚxV/ºººÖþÌS6Çº«dµÁ÷Þoaa¡¡¡!¿L§Ó÷ïß?tèÐF³úæoÊ~ó«¯¾zõÕWGªªªçØë¯¿·9ÿ&0YÝqù´ÿ~ãMZÙcú|¾§¬ µ[Èo¼aüÝ«W¯ï®ýmò¸ñÞàéÓ§kkkùR°öYyêÔ©wÞygåÏ¬]¹OßE¬]õ «åË/¿liiill5ò¼RUUÓ´ßÿþ÷Íªü6y%[__/;Pã=%ã`ÿß±QV«UþswïÞ%«%"¿d¥ÈNP=ÏíÛ·²Ön!²Þ[[[eåîÛ·ïæÍëþ¶ééiù®ql£¯Øqë>+×²´jå>±vÕ¬¢äª×ëe¬bó"È®]»äjSSL «UÈ* «UÈ*dUÈ*dUÈ*d@Ù¸zõªqÓ±êêêÖÖV «6O×õüm;ßzë- «¶°kx,ËÉò£G¬Å3gÎìÚµëW^G[[[«««­VkSSÓ7ò?|úôiÚl¶p8¼´´ÿëy'@V²ú_G$¥+lll4~òÓO?]ùøÑ£GÉ*@V¬Õ®®.Y^XXXù-ù£|Kæ¦Æ>üñâÅ·oß§Ó¹ò70Yðß(~ñÅ+éîîöz½+iµZ×Í'YÈ*oâG$¼÷ÞÆ¬tUVÉ*@V<oVm6<²¸¸xåÊß5Þ¾páÂÜÜ,hFV²àYmhhÈ¤ªª|ýæoþ½æ¥h4JV²àY½qãÆ¾û¬VëþýûïÜ¹#ßýÕ¯~e|ëÔ©SuuuÆ	6< «Y¬@VY¬@V «¬@V «¬@V «U@V «U°Âÿ¸µ§~¶@IEND®B`


¥®ÑÝÝÍP@ÑduqqÑáp¬®®f"Ëþóå§%	çßïq^XX`( 8²êv»§¦¦~øwüÑì[xoõ ,--L¦ÎÎN#«ëínxY=@¡PÕ! h²º¶¯?Y=Õ! Ð³º®dµ`±:ßlå Ykk+«CYEn¨'Û°:UäFGG«CYEn,--V²Ü¢Äb1È*ö*LÚl6ÇÃPYE¨'ÛLLL0È*YE8Î¦¦&ÆY%«Èh4ª(«C «d¹áv»­V+«C «d90??¯×ëd¬"Á Á`XZZb(U²½J&%0È*YEÈ½ÀêÈ*YEnØív.Å¬UäÆÇÌêÈ*YEÎ477³:²¬ÎÍÍ544èõúùÒápÕr£®Á¥XÕ½fu||5«²áv»Éj¹ñù|YÝkVkjj¤£·nÝZU¹ÕrH$Coo/C¬î>«ê<UÝ¬®®®fúJVËÍ¹sçXYÝSV9"VkªnTWWÕ2L&ÍfsWWC¬îÒåË5YÂá0Y-OòF£²ºK×¯_·X,:N«ÕÊdettO3ÍÆêÈ*ç­"7"¢(ccc²JVíííõõõYÝ±Å¢n=zT£ÑÔÖÖÕ27;;+wM?C¬î4U=&d>²ÔÐÐ@VË'«C «;VñlÍæ#G<~üX²ªÕjÉjÇãáÜ¹s²º³¬JGåOõh0YJª(Êüü<C¬nWmmmæØïùóçÕ¬8q¬Bî «Õêñxdu»<xP]]-sÖ3gÎ¨·È,AeÂÊêÈ*'Ø 7YYÝ½ååå³gÏU¨&&&dÂ:22ÂP «?/WUU©áU²Ûí¶Z­r1Èê¶®·ºN§ôèYEF,ÓëõYÝî	6÷ïß7ÍòçÍ7åËÆÆF²µ:;;¹+²ºÝ¬Ê×ëë­·R©-HÆ@ ÀP «[©««S#º¸¸9ÌeÌ­¿¿_î¯X,ÆP «J§Óf³YÝîééÑétáöíÛdë¤R)Åâv»dóVápX£ÑLLL0È*YE8ÎææfÆYÝLAjjj***´Zmmmíèè(YÅfäñ'VyÌ0Èêúúú4YÞyç²Í´¶¶Úl¶T*ÅP «ëét:éèo¼ñðáCuÙBù²²²¬b3±XLQP(ÄP «gõ©ß¥Ñ>|¬b~¿ßd2±:²ºÞ»ï¾+½¤LUWWWÕcÂCCCd[ `0ÈP «?NI·À*KøYr÷ÍÏÏ3ÈêknA«ÕUl-JY­VÇÃP «·D"¬¬®?¼ö¯¤®¡¡¡®®¬b;`Õ³N§åcfffEf(VeJºÙGôz=YÅ6y<«ÕÊêÊ=«êÅVÕ	kFeeåÛo¿MV±Móóóò:l``¡PÖYÍ|$8?|Éj	Õ!ÕÿÖÖÖ=z4?X%«¥Jj2ü~?CÙjÅºÅÉ*v!)ÅeÕ>ø@]¼ðáÃ«««d»J¥l6[[[C ¬³ÊâÈ±±1yðÈòÍ*"ÓédoVY¼9Ä¥XU²åÕééiÅ¢¢®®îë¯¿&«Øµx<n0º»»åÕp8ý¥ññq²]cuåÕªª*õÕÕÕt:-òeuu5YÅ®É=k±X¼^/C ì²ªNO·¸¦YÅ.°:²­ú|¾ô²!_ÖÔÔUìQcc£ËåbWV?ÿüóì÷VGGGÉ*öÕ!cVÅW_UWW§Óé*++eczzO#'X@9fóV±OXYÝ½ñññÌ§æææN8¡Õj;víÚ5²Z¶¼^¯ÅbI&rÉêGd6Õ¥kkk'''wñKÒéôñãÇ3Y~öÙg²qýúõªª*²Z¶C0d(EV/^¼ý¥¡¡¡þ¾¾¾ìK·~ñÅv»]6úûûÿ´çþÐ¡CÜ©%¯»»[ÊÇ¥UN'-ËËË><þ¼|YYY¹£_²¸¸èp8VWW×fUæ¯[._¾,_¿ò´?þñdµ¤R)«ÕêñxåÕuËAèõúý·Û=55¥þÝì7[³ÀåC^T)233ÃP(ñ¬¾ûî»ÒÂ¿üå/2U½ÿ¾×ë/Ãáð^®¾î»]½¬¦'%UÍvºa¦©GS/sêÔ)²yàÊ50J9«[Úl¢ÝÑu_Þ¼yóØ±cò×O<yïÞ=²ï¹+rÈ*ËA oâñ¸^¯`(U²àR¬J?«9Y¬b;ÑhôûýÒÌj® «Ø¦ÁÁA.Å d³å È*¶/JÙl¶¶¶6@ÉfuËAUìH$áR¬J3«9Y¬b§¹+Ìjn «Ø¦.Å ³ºÇå È*v­££K±(µ¬rÞ*J"àR¬È*YEÎôöör)V%Õü¿¥JVL&F#bP:YÍ¼º6«ûú*YÅZCCC@édu­Ç=V²úÙgUäÝnçR¬J0«buuU²Z]]MV7ò¨ãR¬J-«ÒÔk×®ñÞ*ò¯µµK±(¬nø%ÅBVO³³³ò`àR¬>«ÙK@HSWVVÈ*òÌçóq)VEUÎ[EPWàR¬È*YEntwws)VÅÕÌ;©GÕh4µµµdBf³K±(â¬JSÕÏýÌGÈ*D(âR¬8«êÇdCf	Gyüø1«,á`555q)VEUéèòòræ¼²FåAÈ¥XeVkkk3Ç~Ï?¯fõÄd¨µµK±(Ê¬>xð ººZæ¬gÎQo±Z­|d	kaaA¯×s)VÅUN°AaêììäR¬È*YEn$	.Å (³:77×ÐÐ ×ëÕ;²BÐßßÏ¥XYVÇÇÇ3YR³*n·¬âÀ¥R)ÅÂ¥XSVkjj¤£·nÝZU¹UááaVPLYUç©êdU½9×[EáÙªÍfãR¬#«GNOO«5U7ª««É*Äää¤¢(¬ 8²zùòåìËÃa²ÂáñxL&bPY×¯_·X,:N«ÕÍæÑÑQ>	2??Ïê&«·Â'MGô¡@VÉ*öjiiÉd2±:ÍªfK|H½ëÄÄC à²Z±%.J¥l6«C(Ä¬rÅhllL&¬H¡@VÉ*rÀårY­VVP YÝì½ÕÃ<²BÅE`(bVÕ7S×55³ÝÖÖFVQh¼^¯Á``uÕ¹¹¹ÊÊÊ×^íáÃ=zóÍ7«ªª¯_¿.YYE¡ Ê#Óï÷3.«z½^]jíaaÙg¥ÓiÙØ×UìZww·¢(±X¡PXYÕétÏ@  ³ÕÈ|)ó×o¾ùf¿¯GV±k©TÊjµ¶µµ1+«o¿ývöG¾üòKuãôéÓdK±(Ä¬ÑÑÑÌRû555ï½÷z(ø¹çãÀ(dMO0+«·"¥^U¦­²JVÕ!PVïÜ¹c6³O]%«(ê¥X»»»Õµ5e©`0Èê%«jP§§§9"¥^Õëõ2>«UUUë «(:¡PÕ!DV¯^½*Yõûý="«(Rê¥XYÀÁgu³+ØUõR¬¬à³Z±>²bär¹N'ãà ³Êy«(ê¥XC¡C¬9àõz-K2d(ä5«7Pyo¥D½k0d(ä5«7Pyo%¦»»[ÊÇùË*QªÔK±z<Yr`hhH£ÑD£Q@¾³º²²b±XÔí£GÊÎ¨¶¶¬¢Ø5666773òUiªú¥@ ùÈRCCYEQSWÃ¼fUýlÍæ#G<~üXvF|d	%ÀívÛl6.Å ßY.//ËêÑ`²Ò ®qáÂ@þ²Z[[9öþüy5«'N «(F£K±¢,,,D£QÖ<)Ü¬>xð ººZæ¬gÎQo±Z­|d	¥Av=f³¹««¡@iÕn·[,¦¦&Áx£³Ê	6(mýýýò0g(Pì¯ëëëý~¿Òx<îp8|>#CVÉ*òJ½k;C¢622"Ôµ·Äb1³2a-å¬k4?þK¦§§?®ÕjívûÍ7É*P8G¦<s¯.AÙm¶¶¶©7Z,Öé,Ù¬¦Óiéh&«G½~ýºl|Y=öîÝ»ß<M~¬"?àu=z¶*aÍf6'''¿rØh4ò¨.¬Z­ÖcÇ-//ç*«===¬®UYY)vttd_*G¦³Ü©Èh4Ê¥XQÔ.^¼øÌ3ÏÈ~;HÈ2mmiiéììdd%«:NM`N.·¸¸èp8VWW³³zãÆW^yE6É$³U ya'¯&egÄP ¸È|ÔçóÉÞU"*SU³z<ÙhkkãñYUWP×TÚVÝn÷ÔÔéµ·?|øPîøÍæÄ¼·|ZXXÇ[oo/C"ÅN§¢(ýýýê$ullldddvvÁ)¬¬Ê+ÍævÚu]½ñîÝ»/¿üò½÷ø$0ßï7|ÄÅâÊ+Ájµò»"Èê7äÞRç¬jGsróLS'''O:uÿþN°AáPWàR¬(Á`P&©n·eÂ#«kïeàuG3_®]qÃÏ1UÙUÅb1+·´´¨~ù oñeå PnXlrrÒd2qà·¸³:==m±X´OÔÕÕýõ×d%L½ëÄÄCB344$ÅÖÖVüqVÕhÖ'«(ammmv»Ãk(ÒQyX*âñx8m¦¸³ZUU%õûý«««étZ6äËêêj²6;;+óò´Ùl²?dÅRÈjöGrr&+YE9ÕjeÂDFcýÌÌ£Q:³UÏ~B]Ë£¦¦¬¢´-,,èõúîînE^Õuuu©gÑpYòÒÉêçýÞêèè(YEÉò¾2È¿¥¥%Ë%ûÛ`0ÈQÊªøê«¯êêêt:]ee¥lLOOóI`É~Íh4²X9ò/Z,yøE"F£³Êy«([¡PHóóóò¦··WQÆÆFÖÑ$«d%Èét6551ÈÙéµ··«'_pY%«(MHDvsW®(°¯æççå5ìô²JVQÊZZZXûjddÄ`0X,÷"«de1PÕ!°OÔkÑ¸$$«då¢³³Sf;ÜJ$­­­ÒÔÞÞ^KVïÜ¹c63^ÝÝeÌÉ*J`÷§×ëeVÁP WÔkÑ±±1F£²ZSS³¶¦¼9YEñ¦Ó!'dÿæt:Yo¤ì²ª5oK@U,y@Z,¯×ËP`/É¤ÇãýjWWgÑcVÕ5yoøþÉê¢Äb1»£^FEFfõêÕ«ê¹É="«@cc£Ëåb°ápØh4JV%®FùfU³>²²566&O>cI¥RêY4mmm|¼Ü³Z±>²r&Fpp:¶iiiÉétrYå¼U`c±XLv¡P¡ÀÏF£f³Ùh4r¬njyyùìÙ³dåÌï÷L&N¶ÁÖ. ×ëeªÊÇÜÈêÿÄãñªª*Ö VÀõ,@ À_²ºér*NOU²îîn)+WÄD6Úív§òNYÝø#KÒû÷ïÍfùóæÍòecc#YE)Õj	CµÔ³hêëëgff²ºiVeÃëõ¾õÖ[²+á 0 V½'2¯´ü~¿<$Z[[yß¬nª®®Nèââbæ8puu5YDÓâñxss³ì²B¡o¦Õ­¤Ói³Ù¬n÷ôôèt:Ápûöm²|ÿä"$2;D"E90LF£ñÊ+Yå¼U`O<ÕjeR¶º»»eO%SÕD"ÁhU²ìÕüü¼^¯`(ÊM2lkkã,²ºcsss²ãPßdu8dXK±¡ÙÙÙúúzÙ1ÃaF¬îÀøøøºU dÃívU Õ!ÊÍÈÈÜãv»åÈê.¸uëÖÚ¬Ê4²¬500 ×ùùy¢´¥R)Ï'»Áööv.BNVwa8uC²ºººÊy«À[V(yÍÍÍ¢twwóf*YÝ¥#GHG§§§Õª·dD"òì`(Jz¼xd4Èêî³zùòåìËÃa²dcuR¥E#w.gÑÕ|øúõëE§ÓiµZ³Ù<::Ê'ÍÌÌ(2<<ÌPT*åõzånø%«·ä«Cx<îp8ôzýÇÌhU²V(R£Ñh·Û¹YÍAV5[âÀÀX¢;wNQÎÎÎd2ÉhÕeµbZ­¬ L&¿ßÏPéÝçr¹dçÃ!ä>«:Îápäçz5d¥$É%xì@m6Åbá,ä8«étúêÕ«uuu¿ò8[YY!«ÀÏJ¥R²wnkkc(ëÅz-ü"÷YÍxôèÑ¥Kjkk3ÖÊ-dØÚØØ<_äO¢ðÉÞ¦££CQsçÎñ)nìoV3îÜ¹ÓÔÔÄGís¹§q(pêY4²¬.//¿óÎ;f³93[µZ­~ú)Y~V,	P(b(z¼úápìoVWVV>ûì³µï­Úl¶ÙÙÙt:Í«Àöù|>yUÊu(JyÝÓÑÑÁµh°ïYUSZYYyêÔ©x<Î'Ý L&Ù3v¿´´´HSûûûä/«,ìÝä¼°°ÀPú'¢Ñ(£<eµbK,l<eÂÊ¥XDww·^¯©*×¢A^³ÊÀ@)2;;ËPìë©©F£	E²7§ÓÙØØÈÞü ÈkÍÆµh@VÉ*JD4	«L[üBTÎ¢Y%«()mmmmòIv#G£ÑpÈ*YE©too/Cñx¼©©IÆ9@VÉ*JS 05ÆÆÆLOp-U²%jÉ*«Cì·`0¨(Ëå	+£²JVQÊzeÏBû÷ÂE]>kÑ¬UÙ×Ûíöææf"ç2gÑÃaFd¬¢)Öý Eãp88d¬¢ì8N	G)s"sMgg'gÑ¬U#y¢*2<<ÌPìzæàà £²JVQ¾djeµZ]íz£ìøU²²¶°° ð®®.bwÔ³hÜn7ç¬Uà>K±îBæZ4E²JVÿI&K±îzÉdºrå£²JV§Ê¬··I=¦©©å@VÉ*°T*%s¯bk³h¼^/~AVÉ*°©±±1EQ8¤¹:§rÁZÕÿW/ïß¿_[[KV!³U.ÅºÅËÉ$súÙÙYFdõGétúøñã¬^»vM$k+KVQÎ&''eÂÊ¥X³©gÑ´¶¶òdõ)===¾ðÂ·oß^ÕáááWöÇ?þñÐ¡CÜ©(^¯×`0pfEss³új7SAV²¸¸èp8VWW×MO×~Ùßßÿÿ&Ï(²òH$$«~¿¡øþ§³hÌf3!YÝÛíZ×Ñì/92wîÜ9Vøþ§³hN§¼ÔàQ²ºÍÓÈ*°¡d2)ó³ööö²ÌY4]]]¬²º­¾2[¶044$ÏH$RÿïêY4¬î²£dÈ&i±Ûíåö!õ,úúz.B²Êr@.ÉTU^qÕº¢x<Î¢Y%«@î555ÍærhÌÒÒRKK<ÓC¡÷;È*YöÅÌÌ^¯¥ý¿9;;kµZëëëåÿ;d¬û¨««KÊZÂ×iQÏ¢q¹øY%«À¾[ZZ*ÕÕ!2gÑÈtå@VÉ*'RyØ'ceþÝÔÔÄEÈAVÉ*oÉdÒf³¹ù?D"T.B²JV5Mi,ÐÝÝ­EÃòI «d80£¾¾¾¨ßi·Ûíæ,U²¼±±1°é¿_vIE²JVâñx,K1;g1gÑ¬U Äãq½^ßÝÝ]DÿæT*ÕÙÙ©(gÑ¬U à×ê³h@VÉ*P F£×ë-üªz-Î¢Y%«@AS/ÅFù9d¬ÅÁáp´´´ì|º¹¹³h@VÉ*P4ÔÕ!ðËú'8d¬ÅDf6­ >[;88¨×ëÛÚÚ8d¬E&)200Pÿ©»×ëåZ4 «d(b>Ïh4.--ì?caaÁétrÈ*Yº:Ä¹sçðß%íE²JVRÐÝÝ-UK$ò_ïííUÅëõràd¬¥@zfµZ>_ÿ»KKK---E²JVR344$SÆ|Ð"ÿ-ÅÂY4 «d(MÃétæçH¬LO9d¬¥lrrR&¬ápx_ÿ+êµh8d¬¥ÏãñìëuvvÖn·sÈ*YÊº:Ä>H=F²ÊY4 «d(>Ïl6ç|usçÎ©×¢áÍTU²Édµ««+W¿Pæ¦.K£ÑÈY%«@^B!YÎÏÏïýWMNNJ¤­V«l0°YÊQ*²Ùl­­­ü=2='Ûí>ð²JVD4ÍÄÄÄîþz2*Ëoèêêâ,¬øáR¬»øÑh´¾¾Þ`0ì÷)°Y%«@ÑUedddGkxxX*YÅb!@VüÇã@nó(®üÏçÓh4EUXXXÐëõ?ûñx¼±±Qf·ýýý@VlìÂ&iëÙçÄÄÕjcÄ²`SòÄ1Í^¯w³¹¬<³X «~^¿¢(ÑhtÝíKKKn·[¾ÕÛÛËY4Y°]ãäÉ21ÄªGzgffêëëe"ËòIY°3Fæ¦@Àb±È®A¯×;ÎD"ÁàdÀÈôTRúë_ÿZæ¬ò<úÓþôÌ3Ï<ÿüó²ÍàdÀÎtvvÄb1Íò<zýõ×¬U`ÇZ[[Õ÷SÛÛÛåI4333??___ÏÈd¬;ÖÕÕe#H¨×¢	B---@VÉ*°c275LÅ£Ñ¨Ùlfå¬U`&&&,Ýnoll4ÛYÎY°X,&SUÑÈ*YU² «d²Y%«²JVd¬@VÉ*¬UY%«UÈ*YU² «d²Ê «d@VÉ*d²JVd¬È*Y¬@V6>>®Ñüø/¹yó¦ÙlÖjµÇ»zõ*YÕH§ÓÇÏdµ­­íÒ¥K²ñÎ;ï9sF6?~¼ü´D"AVdu===¬VUU­®®ÊÆÊÊJmm­lttth²Èt;@V²¸¸èp8¤£¬J/3ßU·ççç¿|Ú'|¢Óé¸Sdõ)n·jjêÇOY­¨¨È|WÚÉ«²º]ëíÊ-ÕÕÕ+++êA`Ù&«²º¾ª/½ôÒ| òg[[YÕttÝ×®]«©©©¨¨0ÍÓÓÓd@VY@VÉ*d²JVd¬È*Y¬UY%«²JV «U² «d@VÉ*d¬È*YÕ"Èê·ßüæ7¥Õýë_Ò"G***EaÊÜãr¿3ÜéØ5ÝE£ÑÉ*rËétÆ¡¬ø|¾ßýîwCY>|ø0ã°ßÈ*È*YYYYYY%« « « «dddddddd¬".]ºô·¿ýq(+_~ùåGÄ8üã/^dÈ*d²"¾/5mþÌv~Råv»ØBÆU|Vsû;Qàw:²íúöÛoöÙÃñÅêöæÍC«ÕVWW_¾|YÝó¾÷ÞÇ[7[?ÿú×¿ÖÕÕÉùårËÝ»wå·UUUÉÏËwu:æ	õ?ôÜsÏÉOÊ²½á_G­½îÝ»wòäI¹/N8qçÎìû=³ý¿ër¹ä·544Ü¸qcÃß6>>®.m·Û¯]»ÆàÈÓ?ûY¹ÖwîÖ»ì»dµ¼¸Ýnilôôô¨Ï"Í655%ñxÜ`0¨ÏÙ'®®®fgõâÅr»DQErK[[úÛÞzë­uO>­~Kþ|á6üë8ÀÙê/¾(÷²lÈ½/»Åìû=³ý¿ûÆoÈwåÅ|wÃß&ûeyÉÆÕ«W­V+#_²|ðÁ~¸á»õ."û®Y-»ùúdH§Óê6JÉsì¥^'O&êÏdguÝíÙ¿-³×Î|Kþíÿ:0««P_å¬»2ÛÙÌ=Îü¶¡¡!Ü¼úê«ß|óÃ^hOÿÌ³2ÓÔ¾¾¾u?±õ."û®Y-»ç$P6VVVÔgÈ³Ï>'''¿ýöÛì(­ËêºÛ·UùVeeåÕÇoø­uÛÙ³ºî·©óTÙË¾XöÚ|¡½ªVê ¿ßý3[<Ö><6¼ëAVËH[[Ûo¾H$êêê2»Èx<¾¼¼üÊ+¯ì4«gÎýæwßwòäIõõ9öÂ/d6gÕ¹N>­¤=fccãwPö#äøúw§¦¦ÔãÙ¿MnW^ºt©ªª/ÙÏÊ÷ßÿÕW_]û3ÙwîÖ»ì»dµ¼Ü»w¯©©©¡¡annNnÈóJ¯×wßw§Yß&¯dkkkeªSR?ìðß>¥Õjå?w÷î]²Z 2wÜ)²m»Ý~ûöí-î ìGÜïÍÍÍrç;vì«¯¾Úð·Ý¼yS¾«^[í+ÜÏÊì,­»s·ÞEdßõ «Èy¡êp8 «Ø=Ïwøðay¡zâÄ	þ2 @V «U@V «UÈ* «UÈ* «UÈ*¢155¥^t¬²²²¹¹È*Ý3ÍËv¾ôÒK@Vìa×ðD*í «@¹GñÃ?<|øðsÏ='·,..677WVVjµÚ'NÜ¸q#óÃ.]¹©N§óz½ét:ó×3O¬dõG/¾ø¢Ü")]cCCú~úéÚÛÏ?OV²`¬úý~ÙN$k¿%_Ê·dnª~ÙØØ(_~ñÅ·oß«Õºö70Yðc¿ýöÛµ7ÎÎÎÃ±6Z­vÃ|U¬Ø4ï½÷Üòæoª³ÒuY]]]%«Y°Ý¬êt:¹eqqñêÕ«k¿«½uël²U?ÕºººÌGôz½üùðáÃÿfd©««¬dÀÏdõÆÇÓjµ§O¾sç|÷µ×^S¿õþûïWWW«'Ø<zô¬d²Yd²Y¬²Y¬²Y¬@VY¬@VÀÿ+ê½^ü]Ô@IEND®B`


³Ù;ñ*§16ÓÖÖ&åg)6>²ú¥ørs`3rû´Z­uuu,Å:RSi*çüYýï1Sx²ÒÕÕ%÷ÞÞ^Qdu«cóÚ*°Mµµµ¼1QduS7nÜP'ûüóÏÉ*ðX###Z­¶½½¥`TYÝôIàuxË°ææfNr®LMM1ª¬>"o#¼e	ØÂÜÜKÑÙÙÉ¨²ÊçV½ºtéF£c?¼0Lª «±´´ôòË/U`kµµµ555¹üÞ%¿ßÏ¨²º^$),,äpÀNæòqdT5Íª «[BÑëõ©zW0YEfñx<9 `y<!*Âá07ÕõoY.,,ÈOùsttT¾t:dx¬©©)ÁÐÜÜkÿãrW=<ªà6²ºqVeÃëõ>:ò$0°2´åÚmººº8<2ÈêÆJKKUD§§§cÏU`;ä¨Ýnw¹5ªZ­VFUÕ­®®ÍfµöìY½^o4ïÝ»GVmêïï£===9òÿéÒ%FUU>·ì£úúú9P°ü?Êÿ)£*È*Yö:PpgggÖÿFUÕÇ¨¨¨0êEVÃAVjnn6H$»GU»ÝÎ¨²º¾¾¾uGËEVÉ÷ôô0ª¬nëpwïÞÏªL®dØ©@ Ýv8ª «Û:1Ú¬®­­ñ¹U`wÔs¤555Ù:ªÊÎall+du+»ÊÐÐª©Úàs«Àî¨ÛtuueßÿÓé¬­­å*YîîîÄÓÁýñáÃu:<TUoª®®KªªªnÝº%,//<yR¯×B!²låñxl6ÛÜÜýO]¿~]öò'×/ÈêãX­V	TÐl6_½zu§¿¡¬¬L~tEElHM¯¢~yaa¡l>úµ×^[[[¦ÊNG.ù÷¿ÿ½ü¨²L§üÊ+¯dÓÿÔÑ£GUAVS#???þËk×®É«B÷îÝøo8q"qP´s5#ÓuvvJY³æì.~ø!£*Èjj766ÆXPP ÷FaåKéå/ùK®ª·oßKîÜ¹óÿ%?)C3W32:Qvüï?~Qdu[oÞÌîÞ	¼¸¸èv»Ö.V½J~ç¥Kdãã?Þì¼¶¬ÕÛ2ý$1ª¬n7«yÉr§¿pvvöäÉóóóßR¿-þÝÅý~²lRWWWYYév¹ª «ÛÍª^¯Áqïç«Ç³Ç[XXÓÄÄz°|K6ßÿõ&áÍNNVM&''ßïÏèQU«Õ2ª¬në|p7nÜ(--=ñ[QQ!w¡æYþ´X,ñÏ!Ë%£££UUU29rD°ö<ìK¶9YEéèè0L `·ÛÍ¨²º3þù»ï¾ßEi­á ½´ÍfËÐË¾Qdu÷îß¿/K÷ò%²$Rï]ÊÄË¨­bö1«KKK.Í±iUîw¿#«@²8*UA6põ¬nËÊÊÊ+Wâ_[­¬¬[]]å4æ@r©gSzz2èßìõzåv¦¿8¸¬ªæçç;v,¤ÉÑ$È*²USSÙlÉíøø8£*ÈjêAVÍHP[ZZ2åA£*ÈêÎämi «ÀÖdøPÁ4ÿwÃaÁÀ¨²È*²n·;ÍÿêÄvª «dHwéÖR^UY%«@&i5Ì¨²JVLÎV£êÅ¹@VÉ*1ÚÚÚFc~ØFFÕôüd¬Û¹Õjõz½iõ¯Ê½OÏ²JVÓÕÕ¥ÕjGFF¡²JV$¨««KÙKM¥©ª «dÈTiõQ	ªÉdbTY%«@óù|ÅÅÅ)U;::¸F@VÉ*ÁÔ%®)Uå177Ç5²JVÌò«Qµ½½ëd¬ÙÀétÖÕÕ¥pT;]8æY%«@6B2°ööö¦jTmjjâZY%«@öHÕxU²d¡H$rð/pÊÝQ «@v¦Jä¤¯ö_ªY²S4µÙlç ÿsªY²Voo¯F£éïïgTÈ*Yà`¬FÕt;@VÉ*ds `ùý2Ë®È*åöû@ÁjT­¯¯g©²d¿ý>P0£*@VÜ²'9gTÈ*jÚQuU²¤/)ôOÆÖ¤ªöÑX¬U x½^«ÕÄÓ ªçSx:¬U eÔéeÚÚÚõkkkÝn7U Gùý~¹GLNNîýW]¿~]£ÑÈ¬*@VF+++2bîÓ ²JVL)Q «¾"ÓªÌ¬9É9£*@V|%¿ß¿»¿.»UÁ +	U_jkk3SSS»ø»õõõc/Ã.@VÉ*U$ååå»8:%Noo/kUÿ5M(ÚÑßr¹ªY°ÚÚZ»Ý¾ýFÊÎBFÕ «ÖSÏè^¼xq?ïñxÊËËU²`c­­­Û<É¹jprÖU²d	ªÉdjnn~ìO655É¨*÷) «6uþüùÇ&È]éÒ¥K,@VÉ*ðN§³¶¶vMeTµZ­¼ªU²<^(õòåË~W©³³È*Y¶Eä|Ã÷.577Íf^UÈ*Y¶kjjJFÒÄËå&éüùó,@VÉ*°ÒN)k8¿PBË¨U²ìFeeåñãÇc_ÎÍÍFFU¬U`7Ôe§ ¾ìèè0L»;Ñ@VÓ(«CCCÖétv»ttT.¨®®KªªªnÝºûÉ¾¾>ÙU Y~ô£Íf§Ó)÷Á'xâ^`MÏjYYÙÀÀltwwWTTÈÔôÊ+²!ª[]]~,«<øË£þð?U`û¢Ñè·¿ýí¼¼¼S§N½ôÒKz½¾´´4°2@fg5^~~~ü×®]Vm=öÜ¹s±¬8qB@f`:;;ëêê~þóF­VÛÞÞÞÜÜ¼Ó²d5M³:<<ÜØØM¤2ÂÊÓÓÓcmm-Õ¿ÿýïGýéObZ¶ÏívÁO>ùD=*;+dCVåN¾´´a___QQl¸7o~ùoåµU yYU§S½|ù²Ïç²dCVgggO<9??ø-N§j¬wñâE§Ó©¶Õ½^oKK+dvVC¡Ð±cÇâßÄ411¡Þ$,ßzäßÊ´$I4=~ü¸UfÖ?ü°¡¡AFU>`dpVU#-ËºIttt´ªªJæÔ#G¬aÉ*@@*mmm¥©@6L«@VÉ*¬UÈ*YU² «d²ÊÕ «d@VÉ*¬UÈ*YU² «d²Y%«²JVd¬@VÉ*¬UY%«UÈ*YU² «d²JVd¬È*Y¬@VÉ*¬UY%«U² «d@VÉ*d²JVd¬È*Y¬UY%«²JV «U² «d@VÉ*d¬È*YU² «d²JVd¬È*Y¬UY%«²JVd5í²:44tøðaNg·ÛGGGåêêj¹¤ªªêÖ­[þYÕÈFwwwEElHM¯"ryaaá?óç?ÿùÿ>ê7¿ù^¯çjätVãåççÇyíÚ5O7ü'Nü¯2Îr5ÈêÕöêêjAAF£ñt³áI`YÝØââ¢Ûí^ZZ¿°¯¯¯¨¨hë!«²úÙÙÙ'OÎÏÏ'~K§Ó=ögÈ*¬~%;vlaa!þMLêÀò­¬Èê£ÿaÍÿiÅ¢#VUUÉzäÈ5&þÌfYÀøîw¿ÍøôÓOxâ	áþç´Z-ë#äºÎËËcrêÎ"$ÌrYU$ÜëÁ ë#ð°9â/ùF£ùë_ÿÊRìãs±,È*YeÈ*È*È*È*È*YYYY%« « « « « « « «d©óúë¯üñÇ¬Cøè£ÞÿÖ!G|úé§¿øÅ/þñ°d²YE6Í6f;?©¸6=qÕdéÕäþN¤íÕ¬bg<xðäOv-vêÃ¡ÓéÔiáåò·Þz«ªªjÝ´*¾öÚk¥¥¥òÃôÑÏÇ'¿­°°P~^¾«×ëcg;ÿÐSO=%?)Êö&þª?räÕÕÕ÷ïßO¼ÆcÛ·ù»uuuòÛ***7ümòe^^Ýn¿uëò»|â=qÝ¹I¯Ö­wW:Èjîr¹6Ù8ö¬ºGUVVÞ¼yS6"ÑhT÷Ù3®­­%fõ7ÞË%êL·n·[ý¶Ó§O¯ºøgQß?~úéÿ:R2­>ûì³rýÊ²sL¼ÆcÛ·ù»§NïÊÃ2ùî¿MöÎrë7nØl6V>µï1ï¼óÎï½·áÕºõn!ñJYÍé©EÝ1VWWÕ~6Êýíùç;R,êg³ºîòÄßÛwÇ¾%Êö)ÉjlrêñÍº«&¶xÛ]­spì·]ºtIF^xOa¥Õ]>vO5õÜ¹së~&fëÝBâ²Ó÷1I l¬¬¬¨ËO>C¡Ðß ´.«ë.ßNVå[ùùùþu¤*«ËËË~kÝvâmcÃ¬®ûmjN²ìeßÍÊ§Ï#iuOT|>_âÏlqÕÇß06¼ÒAVsÛí~õÕW§¦¦JKKc;ÊH$²´´ÔØØ¸Ó¬>÷Üs²÷üäO9¢.ÉËËS÷·§~:ölsìI`²B±«ægQOÒÊ~ÓétnqÕ$Þ6~øÃª¿óæMõlaâoËÕ3ï¾ûnaa!+Z÷Ä·ß~û^ÿÄ«uëÝBâ²»æççkkk+**&&&ÔýDîcÁh4¾ùæ;Íªü6yTk±Xd7ª_Ro|øâ?ïÒétò%«)»jäê]¡lÛíö÷îmqÕ$Þ6ä?zô¨UUU·oßÞð·ÊwÕÏU_BÞß²´îjÝz·x¥¬"ÉäA«Ãá`¬b÷äAkuuµ¿,UÈ*d²È*d²È*d²Yd² Ü¼ySz,??ÿèÑ£,@VìÙl¼óùçgA²`û¢Ñ¨l¯¬¬° Yr=ï½÷^AAÁSO=%LOO=z4??_§ÓUWWÇ~øÝwßÙT¯×½ÞÕÕÕØ_a=²Õ¯<ûì³r¤4þÂõ¿ûÝïâ/?sæYÈ*²êóùdjj*þ[ò¥|KfSõ¥Óé/¯]»vïÞ=Ù°Ùlñ¿È*¯¢øàÁøÇÆÆZ[[G|2u:Ýù$«Y°ißzë-¹äÕW_USéº¬®­­U¬ØnVõz½==ãÆøïª'¯^½z÷î]Ù0d «ÕÒÒÒØ[ü¹¸¸øEÂ[ZZZÈ*@V<&«ÃÃÃUUU:îg¹ÿ¾|÷g?ûúÖÛo¿]TT¤>`óùçU¬@V «¬@V «U@V «U@V «UÈ* «UÈ*øâÿc3¹ÁIEND®B`


CORRELATIONS
  /VARIABLES=meangaitspeed steplength stridelength supportbase steptime swingtime stancetime singlesupportbase doublesupportbase
  /PRINT=TWOTAIL NOSIG
  /STATISTICS DESCRIPTIVES
  /MISSING=PAIRWISE.


Correlations


Notes	
Output Created	21-DEC-2015 17:29:14	
Comments		
Input	Active Dataset	DataSet1	
	Filter	<none>	
	Weight	<none>	
	Split File	<none>	
	N of Rows in Working Data File	100	
Missing Value Handling	Definition of Missing	User-defined missing values are treated as missing.	
	Cases Used	Statistics for each pair of variables are based on all the cases with valid data for that pair.	
Syntax	CORRELATIONS
  /VARIABLES=meangaitspeed steplength stridelength supportbase steptime swingtime stancetime singlesupportbase doublesupportbase
  /PRINT=TWOTAIL NOSIG
  /STATISTICS DESCRIPTIVES
  /MISSING=PAIRWISE.	
Resources	Processor Time	00:00:00.00	
	Elapsed Time	00:00:00.02	


Descriptive Statistics	
	Mean	Std. Deviation	N	
meangaitspeed	.6303	.18869	100	
steplength	.4123	.07731	100	
stridelength	.8291	.15214	100	
supportbase	.1551	.05842	100	
steptime	.6573	.12376	100	
swingtime	.4180	.07295	100	
stancetime	.9098	.19682	100	
singlesupportbase	.4168	.07045	100	
doublesupportbase	.2398	.07014	100	


Correlations	
	meangaitspeed	steplength	stridelength	
meangaitspeed	Pearson Correlation	1	.728**	.744**	
	Sig. (2-tailed)		.000	.000	
	N	100	100	100	
steplength	Pearson Correlation	.728**	1	.975**	
	Sig. (2-tailed)	.000		.000	
	N	100	100	100	
stridelength	Pearson Correlation	.744**	.975**	1	
	Sig. (2-tailed)	.000	.000		
	N	100	100	100	
supportbase	Pearson Correlation	-.196	-.301**	-.300**	
	Sig. (2-tailed)	.051	.002	.002	
	N	100	100	100	
steptime	Pearson Correlation	-.607**	-.059	-.054	
	Sig. (2-tailed)	.000	.558	.595	
	N	100	100	100	
swingtime	Pearson Correlation	-.343**	.171	.187	
	Sig. (2-tailed)	.000	.089	.062	
	N	100	100	100	
stancetime	Pearson Correlation	-.685**	-.192	-.191	
	Sig. (2-tailed)	.000	.055	.057	
	N	100	100	100	
singlesupportbase	Pearson Correlation	-.340**	.144	.160	
	Sig. (2-tailed)	.001	.154	.112	
	N	100	100	100	
doublesupportbase	Pearson Correlation	-.699**	-.384**	-.395**	
	Sig. (2-tailed)	.000	.000	.000	
	N	100	100	100	

Correlations	
	supportbase	steptime	swingtime	stancetime	
meangaitspeed	Pearson Correlation	-.196	-.607**	-.343**	-.685**	
	Sig. (2-tailed)	.051	.000	.000	.000	
	N	100	100	100	100	
steplength	Pearson Correlation	-.301**	-.059	.171	-.192	
	Sig. (2-tailed)	.002	.558	.089	.055	
	N	100	100	100	100	
stridelength	Pearson Correlation	-.300**	-.054	.187	-.191	
	Sig. (2-tailed)	.002	.595	.062	.057	
	N	100	100	100	100	
supportbase	Pearson Correlation	1	-.236*	-.433**	-.177	
	Sig. (2-tailed)		.018	.000	.078	
	N	100	100	100	100	
steptime	Pearson Correlation	-.236*	1	.829**	.958**	
	Sig. (2-tailed)	.018		.000	.000	
	N	100	100	100	100	
swingtime	Pearson Correlation	-.433**	.829**	1	.769**	
	Sig. (2-tailed)	.000	.000		.000	
	N	100	100	100	100	
stancetime	Pearson Correlation	-.177	.958**	.769**	1	
	Sig. (2-tailed)	.078	.000	.000		
	N	100	100	100	100	
singlesupportbase	Pearson Correlation	-.385**	.777**	.949**	.718**	
	Sig. (2-tailed)	.000	.000	.000	.000	
	N	100	100	100	100	
doublesupportbase	Pearson Correlation	.108	.701**	.345**	.787**	
	Sig. (2-tailed)	.283	.000	.000	.000	
	N	100	100	100	100	

Correlations	
	singlesupportbase	doublesupportbase	
meangaitspeed	Pearson Correlation	-.340**	-.699**	
	Sig. (2-tailed)	.001	.000	
	N	100	100	
steplength	Pearson Correlation	.144	-.384**	
	Sig. (2-tailed)	.154	.000	
	N	100	100	
stridelength	Pearson Correlation	.160	-.395**	
	Sig. (2-tailed)	.112	.000	
	N	100	100	
supportbase	Pearson Correlation	-.385**	.108	
	Sig. (2-tailed)	.000	.283	
	N	100	100	
steptime	Pearson Correlation	.777**	.701**	
	Sig. (2-tailed)	.000	.000	
	N	100	100	
swingtime	Pearson Correlation	.949**	.345**	
	Sig. (2-tailed)	.000	.000	
	N	100	100	
stancetime	Pearson Correlation	.718**	.787**	
	Sig. (2-tailed)	.000	.000	
	N	100	100	
singlesupportbase	Pearson Correlation	1	.366**	
	Sig. (2-tailed)		.000	
	N	100	100	
doublesupportbase	Pearson Correlation	.366**	1	
	Sig. (2-tailed)	.000		
	N	100	100	

**. Correlation is significant at the 0.01 level (2-tailed).	
*. Correlation is significant at the 0.05 level (2-tailed).	

REGRESSION
  /DESCRIPTIVES MEAN STDDEV CORR SIG N
  /MISSING LISTWISE
  /STATISTICS COEFF OUTS CI(95) R ANOVA
  /CRITERIA=PIN(.05) POUT(.10)
  /NOORIGIN
  /DEPENDENT meangaitspeed
  /METHOD=ENTER steplength stridelength supportbase steptime swingtime stancetime singlesupportbase doublesupportbase.


Regression


Notes	
Output Created	21-DEC-2015 17:46:45	
Comments		
Input	Active Dataset	DataSet1	
	Filter	<none>	
	Weight	<none>	
	Split File	<none>	
	N of Rows in Working Data File	100	
Missing Value Handling	Definition of Missing	User-defined missing values are treated as missing.	
	Cases Used	Statistics are based on cases with no missing values for any variable used.	
Syntax	REGRESSION
  /DESCRIPTIVES MEAN STDDEV CORR SIG N
  /MISSING LISTWISE
  /STATISTICS COEFF OUTS CI(95) R ANOVA
  /CRITERIA=PIN(.05) POUT(.10)
  /NOORIGIN
  /DEPENDENT meangaitspeed
  /METHOD=ENTER steplength stridelength supportbase steptime swingtime stancetime singlesupportbase doublesupportbase.	
Resources	Processor Time	00:00:00.02	
	Elapsed Time	00:00:00.02	
	Memory Required	4004 bytes	
	Additional Memory Required for Residual Plots	0 bytes	


Descriptive Statistics	
	Mean	Std. Deviation	N	
meangaitspeed	.6303	.18869	100	
steplength	.4123	.07731	100	
stridelength	.8291	.15214	100	
supportbase	.1551	.05842	100	
steptime	.6573	.12376	100	
swingtime	.4180	.07295	100	
stancetime	.9098	.19682	100	
singlesupportbase	.4168	.07045	100	
doublesupportbase	.2398	.07014	100	


Correlations	
	meangaitspeed	steplength	stridelength	
Pearson Correlation	meangaitspeed	1.000	.728	.744	
	steplength	.728	1.000	.975	
	stridelength	.744	.975	1.000	
	supportbase	-.196	-.301	-.300	
	steptime	-.607	-.059	-.054	
	swingtime	-.343	.171	.187	
	stancetime	-.685	-.192	-.191	
	singlesupportbase	-.340	.144	.160	
	doublesupportbase	-.699	-.384	-.395	
Sig. (1-tailed)	meangaitspeed	.	.000	.000	
	steplength	.000	.	.000	
	stridelength	.000	.000	.	
	supportbase	.025	.001	.001	
	steptime	.000	.279	.298	
	swingtime	.000	.044	.031	
	stancetime	.000	.028	.028	
	singlesupportbase	.000	.077	.056	
	doublesupportbase	.000	.000	.000	
N	meangaitspeed	100	100	100	
	steplength	100	100	100	
	stridelength	100	100	100	
	supportbase	100	100	100	
	steptime	100	100	100	
	swingtime	100	100	100	
	stancetime	100	100	100	
	singlesupportbase	100	100	100	
	doublesupportbase	100	100	100	

Correlations	
	supportbase	steptime	swingtime	stancetime	
Pearson Correlation	meangaitspeed	-.196	-.607	-.343	-.685	
	steplength	-.301	-.059	.171	-.192	
	stridelength	-.300	-.054	.187	-.191	
	supportbase	1.000	-.236	-.433	-.177	
	steptime	-.236	1.000	.829	.958	
	swingtime	-.433	.829	1.000	.769	
	stancetime	-.177	.958	.769	1.000	
	singlesupportbase	-.385	.777	.949	.718	
	doublesupportbase	.108	.701	.345	.787	
Sig. (1-tailed)	meangaitspeed	.025	.000	.000	.000	
	steplength	.001	.279	.044	.028	
	stridelength	.001	.298	.031	.028	
	supportbase	.	.009	.000	.039	
	steptime	.009	.	.000	.000	
	swingtime	.000	.000	.	.000	
	stancetime	.039	.000	.000	.	
	singlesupportbase	.000	.000	.000	.000	
	doublesupportbase	.141	.000	.000	.000	
N	meangaitspeed	100	100	100	100	
	steplength	100	100	100	100	
	stridelength	100	100	100	100	
	supportbase	100	100	100	100	
	steptime	100	100	100	100	
	swingtime	100	100	100	100	
	stancetime	100	100	100	100	
	singlesupportbase	100	100	100	100	
	doublesupportbase	100	100	100	100	

Correlations	
	singlesupportbase	doublesupportbase	
Pearson Correlation	meangaitspeed	-.340	-.699	
	steplength	.144	-.384	
	stridelength	.160	-.395	
	supportbase	-.385	.108	
	steptime	.777	.701	
	swingtime	.949	.345	
	stancetime	.718	.787	
	singlesupportbase	1.000	.366	
	doublesupportbase	.366	1.000	
Sig. (1-tailed)	meangaitspeed	.000	.000	
	steplength	.077	.000	
	stridelength	.056	.000	
	supportbase	.000	.141	
	steptime	.000	.000	
	swingtime	.000	.000	
	stancetime	.000	.000	
	singlesupportbase	.	.000	
	doublesupportbase	.000	.	
N	meangaitspeed	100	100	
	steplength	100	100	
	stridelength	100	100	
	supportbase	100	100	
	steptime	100	100	
	swingtime	100	100	
	stancetime	100	100	
	singlesupportbase	100	100	
	doublesupportbase	100	100	


Variables Entered/Removeda	
Model	Variables Entered	Variables Removed	Method	
1	doublesupportbase, supportbase, steplength, singlesupportbase, steptime, swingtime, stridelength, stancetimeb	.	Enter	

a. Dependent Variable: meangaitspeed	
b. All requested variables entered.	


Model Summary	
Model	R	R Square	Adjusted R Square	Std. Error of the Estimate	
1	.947a	.896	.887	.06347	

a. Predictors: (Constant), doublesupportbase, supportbase, steplength, singlesupportbase, steptime, swingtime, stridelength, stancetime	


ANOVAa	
Model	Sum of Squares	df	Mean Square	F	Sig.	
1	Regression	3.158	8	.395	97.996	.000b	
	Residual	.367	91	.004			
	Total	3.525	99				

a. Dependent Variable: meangaitspeed	
b. Predictors: (Constant), doublesupportbase, supportbase, steplength, singlesupportbase, steptime, swingtime, stridelength, stancetime	


Coefficientsa	
Model	Unstandardized Coefficients	Standardized Coefficients	t	Sig.	
	B	Std. Error	Beta			
1	(Constant)	.652	.068		9.636	.000	
	steplength	-.175	.370	-.072	-.473	.637	
	stridelength	.934	.191	.753	4.880	.000	
	supportbase	-.512	.129	-.159	-3.982	.000	
	steptime	-.698	.217	-.458	-3.220	.002	
	swingtime	-.335	.409	-.129	-.819	.415	
	stancetime	-.002	.157	-.002	-.011	.992	
	singlesupportbase	-.047	.311	-.018	-.153	.879	
	doublesupportbase	-.105	.213	-.039	-.495	.622	

Coefficientsa	
Model	95.0% Confidence Interval for B	
	Lower Bound	Upper Bound	
1	(Constant)	.518	.787	
	steplength	-.910	.560	
	stridelength	.554	1.314	
	supportbase	-.768	-.257	
	steptime	-1.128	-.267	
	swingtime	-1.146	.477	
	stancetime	-.313	.309	
	singlesupportbase	-.664	.570	
	doublesupportbase	-.528	.317	

a. Dependent Variable: meangaitspeed	

REGRESSION
  /DESCRIPTIVES MEAN STDDEV CORR SIG N
  /MISSING LISTWISE
  /STATISTICS COEFF OUTS CI(95) R ANOVA
  /CRITERIA=PIN(.05) POUT(.10)
  /NOORIGIN
  /DEPENDENT meangaitspeed
  /METHOD=ENTER stridelength supportbase steptime.


Regression


Notes	
Output Created	21-DEC-2015 18:16:22	
Comments		
Input	Active Dataset	DataSet1	
	Filter	<none>	
	Weight	<none>	
	Split File	<none>	
	N of Rows in Working Data File	100	
Missing Value Handling	Definition of Missing	User-defined missing values are treated as missing.	
	Cases Used	Statistics are based on cases with no missing values for any variable used.	
Syntax	REGRESSION
  /DESCRIPTIVES MEAN STDDEV CORR SIG N
  /MISSING LISTWISE
  /STATISTICS COEFF OUTS CI(95) R ANOVA
  /CRITERIA=PIN(.05) POUT(.10)
  /NOORIGIN
  /DEPENDENT meangaitspeed
  /METHOD=ENTER stridelength supportbase steptime.	
Resources	Processor Time	00:00:00.00	
	Elapsed Time	00:00:00.00	
	Memory Required	2068 bytes	
	Additional Memory Required for Residual Plots	0 bytes	


Descriptive Statistics	
	Mean	Std. Deviation	N	
meangaitspeed	.6303	.18869	100	
stridelength	.8291	.15214	100	
supportbase	.1551	.05842	100	
steptime	.6573	.12376	100	


Correlations	
	meangaitspeed	stridelength	supportbase	steptime	
Pearson Correlation	meangaitspeed	1.000	.744	-.196	-.607	
	stridelength	.744	1.000	-.300	-.054	
	supportbase	-.196	-.300	1.000	-.236	
	steptime	-.607	-.054	-.236	1.000	
Sig. (1-tailed)	meangaitspeed	.	.000	.025	.000	
	stridelength	.000	.	.001	.298	
	supportbase	.025	.001	.	.009	
	steptime	.000	.298	.009	.	
N	meangaitspeed	100	100	100	100	
	stridelength	100	100	100	100	
	supportbase	100	100	100	100	
	steptime	100	100	100	100	


Variables Entered/Removeda	
Model	Variables Entered	Variables Removed	Method	
1	steptime, stridelength, supportbaseb	.	Enter	

a. Dependent Variable: meangaitspeed	
b. All requested variables entered.	


Model Summary	
Model	R	R Square	Adjusted R Square	Std. Error of the Estimate	
1	.944a	.892	.888	.06306	

a. Predictors: (Constant), steptime, stridelength, supportbase	


ANOVAa	
Model	Sum of Squares	df	Mean Square	F	Sig.	
1	Regression	3.143	3	1.048	263.485	.000b	
	Residual	.382	96	.004			
	Total	3.525	99				

a. Dependent Variable: meangaitspeed	
b. Predictors: (Constant), steptime, stridelength, supportbase	


Coefficientsa	
Model	Unstandardized Coefficients	Standardized Coefficients	t	Sig.	
	B	Std. Error	Beta			
1	(Constant)	.613	.064		9.650	.000	
	stridelength	.832	.044	.671	18.877	.000	
	supportbase	-.443	.118	-.137	-3.756	.000	
	steptime	-.919	.053	-.603	-17.285	.000	

Coefficientsa	
Model	95.0% Confidence Interval for B	
	Lower Bound	Upper Bound	
1	(Constant)	.487	.740	
	stridelength	.744	.919	
	supportbase	-.677	-.209	
	steptime	-1.025	-.814	

a. Dependent Variable: meangaitspeed	

REGRESSION
  /DESCRIPTIVES MEAN STDDEV CORR SIG N
  /MISSING LISTWISE
  /STATISTICS COEFF OUTS CI(95) R ANOVA COLLIN TOL ZPP
  /CRITERIA=PIN(.05) POUT(.10)
  /NOORIGIN
  /DEPENDENT meangaitspeed
  /METHOD=ENTER stridelength supportbase steptime
  /SCATTERPLOT=(*ZRESID ,*ZPRED)
  /RESIDUALS NORMPROB(ZRESID)
  /CASEWISE PLOT(ZRESID) OUTLIERS(3)
  /SAVE MAHAL COOK.


Regression


Notes	
Output Created	21-DEC-2015 18:43:41	
Comments		
Input	Active Dataset	DataSet1	
	Filter	<none>	
	Weight	<none>	
	Split File	<none>	
	N of Rows in Working Data File	100	
Missing Value Handling	Definition of Missing	User-defined missing values are treated as missing.	
	Cases Used	Statistics are based on cases with no missing values for any variable used.	
Syntax	REGRESSION
  /DESCRIPTIVES MEAN STDDEV CORR SIG N
  /MISSING LISTWISE
  /STATISTICS COEFF OUTS CI(95) R ANOVA COLLIN TOL ZPP
  /CRITERIA=PIN(.05) POUT(.10)
  /NOORIGIN
  /DEPENDENT meangaitspeed
  /METHOD=ENTER stridelength supportbase steptime
  /SCATTERPLOT=(*ZRESID ,*ZPRED)
  /RESIDUALS NORMPROB(ZRESID)
  /CASEWISE PLOT(ZRESID) OUTLIERS(3)
  /SAVE MAHAL COOK.	
Resources	Processor Time	00:00:00.28	
	Elapsed Time	00:00:00.42	
	Memory Required	2076 bytes	
	Additional Memory Required for Residual Plots	552 bytes	
Variables Created or Modified	MAH_1	Mahalanobis Distance	
	COO_1	Cook's Distance	


Descriptive Statistics	
	Mean	Std. Deviation	N	
meangaitspeed	.6303	.18869	100	
stridelength	.8291	.15214	100	
supportbase	.1551	.05842	100	
steptime	.6573	.12376	100	


Correlations	
	meangaitspeed	stridelength	supportbase	steptime	
Pearson Correlation	meangaitspeed	1.000	.744	-.196	-.607	
	stridelength	.744	1.000	-.300	-.054	
	supportbase	-.196	-.300	1.000	-.236	
	steptime	-.607	-.054	-.236	1.000	
Sig. (1-tailed)	meangaitspeed	.	.000	.025	.000	
	stridelength	.000	.	.001	.298	
	supportbase	.025	.001	.	.009	
	steptime	.000	.298	.009	.	
N	meangaitspeed	100	100	100	100	
	stridelength	100	100	100	100	
	supportbase	100	100	100	100	
	steptime	100	100	100	100	


Variables Entered/Removeda	
Model	Variables Entered	Variables Removed	Method	
1	steptime, stridelength, supportbaseb	.	Enter	

a. Dependent Variable: meangaitspeed	
b. All requested variables entered.	


Model Summaryb	
Model	R	R Square	Adjusted R Square	Std. Error of the Estimate	
1	.944a	.892	.888	.06306	

a. Predictors: (Constant), steptime, stridelength, supportbase	
b. Dependent Variable: meangaitspeed	


ANOVAa	
Model	Sum of Squares	df	Mean Square	F	Sig.	
1	Regression	3.143	3	1.048	263.485	.000b	
	Residual	.382	96	.004			
	Total	3.525	99				

a. Dependent Variable: meangaitspeed	
b. Predictors: (Constant), steptime, stridelength, supportbase	


Coefficientsa	
Model	Unstandardized Coefficients	Standardized Coefficients	t	Sig.	
	B	Std. Error	Beta			
1	(Constant)	.613	.064		9.650	.000	
	stridelength	.832	.044	.671	18.877	.000	
	supportbase	-.443	.118	-.137	-3.756	.000	
	steptime	-.919	.053	-.603	-17.285	.000	

Coefficientsa	
Model	95.0% Confidence Interval for B	Correlations	
	Lower Bound	Upper Bound	Zero-order	Partial	Part	
1	(Constant)	.487	.740				
	stridelength	.744	.919	.744	.888	.634	
	supportbase	-.677	-.209	-.196	-.358	-.126	
	steptime	-1.025	-.814	-.607	-.870	-.581	

Coefficientsa	
Model	Collinearity Statistics	
	Tolerance	VIF	
1	(Constant)			
	stridelength	.894	1.119	
	supportbase	.846	1.181	
	steptime	.927	1.078	

a. Dependent Variable: meangaitspeed	


Collinearity Diagnosticsa	
Model	Dimension	Eigenvalue	Condition Index	Variance Proportions	
				(Constant)	stridelength	supportbase	
1	1	3.839	1.000	.00	.00	.01	
	2	.119	5.672	.00	.03	.65	
	3	.035	10.496	.00	.39	.00	
	4	.007	23.215	1.00	.57	.35	

Collinearity Diagnosticsa	
Model	Dimension	Variance Proportions	
		steptime	
1	1	.00	
	2	.03	
	3	.47	
	4	.50	

a. Dependent Variable: meangaitspeed	


Casewise Diagnosticsa	
Case Number	Std. Residual	meangaitspeed	Predicted Value	Residual	
56	-3.536	.47	.6930	-.22296	
88	-4.798	.32	.6226	-.30255	

a. Dependent Variable: meangaitspeed	


Residuals Statisticsa	
	Minimum	Maximum	Mean	Std. Deviation	N	
Predicted Value	.2309	1.1129	.6303	.17818	100	
Std. Predicted Value	-2.242	2.709	.000	1.000	100	
Standard Error of Predicted Value	.007	.020	.012	.003	100	
Adjusted Predicted Value	.2234	1.1059	.6304	.17805	100	
Residual	-.30255	.11492	.00000	.06209	100	
Std. Residual	-4.798	1.822	.000	.985	100	
Stud. Residual	-5.036	1.859	-.001	1.015	100	
Deleted Residual	-.33326	.11953	-.00009	.06603	100	
Stud. Deleted Residual	-5.840	1.883	-.013	1.073	100	
Mahal. Distance	.093	9.463	2.970	2.319	100	
Cook's Distance	.000	.644	.016	.067	100	
Centered Leverage Value	.001	.096	.030	.023	100	

a. Dependent Variable: meangaitspeed	


Charts


t=Ñª3«V­2­ÙnÐMºÇ'O:çÇÆÆéÈôÖzê)Ì_|qW£"÷"ZO[´L]iÒ&¨Rº©©É¹Þ4k/¾øâêÕ«C-,¨*RÊ­[·L|¬êÞõhñ«½h)çï9ìÕº©oÞ¼9kÖ,E$&ÒÒ§êYD7nÜ03X­V-ûÑGõ§FcÕj¦ÅØyC.¨/ï¼iii¿­]öäüæååiÍBÑ&97t<9ºBFôpµý5ã¿.(­eáÕè¦Èý¥VaZcLZvqù«®çÅÝ:¹ÒAt4ú©F6qTE©Éîîn3éÒx8ìÕº¯_zé%JJ¿õÖ[ÇmI±ªÇ¥O»ÝÞwµüã:ªît4ÝtÙAwÙó+ôÝ¢98nÂý.tÀÍ:¤#ÓïÁ×dLLLnnî¾üòKÆÕè¦Èý¥VÁ#­ªÚUfXXØ@nDåìã¡.èfðgßR¯¬ÖO?ýÔÔ×JëSéÓ§O;JÄèôÛhÒÎ7á8º û£7è.éüºt³¡î¥®Öe%çÎ3fuøïÃ?ôÊÕ8+­GZÝ½·ó%óÝÑÚwñóçÏIý©ïûOßÍå¸D9fµÒa¯vÐqÕªUáW^Ñ§ênç"3*XEúâ/<Ç öLÇ¿~£U7»ì-­ºÙÄ°÷Ý´êájû=k­­­[¶l1-´V«uW£"÷"ZAª]Õ,û÷ï71cX¬©¼òóóQÌâk×®¾üòKXYYY.8Ð71=X¦CëÚµkf=Òa¯ÖtË]ë¥ß£±gÏG¼²ûvç"=&~ÕFwîÜé¹Z]0--MGïúõëæè9þÍx¸ËÞÒªMßM¥Î¦®í£®!~Ï4=²ûjtSäþR@«ÐµÛÇèÊ¯úYSSã¼¸óÈRÕqöT«ª5u®ËØÎ®Öq¹wÈÕÅf_´uG¤Á¡Ã¬Y³î7:èÎª´£åÙÃ]þÊÀLºÙÄ°÷]1¥ó«W¯Òé÷¬99ÈÉÉáÕè¦Èý¥Va­ªzøáßÿÙ<h·ÛU§¨Û½·ËâÇW©æijjò|A70÷V»9ûÞ,8¼Õ8p@ókæîÒ~Ù´iË6Z,:>!!!/½ôÒçîøóá~£.¨½3·Ejµ×íË@»ì-­ºÙÄ°÷]|øá111Z§ ë<Ü×ÃÕº5/¿ürdd¤1±T ?Â«ÑûK­Â(¶!8cb;ã¶7o6vÍÆÕhé[uáÍ7ßäj´Ð?½p _®]»VPPm®%ñÞ®F@«hÐêÄåòåËÇÙ;sòäI3ôqñâÅÎ¯4´ýÓÔÔd2Ó·(##Ã¼8eûöíë×¯ÿ·û·())iMêëëÿûÿïT×hub°víZó¾Eaaaæ³7nÜP8û¯ÿú¯súñmÑ!!!ÁßßÅT×huBþ´êx8¸KÚ§zêÏþìÏ®gÎîåÉ'¤¢F«^«Î/'è´£DLLÌÒ¥KwîÜùçþçTÔhuÂk5<<Ü¼LCJ£UK§*HSÏ?ÿþûï£U´êZÝ°aÃÞ½ÐgFFZ1kû5qªªI´V'¼VÍdSSSdd¤yê·ãílhF©4..ÎáT´V'h¼Ûö»´SÑ*ZE«Ãlûuô§:ç£U´V¼ãT´VÑ*ÀHÚÕÕU\±qãÆuëÖQß¢U´08Î÷Ò3j)33³®®®ªªJ¥111Ô·h­§:ßKcÈÊÊÊÍÍuLþíßþí]wÝuøðaªVÜÅ©rªÕj]³fÍfâ'î¹ÄÄDÿïÿû*5³½ÿþûË-ûéOJVÑ*»¶ß3f,°®®îôéÓ,Ø¹sghhè_üÅ_È¸F«Ë/ÿË¿üKªVúoûÛËìÙ³MTZZZ"ÆÅÅ%''çåååää­Î3ç½÷Þ£ÊE«hÀ'OM6Ín·GFF**e322jjjTñOÿôOeóæÍ<ðÀùóç×¬Y#û^¿~*­¢U×8uúôé3gÎìèè0!©âÔ¥K®[·®ººÚhµ«««¹¹9!!ÁÏÏ/44411Ã¡U´àT5uêÔ³gÏjRî5)­Ê¯Ògeee\9§+<­¢U~*ÆÆÆ>øàÌâââèèhåüô§?UÑ´iÓJJJêêê233ív;o°A«h ï$?uê"Ô®®.GQý¬Y³ªææænÙ²EBMKK+,,ä6h­ôïTçç(edd¤§§wvv*ÝÓÓ#*0íîîhq´VÑ*À¿Ó÷9JRiNNÅbIJJ²Z­Sû>^­¢U´0¸S(ZmhhP +Ëº_	ZE«hÀÝ»ÞZE«h&;^q*ZE«hpê×÷ÒxÅ©h­¢UÔ·¥zË©h­¢U NõSÑ*ZE«0yãTÍm^èVÑ* U~*§*Nõ®SÑ*ZE«0j·Û½ÛöVÑ*ZÉ·îOE«hÐ*ÀdÇåy¿h­Zá·ýz÷^´VÑ*Z¼N½þT´VÑ*L"F»?­¢U´1èOE«h­ÀdSz×'>®©©©­­5ï0G«hÐ*N¦S"""233SSSCCC%W´V­Ðö;§Ùl6ÇR¬$VÑ* UÉyÞï°ûSµ¬Tê[\VÑ* UÉØö;ÂûS-KÎ®]»rrrÐ*Z´0é:ìþTçhµ±±Ñ9'//h­ZtNõÊ½4ô­¢U@«8õk§ööö¯­   44ÀhÐ*À¤nûõSZUuuuMMÍb_´VÑ*Ll¤½1x>ZE«V|ÅKNE«h­ÀÄnûËçý¢U´hÀ:òiÐ*Z´_·ýSÛÚÚ***ÊÊÊ²VÑ* UÚ~Fiiihhh^^^aa¡ÖÝÓÓVÑ* U_¦££c4Z__¯Õ*Z5]]]			[Ñ*Z´àËN¥q¿¹¹¹Î9uuuÉÉÉh­ZðMF©?Õ&º45ÇÅÅ¡U´hÀþT³ªúúzª,X¾|¹£X(xMMME«hÐ*¶ýz×©ZgpppHHÈ¶mÛ*++1cFKKiVd|äÈ´V­øS½Û§µµõöðÃ¨´µµ566ÖßßßjµÊ©.mÂh­Zðüy¿dMMô|æÌÝ»w:uÊ4óJ±ÝÝÝò¨yÁjOOÏìÙ³Ï;7ì/VÑ*ZS÷ìÙc±XÒÒÒ222¦M¼víÚ3gFDDÔ××h¶¬¬¬ÊÊJ³+£U´hÀ×âTÍ-¹oÍÍÍÒ§y^RYYÙÂï¹ç_þòZçßýÝß©¨ªªÊH411Ñt£ÖÖÖ*g$_­¢U´ã1NS§Û©WzoE-))1i­­±±±ºº:%%%))IAj^^^qqqNNþñÇWTTÄÄÄxþÆr´VÑ*ZNµÛí#iûíîîVô¹råÊÝ»wÅråO·¢jýÒ§ÚJZZÚÌ3|ðAyWéa?­¢U´ãßÚÒÒbµZ%Î3fdffÊ²²¦YYY©hÕx÷±^3T´VÑ*Z¿´··Û©Z¶¤¤dÓ¦M³gÏÞ²eËÞ¾U©			¹¹¹åååZsXXXuuµdÓÐÐP)Üë»VÑ*ZñÒö;ìiZ[[¥É¬¬¬çþî1¯ô>Õ!22rêÔ©+W®Þb¤;VÑ*ZqáÔô§jAó¶ÒÒÒ¢¢¢ÚÚZÕÑº;wî¶»ÆÆÆ¶¶¶a¼ñ­¢U@«ö§¶··+ú4¦¬ªª2½§ú4I°ÈGÑ*ZE«0IêÞ£¾÷ÞtZ[yyyVV«ð455577wÌv­¢U´·3NÉ»ÞJJJ¦L¢õ§äësîÜ¹þþþiiiV«5##CrE«hÐ*NuQ¦­­­JÍ7/<<êèèxüñÇyäÍ`JÇ´VÑ*L¼¶_a·Û_2SPP0mÚ´3gæææeVÑ*ZÛyÞï°ÚÜÜ^ôÏÿüÏÎù»vízægÆltZE«'OZ­VÿÅ=zÔ¹¨¡¡A¿RSÔÔÔVÆsÛïHÞõV\¬x4//OáááJ8<ZXX¸yóæÛ»wh­N$222öíÛ§ÄöíÛ×¯_ïváÂ%ôuëÖ­3øÎw¾Vn»SGÒZ__ÝÖÖv¥·?511qÉ%æU°í(=ä­¢UßDî/¸qãÜéêÅÐ§ÒW¯^½£wÞy'Z¸½NvjiiiJJÄ¹|ùrãN©©©©sçÎ½çrrr,Ë=nû>¢U´:ð÷÷ï7mÚäN~òÉ'²ïÿ×Gy­Ü^§ööö¡.m³ÙêêêÖ¬YóüóÏË ÁJ»wï3gNyy¹	aÑ*Z!àçççH8­X±âìÙ³Æ¯«V­¢o`|¶ýÃ©&ÀÍÏÏ/,,¬ªª3¥»víRÌ:~ö­¢ÕDxxø7L#°Ò²hàö"#d"QÙÔ¡g)VZ5ümhh5OÕG«hÌöîÝ«>322¡~úé§J>Z+Z'(<]ÚË0ÚÜÜ»lÙ2-îxÁx]]T:uêÔåË+QYY9®ö­¢ÕDSSSdd¤Õj=qâÄ¿;¾>;²©âT*VÆIÛï0Æ(µµµ)-++1cFQQÑï½¦L3ÃÖ­[,YÒÒÒr»ùVÑ* UÛàÔaÜK£ðT1hfffPPÅb1¼%%%sæÌQÎoûÛüüü±*!ZE«VngÛï0ªÕf³i¬ZÖ¼6UzVQuuuTTÔòåËLZE«VhûuÚØØ¨Ñ%òòòM©BXÉuï;ZE«h¼IGGÇ°ù`±XL¢»»;88XrÝµkWNNÎÞáÄ*·m¿h­¢U§Ü¯s´z¥÷¾«Õ®hµ¹¹YÁ«"×ñÐ*ZE«à×ê£oÕLæææN:5   ..®¤¤äö¾­¢U@«cÇH÷ëTj±X233SRR"""ß¨:!@«h­Ú~½âT¹oµ¶¶¶³³sÂ´VÑ*Ô©#éOõ1Ð*ZE«0|Fø¼_´V­q*ZE«V¼§Úl¶èèhÉ£VÑ*ZÅ©rªâTVÑ*Z:Õn·ÓöVÑ*ZE«#Å[÷§¢U´h`²ÓÞÞSÑ*Z´à¶ßÑ»¦¹¹¹¼¼¼¬¬ÌñL`´V­ø²SG¯?µ¸¸8444//¯  Àjµ*1!ÿVÑ*ZE«ÃaTûSëëëµò¶¶63ÙÙÙ©)lE«hÐ*2Úý©¹¹¹åååÎ9µµµÉÉÉh­ZðÁ8uïz´´4¬.C«hÐ*N2ùùùEEEÎ9»víJMME«hÐ*m¿ÃwDDDUUlhhu_Ñ*Z´01Ïû³ûSëêê¤Rm.!!AÊÊÊèÐ*ZE«à>ý»ÞzzzZzéîîÐG­¢U´ßpêô§ú0h­¢Uø§òlB´V­xÍ©¢½½6×ÝÝ§ÈØb±$$$ÔÖÖ¢U´hÀ×Ú~ÇÆ©"333))É<¦¦&44´ºº­¢U@«óçÏö¥®®®ÆÆFÉ[MMM½ç¦O¾wï^ÇuuuV«­¢U@«§KSµãøfñîîî#G(îlmmUNmm­<ú³ý,88811122ÒÏÏoK/ßûÞ÷oÞ¼Ù±6iµ³³­¢U@«¸íwHc¦,--#""BCCçÏ?cÆ­gæÌFYYY³fÍò÷÷â'49eÊ¿ú«¿qUúùZð­·ÞÊÍÍÕÖcÚ¥g­JA-ZE«V&ªSt/MYYÌ¶råJ2//ïJoé=÷Ü³`Áÿñ?þ/VNsssxx¸*wÚíöÊÊJmhëÖ­JkI·  Àb±(¢ÍÈÈ0]ªÅÅÅ			4£U@«µíwHNUêxÂÍßýîwZUZ!fzzú~ðÔÔT¤Êyúé§KJJ:::¤aéSÈ«W¯Öæ®ô>ò7''G*Z¸p¡ô¬Åm64VÑ* Uoû;SRRBBB*íìì4cdÊøøxÅ¦Æ=ôyb¾Ñ+Vúj+±±±---æÉM¬ªªR¨jºTßï½ÀÀÀç®¼¼ÜzUÑ*ZE«;Uq¤l*GÞwß¯¾úªâËC)eÊ»ï¾;))I³IúqÉ¾J'''§¥¥+ ÖZ*!!aÇ*=räÈ]wÝô«_ýª´´4""bB?þ­¢U´V§vx>îWñ¨Íf3éMMMk*è5srr^³fÔ(AJ~øaLLÌúõësðàÁ3f,Y²Äô¿®[·îÎ;ïeVÓ;«ÖÐÐÐàG­¢U´@jÿ¹ô­ÊçÎS:mÚ4ý ¤Ì)S¦ÜÿýEEE>ú¨¿¿dd¤¬¨Tf5/¥z¥áÚÚZiìE«hu¡ß§¹M¿À¨¨¨>ú­qªÛÜÜÜÕÕÕÓÓÓÚÚÚÒÒ¢tEEEnnnaaá»ï¾«Ä½÷ÞûÐC9bJ)Öb±¦§§+]RRR^^~âÄ	)Si³¦q¸±±Ñ¬Ù7^JVÑê¸fëÖ­wôaûöíh`lúS%Q»Ý®°U1åôéÓV*¦|àôOW3Hë×¯Wú±ÇÛ²ebÓððpÇ;ÆùË_Î7OÿÇìh­Â È£¯½öÚÕ«W¯]»öÊ+¯h2((­xÅ+322S*î,((xäGfÎ©øòñÇGòh6¡³fÍÝ¼ysZZlZ[[«Ïýû÷[­Ö#Gh%Ë-Ó"¿ÿýïM´ªL2ZE«ãN«ß8¸wÜVFNwwwbbbJJJÅú¹É¤>ýýýUôÂ/¬X±B1¨P)s×®]mmm*UHÚÕÕ¥É)öJïóde%JJJ4ÃÝwß-U766rÑ*Z_ìØ±CÕ?hª·nÝ2mÂöìA«#AÍÉÉQèyï½÷=V9III¯¼òâË°°°Ù³g×ÕÕÅÄÄ<ùärgss³òï¿ÿ~ó¨¨(Å²¦ÿuÞ¼yÒçÞ[QsssÍÊ333ãõ2h­úÐtZ6j\þÊæ£GÎ3Çf³É,0hiié·¾õ-ý©UZ9Ë/7CsMD[__¯Éz(00P~MMMU¼kúeÍ9Îh­#üÜâïïVÂÐèèh3¾7!!Aþ+++[¼xñÔ©S,YÒÐÐ0þ|VTT<óÌ3hcc£ÐÿøÇò®é­¬¬;wîÝwß½uëÖ_~yÆJ<yÒÜºjh­ZßGÑ§ÂPÎÎÎÎÍÍmooS§MöË_þ2??_W¾Ð¶¶¶ÄÄDµªªjÖ¬Y*þùçÃÃÃõ§Vh%¦588Xë5kôQEÀ)))aÀVÑ* UZu<´ABUä"­¾ñÆÓ§O,ëëëøÃ*½lÙ²?üá@.CIIImmí?ÿó?õÂ_´V'*'NÐ/ß¿ùóç>­çGJ«QQQÒj@@ÝnÏÈÈHKKS©âÔ§~ú±Ç#E«hÕ×¨©©é;déÐ¡Ch`Øâÿîw¿[°`Õj1c¡'ZE«°°0sÍ­[·nÞ¼©&ÃÃÃÑ*ÀHxåWîºë®ÀÀÀ'|»KÑ*ZL²n°6çÏÒûS­¢UVóòònö¢&###Ñ*Àèéé1ÃwçÍ7eÊ%:::8,h­N:>üðÃ¾«có´¾DZZZrrrCCCLLÍf[³fMjj*­¢ÕÉÈ©S§æÏ¤Ä'Æf»h|'O:ÞÚÝÝmµZéUE«hÆ´>ôYQQñøãÇÆÆÊ¬Îý©YYY"´V'QQQªÎ;VJGGG\ê/¾hîü~õÕW¥ÜWVÑê¤ÃÏÏÏe$0Z(0&«««ÿÛûoùùù			Eáéïÿý76mÚ_ýÕ_·¢^éáLtttgg'Ç­¢ÕÉÅÞ½Í«W¯^½uëZèÉRLNNþÞ÷¾§?£111khhèúõë§Nzß÷ýÓ?ýfPðºzõjÅ¯òkss3Ç­¢ÕÉw(y1ÀÀH¹¹¹>úh```IIr4ùì³ÏJ7nJ%×¬¬,uuu=ÿüóÄ©h­NÞF`^Ð/UUU:eÓ|PUNBBBKKKGGÇ¬Y³ôcQfýÒ¥KÍ"·¢ZE«@ß*LjzzzZ[[¥L%wþã?þãÌ3T·k×.E®eåÊJ9sfêÔ©óæÍVåÝx ½½]ö_µ,­¢U@«0)++KIIzè¡ÐÐP/ÁÁÁ6Mqqqmmm&`½ÒûÌïÿû=öØ¤UÅ¬U¶úûû+®ÍÎÎ¦í­¢ÕÉËo¼rÇw¨RØ±cZFòKLL&£££W¯^ß÷)=cÆùóç9rdÿþýÓ§O=¶¢UTÊÛ¾FFFqq±ÌªØTÒUÀúôÓOkf???ù¸¦¦&55U&&BE«hu²óÖ[o¹S³¢UvÝ_üâbUU&öYýbEuuµ*¿nÞ¼9==½¢¢¢´´T¦ÔZ¤¨¨HsJ½çÏOJJÒ²Ê¼ÿþû§L2mÚ49UnÖÕ«å]VÑ*|¥úB*Õÿq¥õ©´þ£UèÈpùùù111eáÂ³fÍR(éïï¯¾¶¶¶­­Í<_3¬[·NUN``àâÅÐ²X­V«n¥ØÍ¶qãF¹³±±qÙ²eR²VöìY5ZE«ðTÑH¥æFÕ7n(=6£Ñ*ÝÝÝ¨"Èööö»îºKTT*SÖÕÕEDD¼ýöÛ³°°ðá.++»Òû,¤zH3hqIW³uvv*xÍÎÎ6¯2´~2±Ö¶dÉÞõVÑ*ôw¿ysêÝ«VaôåÈ¥M»î®]»V®S*-**Òõ¬ÒM6)JãââÞxã¥·lÙ¢5++K³EGG×ÔÔ´*IóþT´VaÔµzòäIÅú#¿xñâ£G:]¿~ãÆóçÏWVÁëÈp:SSSçææ.X°@1¨¹%FlkkbÓÒÒòóóëëë×¬Y#Ånß¾]¦T©Ô÷Ýw+4eÊçî^Ðu«Ù´r¬Z|ïÞ½8­¢UnðP±ûöí3½³ë×¯w.zýõ×ß|óÍ[·n×O¢UUUUúT¬iÆÛÚÚ*·ÕÖÖnÝº5,,L*­««Sè©+Va¨¤øÈ#º*êèèÐ??ó@ü%K<ôÐC2åwÞ©ÈjÁéÓ§Ûívªæb.ëEÛÒ&JJJÊËËu/í§¢U´îðsý¬ª×½³QQQÎE_Ï=ëü×ýWK´´ý"&&&JfYYY÷ÜsÏ´iÓ¾õ­o)¢PR®eJ²¤wßWQæ¯~õ+¥õWO²Ô"¦ôÌ3³gÏ^»v­bÐýû÷ûPã*ßÍ×hoow¼?VÑ*Å¸§~ÓfòwÞ	R­têÔ©û·¯ñññhú%!!¡   §§GbSôùÝï~WÑ§bÐ»ï¾åÊý×-¶´´(ë­·ÒÒÒ®ô>IfiiiD/[¶l1OlPD¯9ôd~Ñ*Z±yiÅ.EöìQâ³Ï>³Ûí4ÃÚ~%33òHa¥¼ØÝÝ-S>òÈ#UUUÍÍÍ.&5$úøã§§§kNiØìÕâóæÍûþ÷¿_^^~øðaG»îPÃeVÑ*5ááá7nÜ0ÀJ»È¢UpOuuµy!Ì?=W¤¤¤ï½×´ÙJxúßV__ÿï|'88øã?hÍ`%iU®L<&húSÑ*ZÛÀöîÝûUïK[322Tîß¿_O?ý411­'ÈëÖ­=¶/M'hWWL©hµ²²RîÓ§rì1E¥ñ1mÚ´IW3(®Õç°¿TªõãT´Vá6ÐÔÔéçç§@áÄÿ~nîøúì|ñÅéééSívû¹sçÐ*xÒ)¥¦¦Ê¬óæÍ)ß~ûíööv)VÿÌ¾ûÝïÖÔÔH-*--Õüï½÷ÂÖùóç«ôé§~üñÇµ¸äª`8Ð*Z Up&999??ß¤JwÞyçÊ+gÎ9cÆ&%%M>=((È¼gÆÜHãÅ/ Úl¶èèhÉÓVÑ*?âSÄ9ûVÑ*x±ùWtyfVVÖO~ò#9Å²æÖ^FÒÚo*§*NÅ©h­Â0ÏÜ7*ê«h¼|©0Ñ%³°°Ð´÷6R©Â_Ú~Ñ*Z/ §* n/111uuuI].9£÷§¢U@«Þä×¿þµ´zðàÁ7o¢U¸ÔÔÔ8ÞÚÞÞfî^=´!V­zU[!!!Ã&0ZïR__lµZ³³³½ÛÚoÛ/÷Ò U@«ô­xÇ©ô§¢U@«ô­¢Ë¯µµµ¥¥e´|ÐV­Û¶mV>Lß*1íííÍÍÍæ¹HæVT%¼+ê@ÛÅ©hÐê¨Ê¼o­ÂðPl*ÆÅÅÅÇÇßyçæÝm¢®®NfUé¨Æ©¼ë­ZEFò¾U´Ã ³³Ójµõôô¤§§Øl¶=öÒ¢¢¢ÜÜh­ZÁ[_%ÑoûÛRV'ÕÕ××Ë¬f¥SRRhû´V­;L¯"Ñ5kÖ$%%666_é×­¼¼ÜÑ&ìEÌó~q*Z´Jß*ø]]]ÅTkaaaee¥ÕjÝ±cGLLÌáÃõiiéõQKæÝ88­ZÓ¾Ug­Ò·^UMºµµÕªR]KKËo¾©ëmáÂééég+**¼îTúSÑ*U=Z½=~ýW^V8VÁÔÖÖfff:&«ªªdÖðððþðÞw¾óßüæ7R½þöîO´Vo3·nÝVUß¡Uð"íííÅÙmøë¿þk²£ôãTaGZ´zÚÔÔDß*6­±±Qé¥Gõ¥o¶_VÑ*Z½ýCô­×)++Mãââ½),f U´z,9SoÜ¸VÁ[´¶¶VUU9rd´ßHãhp^ÚN´V'hÕçéééIKKÈÌÌLLLÔ?¶æææQÝ"c­¢U´>K~~~rrrWWÜµkÕjuLS¹Ð*Z½m;v,,,ìÂÏ?ÿÞ¹sçÐ*xÅ"Õ9ç¤¦¦VVVRÛ/N´Vo2¨¤È9þ|yéÒ%´#¤³³3::Ú%³¨¨¨¸¸¶_@«hÕ×0½|ù²#³§§G5ùhFNhh¨Ëý-iii^V;::p* U´zñ÷÷>¯]»æ/ËòðBðýö­*õ®S÷h­­öpÑ*x¾#Íã ¼ý©VÑêxAAôùé§ºä=Vùh¼Ekkkeeå#G¼;þT@«huñë_ÿÚN:~ü¸yøÃõë×LæîÝ»Ñ*gèO´VÇë×¯ï÷ÉiiicóÐ*Û©ô§ZE«ãÖÖÖEøùùÍ?ÿèÑ£c¶u´ÃçýZE«V8Ð*Z´ã,NµÙlÑÑÑ^Û9 U@«h&]*§*NÅ©VÑ* U©Sív;m¿VÑ* ÕÉHnn®DPPP0ÂÛU¹?Ð*Z´:yéììÉÎÎniiinnNOO_mV§ZE«áºÅÏÏ­ÂðÈËËSsRSS÷Öî¥´V'~NôÕ*Ïa ´¾¾>66öþáókkk³Ã©ô§ZE«·ß~[íµ×®öòÒK/irûöíhÜi±XCCCïºë®üüüSTSS3T­Òh­NTe£Éàà`´ÓÚÚªkFú4ÀßûÞ÷Ì`%S2¤F`úS­¢Õ	¯UÕ×®]»~ýzFF&Ð*xNIIInn®IwvvÚíöÕ«WGFFVWW'&&J«YRÊ»Þ­¢Õ	Ì[o½Õ·ouçÎh<Gê®]»¨Dëçç^^^îhÆ©VÑê¤ ¦¦&*** èèè±Ù.ZõÊÊÊ²²²sêëëm6ÛVBÛ/ U´hu²ÓÖÖ¦ÀtãÆ¥¸¸Ø¦­­­:÷ìÙãùzÌó~q* U´huò¢488877w×®]O<ñÄÔ©Sï¹çÄÄDez¾3gÎp* U´ê;|úé§-RUha·ÛÑ*Jww·Õj5£,hjjÒ3èO´VC¹<ôôt´î9räHrr²K¦Î¶¶¶!9þT@«hÕ§GÏ=ë¬Uî[A©®®ÎÌÌtÉLJJjllSE;ÇÐ*ZõCÙIH«·nÝâÀÐ/''Ç¼fÃiii÷ÞïÔ©SKJJ5©?d¶;Ú~q* U´êSÌ5K=qâ±©I£U¸Ò;Ð·¢¢bóæÍæ¥4Û¶mó÷÷;wî±cÇ$×éÓ§§¦¦^ùÓ5ÓÕ?1JVÑªo¢k®ïã jjjÐê$DëééÙµkL)GÆÇÇÊ¦Ë-		D»»»Í3¡°°Püô§?UÌªÙ4¿ó!Ü¾KÁ©VÑªorüøñèèèE!V«õ£>í¢ÕñCiiihh¨Î~pppPPÐîÝ»?øà%,KUUUBBBSSSJJÊøCÍ#õÖÖÖÊ²fÙÏo§a U´hÕÇQ)Õ566Ju3fÌÐyILLÜ¼y³âÑéöÁlnnnkk;wnDD´Z]]íx)M^^^YYNå^@«hÕ÷,9Pêîî^´hÑüùóÑêäA¨¾ëx1ªÝnòÉ'+**V¨ú£ýH±©lªX6))éç?ÿ¹2ËËËM®"ÚOÚ~q* U´:é´zóæM^c>©èììVMÚÑ´[TTô½ïÏEbÿûßË£ñññ±±±o¿ýöÔ©SÝ8p@ÞÕ²¼ô¶_@«hÕ÷QHzÇpßê$AduuuHHÈéÓ§¯ôYTèýÎ;ïÄÅÅI®³gÏîîî®©©>úªU«ÎnÙ²å^HIIÑlÎOYh­ú>.êx¾   mÛ¶¡UgóæÍE6mZeeåÞ~ÖY³fiòW¿úU^^®µ	x[S÷h­N"äÑ±iòE«ãéS¦4ììì¼ï¾û¦NªÚ'33388ø±ÇKMMÍÍÍUäªPU3;¦?Ð*Z´êûÈUUUÉÿôþÓòåË÷ìÙã­ÇÑh­NR¶lÙfÒTî9´êÃÔ××Î3§¤¤Ä¼Õâ­­Ðh­NRÒÒÒ®Ö´ênÞ¼ùMHHp4ðåææzË©ô§ZE«yôâÅfRõ 7Øø*gÎQÚ<çá±ÇËÎÎÖdMM&[[[G¾÷h­NjåÑ¦¦&3yìØ1n°ñU6÷âÜ³gÏôéÓõJ´Z­õõõÄ©VÑ*Õ­ï[Ý½7Zõ=òòòÌtwwµ´´8w²$NµÙlÑÑÑ+GÐ*Z¼|ôÑGQQQ½(j9xð C|95))É9§²²2!!Á++JåTÅ©8Ð*ZÛZcÚíö¬¬¬öövó8ýª««óSµfÚ~­¢U'OT½ìïï¿xñâ£GöáÐ¡CwÜqZ½íH¢UUUVuÖ,KbbbCCÃÈWÎý©VÑ*|O?ýtÑ¢EÁÁÁæ6.±oß>%¶oß¾~ýzÒ7oÆÇÇ£ÕÛbS34WBINNö¢ÿ´rh­k@éx2ðW½·®¦§§²lXXØ­[·¸qãFTTKé/~ñ­[·­~ùåOö!22­Dum6Û3ôQ3(I999æÕ4^iûå^@«h¾Ü&ó=ÖY«Þ`ã|«Ë­®.Ô+é­þÿó²ûp÷Ýw£ÕQ¢®®Îb±ìß¿ÁK,Q¨jº»»#""F>°þT@«hú;½|õ§¯:¿ÕÎ³8)N:vìY-ÀcOLLÌ=¨®®PÍKU£FØ«J* U´ý3kÖ,ïÄÆ¦&îÉ²íÆ¦ØeaÑêØÐÕÕU\ô_ÿë5#$?%JKK½­Òh­Âèëû8OÝ°aÃÞ½Ðç@&Z3äËßüæ73gÎrejjªj·ÛåÔÿøÇeee#ï[UÊ»Þ­¢UpÇñãÇ|øûû[­Ö>úÈÃ"##ãj)¹ýz­mmm?úÑxâ	34)))éç?ÿ¹,[^^n^zªÓ¤(Öv¨S­¢U¿ U¯ÐÜÜ¬Eqqq!!!AAAIÙÚÚªÈuõêÕúß#éVUU566û9´ýZE«V©Nú4­»M8 hÒô¡*²Ô3gNvvvMMÍ7d÷S­¢Uýû÷+ñóóó÷÷jhh@«ã¹S,))Ù³gÏÉ'®]»öáîîîV©ôYXXX__¯ÈÕÌ©oWæþT@«h<âí·ßî;dIµ6ZotuuûDeÊÔÔÔ;ï¼såÊPcccå<OkDDÄæÍ;;;óóó£££G.BúS­¢Uò¨jêk×®]½zõ7ÞÐdPPZodee)úìéEá,ËÏ~ö³ÜÜÜ¢¢"ùUù---rí)SBCC322FþÌîO´Va8ZýÆÁå5æãE¢AhllLLL4m¼[·nAûÛßJ~V%?ùµ¤¤Ä+[4N5kæøZE«à;vìG_zé%ª/_ÎÉÉñü¾U´:fÇ$tuuµªÔââbóÂ3g>úè£Jäååyåä¶_h­ÂP¥[<|!Zõ:]]]ò¥þß|ðÁ)))I§Lòøãwtt´´´(;ªªª®ô~÷ÝwÛÚÚ¼²uE½Q´Va8ø¹Ååúhul¨¬¬GSSS×®]çw>òÈ#V7oÞ<gÎyóæIuL/^¼páÂîîniÎììlom]^ÚN´Va"VûåÈ#rªy~rrò[o½ §j2//Oÿu¢z			ÑlX-KVV¢[¯l1JVÑ*Ë/÷Í¼xñ"Z½]äææ:ÆEDDôôô477ÇÄÄêêêoë[(ßÑÑÑØØèEÿq/ U´^è[Ý²ecòÖ­[6lÕ.U´Ú/ÒdQQÍfÓÁw¼ÖÍjµÃÙªªªÒÓÓGãGãT@«hF¿¿¿ÌváÂUåf¤7Øx¨º»»MkmCCCYYYyyù'Ì|ðÁU«Vµ¶¶æççgeeÉpAìììúúzéÖ¨×n·k)¯CÚ~­¢Uð;wî4w¯ÈÈÈÒ·êdPy144TÆ9sæ=÷Ü£´ÐéÓ§Ï3GúüÁ~ ØTùÏ<óþÊX,ºº:.""¢´´T9çÎàtvöYåÈµ999^¹Æh­×8pà¢U£UUÜ§NB«^!99933ó|/ê¼yó,Y2öìÚÚÚ¤¤¤çÞ¼^FEæTIîÈ#òì»lÙ2Éõé§ÎËËµ×¿¤6Ç¸_@«V½FPPyZáñãÇÍ£!*w´:BäH¹ÊD¹¹¹ì÷Ý×ÚÚZSSc·Û%TÍP^^þÐCI3##ÃôªE©FðÖ_úS­Z£!K/¿ü²còæÍY6æe2---YYYÏ<óLKKe¯ô¾]ü®»î23ë¯&¨¯¯_½zµÂÙ¶¶6óà$eÊ»Fº£÷méO´hÕûôÍ¥KÐêãââd©Ù³gÏ9óî»ï)çÌ£æææììì]»vi6÷ß¿<ª0122R*¥RÅ²¥¥¥òèÂ_õUå¨Èx ?Ð* U_cjUªªª*))©®®þøÂÂBýh8p@¥Ú£åËGDD466J¯¼òdYYY)éJúÜ·o|öäOÊµ²©Ä&·¶¶*0Uk±Xì1å+ÞU§ÒhÐªÛ~-½.ÏþíGOh­9rDÊÌÌÌT@9wîÿýìgAï»ï¾Ó§O«´««+??¿  @ö&ï½÷ÞÍ £úÂ/TTT($2eÝn_²dIppp```bb¢5xë¡¾îÿð¼_@«VÑêíäÌ32å5k¦Oþê«¯^éÚäôÒK/Ér§5:ºKSSSP¼jÕ*E·¿ùÍoÚæååÉ¬ÍÍÍIµ ÒæÁI£½#Ä©V­¢Õq¡*<PW¬XaµZåÈììì=öHæ¤ò®i¢Uw·ÊÊÊqÒ|m³ÙômGþzs´V­Eæl:RQii©ÂMO¿óï8÷êG«ØTMxâéKc£zçqªª8§Z´VoSåÎ?üáqqqùùù²ãï½ Yæææ>úè£YYYÿ4D®@öÇ?þñ´iÓ¥Û1ë.õÄ©v»¶_@«VÑêí!99YN5£UPúòË/KN999ÿù?ÿç-[¶ÜÝËÆ%ZÅ¦aaa?úÑÊÊÊZZZÆ²»tP¸?Ð*u>Z­º­7²]é8¾âQÉµ®®.--­««ëûßÿ¾bÓÙ³gËUP5ÔkÞ:ÞÚq* Uê|´:êø¹ÅßßkÕñF6Q^^®ÉW_5==]~MII_;::ÆC$:hÛ/÷ÒZE«hu²0µjÞÈæÜ·o_PPÐÔ©ScbbòóóÇÃ$OJ*ZE«huÙÙØKEEEIIÉ(½mô ?­¢U´:¾0Fïl£ý©h­¢UðZÊ»ÞÐ*ZE«SÐ*Z´JÛ/ZE«Vó¼_VÑ*Z/´ýr*ZE«h­zÇ©ô§ U´ãQ«ÝÝÝEEEv»=++KÆÿN¥?­¢UZíééPÓÒÒÛÚÚCCC[[[Ç¹SE;µ'ZE«hõvjµ¡¡!==Ýf³%''?óÌ3©©©sæÌùè£ó¤¤¤ó¶_VÑ*Üf­VWWK¢»víô¤Ê¬6mRZUUefëêêÒä8¬)Î?Ï%´Vaµ§§G²<räÈÞ×[­ÖÓ§OësÍ5555bUÚÝÝ¥·«ujUáéÒ^p*ZE«pûµÚÚÚj·ÛMº¼¼<??_I7!!¡¥¥e|63F	­¢UwZZÍËËSBrÝºu«²tß÷ýýßÿQQÑx²Ä½4h­Âxì[µZ­uuuF±ü±>¡vwwoØ°aÚ´iK,ÉÌÌlkkWm¿8­¢UGZíêê*..NOOÿö·¿ò³ý¬¾¾^áéÔ©S×®]«´"Tùµººz¼U´ý U´ãK«öâââ*N­ªªZ¶lDõÈ#dgg¿þúë999©©©¹¹¹¦WuÑÑSÐ*Zq¡U©(++ËjµN6mîÜ¹ÍÍÍ&ßMRð:Î+9q¿h­Â¸ÐªT£´³³3<<üç?ÿ¹üêG«««ÓÓÓÇs@*ZE«0´ZXXiÒ]]]EEE&§¶¶v<kþT´Va|i5--Íø5éÍ7·µµÙl6:néO@«hÆV³³³÷ìÙãþ°>ûì³<ð@cc£âT»Ý®øu|:þT´VaÜiµºº:..Î!'5**JrPÆ§SyÞ/ZE«0Nµ*òòò¢££KKK+**Íã	Çm@VÑ*k­úúúÂÂBùUf5ÏÐH¥6M$WjC´VajuB Ê©Sq*ZE«VGêT»ÝNÛ/ZE«VG÷§ U´hÕ;´··ãT´V­z§íiÐ*Z´ê§ÒVÑ* U/@*ZE«V½ý©h­ZõZÊ»ÞÐ*Z´SÐ*Z´JÛ/Z´Vó¼_VÑ* U/´ýr*ZE«V½ãTúSÐ*ZÁ9yò¤Õjõ÷÷_¼xñÑ£GN8¯"éD³MZ­r*ZE«à)ûöíSbûöíë×¯w.=~ü¸º¦-ZtãÆ­°Ùl¾­UãTÑÞÞNíVÑ*BXXØ­[·5£¢¢-((èÚµkúìÃZu´ýâT´VÁ#üýýûM;óÉ'lÚ´i²5?1Jh­ÂÐðóós¤úÎpõêÕªJ«OöSÐ*Z!~ãÆÓ¬´KéÅ7nÜxéÒ¥÷I­2F	­¢U&6lØ»w¯úTTêÐÐ°fÍË/»YÜ÷´Ê½4h­Âðijjôóó³Z­'Nø÷ssÇ×g'**ê'&VÛÛÛq*ZE«pÛð%­ÒöVÑ* UïÐÑÑSÐ*Z´ê§2î­¢U@«^þT´V­zúSÐ*Z´êµ¶_VÑ* Uï8þT´V­z÷VÑ* UâT´h­³8Õf³EGGK®ÔVh­ZQ*§*NÅ©h­Z©Sív;m¿h­Z)ÜVÑ* UïÐÞÞSÐ*Z´ê¶_î¥@«hÐªwJ*ZE«V½ý©h­Zõô§ U´hÕkq*ïz@«hÐ*N´VÑ*m¿VÑ*øVÍó~q*ZE«V½ÐöËý©h­ZúúúÜÜÜÔÔÔ¼¼¼ÖÖV÷N¥?­¢U@«®ôôôÔÔÔ<ýôÓóæÍ«¨¨"""2+÷§ U@«hµÌÆ~üã477"ù5!!a §öövj´V­þªS#Ñ¼¼¼ÊÊJù²««ËD±¡¡¡&Ý·í§ U@«hõ?^>.//7~MLL<räÁjµvvv:ÏÏ%´h­öOss³$jÒR©hVVbVåÔÖÖ*Ç1³ÂÓ¥½àT´h­ö$j±XñhNNÎ<0wîÜßýîw1112«£í1JhÐ*ZÜÜÜäädcÖ¥gÏm³ÙRSSëëëNå^´h­Tª U1kRRÕjMLLtqg;N@«VÑêÐZdP3|Ém¿hÐ*Zõ8­ZE«Þq*ã~Ð* U´êèO@«h­¢Uï@* U´V½ÖöS­¢U´ê§Òh­¢U/Àó~­¢U´Jh­ÂxÒªTj³Ù¢££%Wj@«h­(NS§âT@«h­Ô©v»¶_@«h­öövî¥´VÑª´júSq* U´VGªÕ3gÎp/ U´^Ð*Ïû´VÁ;Zíìì¤íÐ*Z/h¶_@«h¼£UÞKh­w´ÚÙÙIh­´ÚÞÞN* U´^Ðê3gú÷ÛÜÜq­¢U´:½ëíÈ#¥¢¢¢§§GE999§««ªÐ*ZE«¶ýô®·ääd9Õ9'))É%Ð*õ-ZE«ÿÑöÓïiª*NuÎSsss©J­¢U´ÚOÛ¯j³Ù$×~gP©bYçâââÂÂBª@«h­~OÞõîX;;;­Vë#G¨J­¢U´ú¸×[wwwQQQbbb||ü9s/_^QQQRR"§nÞ¼zÐ*ZE«ßSÝ<óA±©ÖÜÜÜÖÖöâ/ÿà?ÈÏÏ¯­­¥´VÑê7ºdÉÙ³gK«			6lxòÉ'm6[rrò³Ï>¡õÐ¡CÎMÁ999TVÑ*ZýVÏ9cµZ§NúüDhIIÉ´iÓâããÖJoÜ¸Qfýá(¹:úP[ZZìv;Õ U´VÿÌ9N1cFAAiì;¥Ìââbéöã?¶X,ßýîwkjjª««ãââÌ­­­hÐ*ZE«ÿ¡U9Õ<óaÎ9FT¢¬¬ìGÉËËS:===;;;%%EÒUÀj¨D#0 U´_kU466nÛ¶-**jÑ¢E¥¥¥!!!fä#-//VóóóÎÈÈPj,)UüªÐv E U´:,ïºë®+VûùùM6-77766Öb±deeuww;7+65ÀgÎQ~'AAAg333zXZE«'OZ­VÿÅ=zÔÃ"²éý÷ß/5*ÜT´ºvíZiU×²¯¼òJIIÖà²°qãFÍPQQá<d	­¢U_ ##cß¾Jlß¾ýúõnþïÿý¿÷A¡çôéÓeÖ6üÍßüÍ©S§fÏý?ÿçÿüù²î²eË´ÇláÂIIIëÖ­KNN^´hQJJÊßýÝßý`0Þ÷]´V'aaa·nÝRâÆ7Ý]½zõ>ÜÙâÑ)S¦L:U	3ipNOP´S~~~þ>ÎÏï ¹8]¢¾½Ú;ýA§ºF«]²ý¦ûý¿ÿ÷ÿ.öaÎ9Ï<óføéOºeËÿý¿ÿ·d|óæMåüíßþíO>9ÑOyy¹Ë¿ãøþý¯ÿõ¿|xÞÿÞÁçÞwðç?ÿù÷ÝGuV'úëxXä`ÝºuF«¾ZE«h­¢Uááá7nÜøª·¥WiÐ*ZE«h­¢Uè6ìÝ»W	fddxXVÑ*ZE«h­B?455EFFúùùY­Ö'Nüû¹¹ãÐ*ZE«h­¢U-Ð*ZE«h­¢Uðµµµuuu>¼ÿò/ÿRQQáÃ;xéÒ¥ÿò_þË+W|xÿæoþæìÙ³>¼¿ýíoÿñÿÑw°¹¹ùÝwß¥¾E«h­Âh3Â§Oô<qâD||¼.]ªÙ|o2Ö|o¯_¿¾qãÆùóç744øÞj§l6)jjjÐµÍåËûÓà3UZ¯ñüéÁ>¹±±±ÇWâý÷ß_´hïí ¸yó¦þ:Lh­ºÙÁ×_ýÍ7ß¼uëôã;váÂ%ô9¡ÇÙé?þôúLUVáß´>=Ø'÷Ñ   ÜÁ_üâ[·nÐZu³o|`àÔ/*¡Ïû¿A¬]»öÜ¹sý^>SÕ UøÏìûèàO>Ù´iïí B»Ý®:kBkÕýUúÎ;ïè/sêÔ)ßÛÁ'Ojïtúô©«tÂ¢¿ëÐgª´_3Â§Oô4z5##ãÚµk¾·éééÇ¨:ó«tÏ=J|öÙgúá;¸bÅË¯«V­òI­úLUVákFøôà¾_õ¶­mÜ¸ñÒ¥K>¹.¯üóÉ«Ô7;èc¡ÏT5h¾fOèûØÐÐ°fÍË/ûêIt_ùÀæååíß¿_O?ý411Ñ÷vPªvMÓ§O+rõI­úLUVákFøôà¾QQQ>Ì¹ÙAßÐªüâ/ÒÓÓÆÙíösçÎùÞvttÈ¦ÚA*ícZõ±ª­ U´h­ U@«h­ U@«h­Z@«·Ý»w¯X±"¨ÄÄDóÊÉÿø-MwÕ¹ÿ;vì÷÷÷×>ñÄ'O½ï`ÜºuëhìZ¿äääÜÑììlÑjVVËÞÉ¯MMM£ªUï¼óZ´0YøàT[,_ÿú×7oÞ¼uëÖoûÛY³f)SE> Õ(ßjµ;vL.®»Ý>ªßAó­·ÞÒdTTZ´0Yxâ'TïÞ½Û9sÏ=ÊTs¿oß¾È^gþì³ÏLãjrròÿøGGÑþýûcccU´téÒºC		Y½zõÑ£G5¹nÝ:ÇÊQ¾ûõ;wë+é»ä¡ôôtå×ÔÔ8r>ÿüó5kÖ|òÉ'ýÌyÒ¤÷îÝ6þüsçÎéËèkGGG8qÂÈ¸ßwü­Y¼x±æY´hÑ~è²ª~9Z¿«úþòË/3¯_¿®LÕþÎU¼3ï¿ÿ¾)óW­Zeò:ä²ÆÆ¦çä/¾øBé???®Gß¡ïë»Úå+vô0.ì«Uñññún&-Ç­¾ùæhÇ>ì²	R»?æh`üb<Ñ¯!¬^zIiÁ8/î¤~øaåKJ755)½~ýzçµ(þüynÙ²E9Û·oWZJ¿öÚk®Çn·»|«~wd ôPõ=±fGGóÁé»*¶mÛ6Ð+¾WNnn®¬O¥µ¿s´0~	RõêUçÌk×®õV/]º¤ôÅ¥b;I]«W¯~ã7LÄ)â²6g_¸pA9+V¬PZòPZêt=Ææ[imiÕìà7­Õ[·nõv¯UóEíÜ¹Ó¥ÔyÇÍÞï¦O¥õm=æh`ü²víZG¤è`ÇÊTsoÚQUÑ;×þÊMÓÒÒ¬V«ò×¬YcòåÊýjÌ ùáê3))Éïf=ÆIæ­¦¤¤8¿çÎSØoßªqVÝ§ÝzÐÒAµÚï1@«ãSeïÙ³ÇÞ·oétó1Uü-[.((PÚ1âÆAOOsD¥ÐSòÐ=û÷ïWftt´§ãÈw³3 Wá²Òyyy)Í¬YÖ7Î?¿jÕ*Gpì0÷áÃµï/¾øâXjÕ4q;7Ë÷s´0ÉÎÎöä¾Ug8àì6ªßØÚØ×å;È+æ®	þúõë.Öïw=æÎA,õ§¡Å.÷­:¢UÇ­g´µj¢sg?>è1@«ã½÷J½ô%s³Åbq¾ÙãÒ¥KQIx/^ti~Íæçç§E:º]rc¢OgZùÚÊT$ª¥mÛ¶méÒ¥Z¾êSO=uúôiGÑ¹sç¹ªhÑ¢Eæ.Þ1ÓêW½7Øèéèir7Ç­ U@«h­ U@«h­Z@«h­Z@«h­øÿ¨iEË²×IEND®B`


½¾(%kÙçó¹kµ´´ôÎ;÷ïß_±bV«ªªü?¿råJólõîæWêêêFÝ%@«áG!y$é`ïÞ½æþ-7lØ`Þ2=ôjZÞÖLBô_íëësl³³³Ó]«qqqÖ=Õ#Èþùk×®9ÚêÐÝwûh`RPg¤(Iö¡_ô1]¼x±¢=iýúõVºÂÇIN2[½zµ¬¦-¼ùæzëÓO?ÕÎ¾§UqðàÁüü|³Í>úÈ!Q­öööÐ·;6Öç¥[×´oö¦Oþûh`æý¥y&V­ U8Ì]ò­ U@«h­ U@«h­Z@«h­Z@«h¢ÿj°·dÉ]»vEæ~Np#ÅÅÅþéééé:ð¡¡!GºRbcc½^ïðððdì­ûÇfòääöcë¬Eõ¥hfVíÔ××O?­µ÷ÞOé¿þõ¯é[¶lQú»ï¾;IV9öHË´h(#îÞ½+»(tSâ'fVîóùé¹¹¹J¿víÚc9(o´h¦IõÁ(qÅVÊg&ÇÄÆÆÊ=t|])J×»yyyú¤Sî_<ölqqqÜÕÕÕwîÜ±qñâÅæÝÝÝýa³pÜqÈ/½ô£&ÑÛÛ«ûíÇµk×¦¥¥éGµÙ×^íæÍLHLLt©®ºñÈ#&÷t¼ZëÔuÈî½yKh~¢¨¨èöíÛGÕòx<:jkgÜÂý£¶£mê·JJJô­ rfÔ³688¸jÕª+%%¥¾¾ÞºòÕ8î[."ZñµªWéééfõüùó111öÒ­¯¯Ïþõ±Þ÷*míï¾ñÆæ]lÔ[ÓÙ¬»V÷ìÙ£Ä¿ù¿±Rªªªb=f[X¶l&;ruÜ/:pË¸Ví,Y²ÄÜ0ÈvãþÄ¸0Öy<ú´==+++¨õ¬½úê«Ä7ß|sW£Ë[î"Z@ïh²Ò,WTT JËÇ³æëúÀÝ»wïÝ»'©Xj	ä*" øXÅ½y×lG_¿7¾eßÏ7ëR >zô())I´ôªrV!ÑÃÍ¼^¯¾àÀ¯ÿxÓØ±ÙÆÆFý¢¹clÿ¡q¿¨·fYYÿÞºr ç·®®Nh¾(íiU1g bÜCä<ê±àfG=kÆ×¯_×²¾«åøøø	^.o¹_hÐªceGU])ö¯+ºµ¹Ö»ã~Ñºé§ÙÄfU¥V¿üòK³ê¸yòfÝÄÕ«Wë]AË6lðo·%Åª>óóóý7ûÅ_«î_´nÃtË¸ÈùüÑdN?á~ce¸Ù¦uyÈAåÌ¨¹xñb­fffÖÖÖ~úé§÷ïßwlW£Ë[î"Z´ªbW)))cÝHtÝÇÁ~Ñ¥ñ§ÿ»aÙ¬.òZËzÕòùóç­wÄ(CF½hípìïûE÷Ü÷:¿.«.?ì!ønuläòåËÆ¬ÿöïß«1+­B@Zýè£ìMLÝºêÿõ³ªJ½Mßåc¥8¢³YëÝ7;n¸dÉ`Í5zUÙmË´Ö[ÝÝÝwïÞã~ÑÜÏ´j3£F«..­ºüDÈÇî­¸ÙQÏÚ3gÞï=sÖëõNðjtyËýR@«0N±«åO>11Õ,Ö^õõõÈLKH«/ùzii©pÿþX«UUU~q¬=1O°Ì­¡¡!³ëÝ7kË0jn´··[ñÊÖ­[ío)è1ñ«~tûöí«eÜ/)÷<x`rÏªÍxÈáÒªËO|ìæ¥Î¦®£® rfÔ³fVÍYE®öF!_.o¹_hF/vý±ZW~=ZcÈÎÎNû×í-KUÆþùç~q¬âXE­)sm;'¸YÓFÆÑwÈÊbs,úuëy¤ÁR!))Éz¦èþ£ã~Ñ~¤Z¶î<xÈ_Ð8UùØSÚ¿¸téÒ rfÔ³f5²¨©©àÕèòû¥Va­ªZ¶lÙ=;xð`~~¾Êqôãë½½½/Ö»úÌ±cÇÿ¢LßJÓÓ¿³`hýôÓOõymÓô.U«V9zÚXwh²(V¯^íÚ5«òáþ£ã~QGgºEêxuÔcËX.­ºüDÈÇ.öïß©mºöæ¾nÖqÖ2¾ýöÛiiiÆÄú¢ý	^îo¹_h&ñ2Û·=zôÈÜcÏÍÍåj´@AAc­:øå/ÉÕh`t<#0*CCCæ:ÑBóp5Z@«VÐê8üïÿþï1Hâþç¢U«_~ùeLLÌÿÿë¿þ+µú'ò'_D2+Z@«hÐ*Z´VÐ*ZE«VÑ* U´VÑ* U´h­ U´V­¢U@«¥Õ'N,^¼866Öçó>­Z¬¬¬ÞÞ^-ìÙ³'''­ZqqqzÝ¹sçªoò£ýhÞ¼yE@«Ò××'jaÓ¦MùMÑ* Õ@¹wï^EEÅÐÐ7­N7o®òöíÛ´´:!zzz^zé¥;wîÐÁÐêDIOOe­À4 ¿¿ÿøñã·nÝ"+Ð*ÃAÎ3gòóó³³³É´VBáÆ^¯wÓ¦MZ½xñbnnn;9VÑ*@ÐH¨öCÉ¬äZE«ASWW·mÛ6G"ÅZE«Î®]»òòòÒÒÒôêÿÇEKKKcc£=åÜ¹säZE«Ë[o½SZZºqãF½Î3G)d@$pæÌääd¶fupp°¬¬¬¾¾A«h5B¹zõêìÙ³íª"W¥=ÌvïÞ-³VWW755ù|¾`dZE«Ê/~ñ´´4G¢RÞyç2"[·nuuuuvv^¼xq~KK2Á4	´V#ææægyÆøì³Ï6449QáZYYYee%7­¢ÕFgqÖ¬Y¶RN<9öì½÷9	?~|Á===fõÊ+¹¹¹-9h­F(/¿ürllì[o½%¹êÕãñ-!ÔÖÖ¶´´ØS:::JJJÈ@«h5ryçwæÏ?oÞ¼äädCDQVVvèÐ!ÊÅ³³³É@«hBV×­[gOÙ¹s'Ñ* U´¡püøñÄÄÄ®®.³zêÔ©ÌÌÌrÐ*ZP8|øpFFÏç+,,bÐ*ZrîÜ9&´VÐ*ZE«VÑ* U´VÑ* U´h­ U´V­¢U@«h­¢UPèééÙ´iSkkë©S§È´VB§¦¦fÁõõõuuu---ä	ZE«¡ÐÖÖ;00`Vûûû½^ogg'9VÑ*@Ð,_¾|ß¾öÖÖÖêêjr­¢U ñù|/^´§:t¨¤¤@«h hÊËËÛÚÚì)MMMµµµäZE«AÓÓÓ|øða³ÚÙÙ©Õ3gÎ3h­B|||~~¾Ïçóz½"OÐ*Zý?~êÔ©ÁÁAr¢sçÎmÛ¶mÓ¦M===h­@èlÜ¸199¹®®®±±1##£ºº:+h¢CI¥VÍê­[·¶¢U´ÑMMMM~~¾õïÓò0»ººZZZÚÚÚÝ±µµµÛ¶msìaqq1ZE«ðÑ¹»téùQ³`ÁÆÆFM*=ËËËå×ifVý=/_©ÃT"11±½½ý±ïUYY<ê8ÙÙÙh­ÎtN<éõzçÌ3wîÜþðä	D*Üí)2Ðºuë¦Ó1ÖÕÕ©5«ÇY­YúúziÞ¢HZ?mµ:ËhÄ7æÍ÷ÜsÏ]½zU«»ví+DU±é¾û³N§cTj=Â44	÷	vïÞmÉ>2ûM«1®ÄÆÆ¢U2èüùóí)2«®¯èoSXXQSScÕ !@¬!õ!íÐÒÙÙ9Í´ªØÔÑÂVqa$Ã¥YåÏçS á,lºiÀ-òM°=vÔÏïÜ¹399YõSýÉûûûËÊÊ"°Bd¢GUUrOÅ^÷-!dfÂM`IËª7´¶¶FÈî)V¨±Ï³§B«CCCkÖ¬A« ^Á¢ÿìÙ³Õ¯×ko¤ ¹fff:f_±JUAT1ÅP~~>£þ@,Dõ RUÌüõTÌê`³³³§kç(Ðê+WRRRbbbx¶þìØ±cÎ9ö¶/¼ðBRRÒ¨k¥UG¢þáÌ¹=.§Nr<Ô3>>ÂaAêêjÏ'¡::ØìÜ¹Ó;´TRR¢lÒcÔè2bÕÏàñh5--ÍÑ^ÉãñÜ¿­áW^Y³ª`ÒvÞ¼yºGý¤êûþÍCjjj×ÀÊÊJGbQQQÄ÷6=Æ O5°Ý»w«¦;j«ÕÃhÍðøµjâÔ;wî¨ÄÔëéÓ§µZXXVÁ³J«yyy¯¿þºïÕ²²²úúzkõÜ¹s6àþÕúÓÙoEj!##cRQ+ÈsóHÒ¿ÚªÝSÍU50í¹.ãÇs#W«ZPT±víZ9nCÈrEõýåË+BmnnV(mÿ"_ÿú×sçÎê©§¬[MMMª?©DlÚQQµ¸¸Ø¦µ«xZ]¸p¡èõë×­ûÀ©©©hBCgYõM½CzT°qãFE»víSÿò/ÿréÒ¥ú|ç;ß!Êl233÷Ö­[BwO÷³áBco¼:¢m­>zôHU³üþûï<Õà._¾V!dT®¬¬,//WÈ5Va°æqõÀÀJÉÅ©ÿ÷OÎL6uuuºJ­õâÅ*ª*X|¹jEcVÿ#mÜ¨ÿ×4ë)4M´J¿U/*§d®®.-û|>îS¹þÍ§U/©©©!s&]ùùùÒá¶mÛä'`-¥+µÄ<©7fð8ÆÐÑVÑ*Zþqªj/VTè4443.øÔØØÈpSÃDYø7¼³£>[ånP$j1!H¢Sí)ÇW@@Î¸PUUexcÉÉÉ4ò|twcÖ¿%ðX]Ýà1kÕØ®UÆÐ¨¨¨p©tîÜ9ÏGÎ¸ øC¥³rISuuuÌêD«ª9¢ÕÉ¾1+kû¬y¶¹ZµóàÁ5kÖH«~ú)ZhnnvLüÔÐÐà?Ðø£êHKKKkk+£Q­rc­~ááa:ØÀD¯Å¬ªSK¿rssí,¦Ó_RRbo	ÌY´êtê±cÇx¶Ag¹©©IEÏçSYSaRéïï¯©©ÉÏÏWÎ1ÞïÁY´:~%Õ¹Ð*D87nÜÈÌÌ¬®®>uêÜ6êì4S­Uÿ9ÌåÔ¢UpêêêäTÊôK¢O«ô[(¥  @qª=eß¾YÉ@«N"áãD¸qãF ãëêè|;;;Ñ*<­Îr&K0Ù=ö­·Þ*--U¡i®^½jöM¯Zhkk[¾|ynnnYYÙ¡CÂî¤èâØÕÕm¦ã5Ï9ãòá)»	 æaFkÕþ0Õ_«JGGÇìÙ³çÏ_TT8oÞ¼©ëfppPÅ´dã2áöM×¿Ù7ìsçÎ=pà@xw£¾¾^BÝ·oßÅåWýÊîÝ»Ã¸ýèâ¨³£ÜhooaÓ¦M,ptv*¥ù£yZ¶£ÕñÙ¼y³<úî»ïÞaõêÕZÝºu+ZIB<úúë¯[)ØO>9ÙÑÂ Ñ>OãX&S1­¦ß^~ùeím÷M¥¿B±+¥§§GûÆÞ'---*ûÒ®´ªÓ*¡ýv·êX:5ºrLLUYÜ~î­Çã1ÓÛïÇÇÇ£U$vìØ!:D+9úÑ+,Pª0K¦uô·¿ýíÍ7CøQmMNµTjLæ¸ûªøÓ?ýSíIii©=kßÂ°¶¶¶ê¸Æáµ¥OeGÇ S:Gq»¦sù%&&¾øâöëDÙè? ÕÑµªZÞÐÐÐ***´VaÐÅ¦*¿#Qå×¶mÛ¬UYMljjR&IÙÙÙöòkP?ª"R[³§§ÖêÞ½gÏýÔSOIù-3gÎÆ­SU`Ü¬àÆýY Ñj]]#QVµªí;,))ÀÅÅÅºNûÔ~inlèQñ«_ýJuq2gZ6ø?[Ý¾;ZIÂ<¼´ßó¼tévöìY+%##Ã~VÖÑe¹zõê¯FÚ=ýôÓ)úQÿAG¤jkUWæ~ðE¥ª~¢¹¹YÚ±'OjßÜ.I¨Úa)¡ªªJÅ±Âkg·f+W®ØñðÞ¾qãrL©ÊíÂÂBi5*,©*£½µ²B;ÅèÜYÏôëUUÒåañ2gZý÷ÒÓÓ=#¨tèéé¡L**¶öYã*©kþüùYYYÖ»þÞÈ©ßýîwu¡Z)rÞ+ÿÅÚÚÚM6ÙSöíÛ'eý©¬¨¤¢Ô·ª¾ðÂ.[Vi«OZûf¦£q¿O¨ÀQ4­2½B"Ø1ÞÄÑn¨ôWe¢¼¼ä¢ùHÃ4ÖÉbu.t¦xç%QëòÓÙÑÙÔ¡Ô)ÖÉh~«H¥ÒÔ¨WÅ*=íá Êµkµ¯¯OZuÌä*-åääþDë­PU¾1«»víJHH0ËºJî¹ÙDËîq¤ª¢ßÚãà±ö§¦¦FÑwÃxû×]´åoË**<­«««¯¯wL28eZµfÖÎTTTèúüÞ÷¾§ßÖÖÆÿ­¢UPN<¹cÇû½_Kl*¿¬L7oÞV|òIûcË§zjÉ%AýDmVaJéÜPJê2¨½ÍÔ7dýý×w³QÚu>000IÙîÝ»U«T;Ñ±ËXQ¿N½×Wö_|òNèÏþó©©A´jÕóá  Q¼âõz¥+VYd8ÑzW2ÖU:n3"þå_þ¥¼¼üÕW_Ý²eã­¢¢"]¦Å¯|?þüg6mJÏ6,===ãF«þÊ÷ ½¤¤Äþäuâ(z^°`öÊwAAÍY]0ýj¬Ë/77÷§?ý©ª&*|© U·á Ì1£ÁpðØYÊOÅÅÅ?ûÙÏMÎ7OêÂåÔ?ÿó?v_FFÆÆ[[[óóóµqG)ÙèWæÎ«×¼¼¼Ç~2ÏVU­© ÖÆ­VÄ [ótê?ÒØØ¨UçHíâKí¶¬£¶ßdmm­C¢Ü¦æò3ß/¼ðÝï~Wf­ªªòà2<Ìh­r¢«W¯>÷ÜsO?ýtzzú[o½åx÷wÞuôÿ·¬øCE¤%$]²õ¨M:ªÛ(×4rÑöò&$$$%%©n*7Ò3ç«>»öÎ²µME¼#%Ý ÂÙQÇGäÊ|Ö«fF­Yµ5±ªG46¡U´<¡â)>>^yê©§b¾òÊ+¯¿þúO<æïÅêêjG?#ÚBwI¥ä#G¤U¯×«©öá?ü¡äjïë"QÞhï©X¶¤¤ÄXË¬.]'õIÝ®9½Z®£Ój·©g&:ÊCÇ]¥0Ö¦6¢ëMUmóÜ¹sö6t0­´úðáCkÒò¬¬¬Y³f)@«Étvv*ªþîïþnÎ9VWYJÁâ/¾è±!èì&@+8þë¿þkëI­l'­Ú¥.³ZmÇbÁÎöÎöE_ý±©ªl-<¯´ÅÊÊJÍ¦¼VåC¡ÕÛo¿­¸Ù:|é¦Ò¸ttt(¬t$*eçÎÁnÊ¿zÔÒÒ¢2yjUN5ôO¶,åää UX¤«ëêû·«Kò¿Éty;ºÁÈ+2VÈûðÿø¦q~Ë÷)ËÈ£û~XÝ7¥bZ.´^Õ9R;éèÕzêÔ)X	à$`ý­òòò¾BYa÷ºüªÛ¿¿þé²©	ñzô;QV+?í.Ô²R7íÁ¿z¤Ó¤M&OC­6JZPÍWÕÞ0D2·nÝÒµjtiii¿øÅ/dMKRb8Ç·¤"ÅætÀÜ¯ÿ÷¿ÿ½éÒj­óæÍúé§_xáÒÒRû'Ï=;®VÍ>OG!SjÇì÷«ý£ÅÄú~øáVÊ¥Kô/Þ±cGkk«6âØ¾TjTqª¢nîÕ©¿b¤Ð«®e¾*=&ÓT)óChì_=Ò6Ñê´Õªþ¢CCCz5wÑ*D2º$H«É=ÔÕÕYw5_~ùågyÆÿ2Ô¥L_ÏÎÎÈTt*påQÊo~óýqÖ¯_¯ òäÉVú÷¾÷½QwÆÅß²¾¼èkÞ¿ÉÒüùóãââ_×g~ð(Rwå_ÀGËÑUdÎ¼xñ¢^ui©ÒV^^®ËFU]9:õ¡Í`cÆe´§455qxzj5==Ýº÷«BÁhõùçG«±ØGT88wî÷ïßãÆW^yEbsðÖÜ¼¼<¾DE¥«öM/¾øâøCÅO>ù¤ÿxÁ¢ãR½!EKÿüÏÿìÿ?Rè,­JÉúQC;wÚpA¾TîÙ«5«RúûûõVOOÏDúë¨n$³ÊÖ]]]UUUr¶£ÓD«wïÞMMMU©ôÆol,A$#m(nPX¦â©½½ýÛßþvBBkPQÅÞ½ßÊ5sªSBØØX~TõQ3iâÅÐ-Y¹ZÓÛk(>ó¼VQ¢_QeÂ49VÈmmm*»åTk ÷ÌçHT¨êx&:tR*++õ+ÌÛ:mµJÒÀBæ0£ñI$úW477+¤kmm0ÕÕh¬£-ËÓ·C1ÏV¿óï*HUÄâøz;Óp7É©ò¨"c)üõ×_×¯ÛçPµcÝºufðØ4íµê?+­S#@«h¦9R±BÙÙÙ#§Ê¦Í¨¹KHá©ÄfFeRú­oË~uÇôvÚ+ÇÚ g+ Ö>gee½óÎ;a©«ÅÇÇûßfÜ´4.ÉÉÑõdççç£UôSí_cc£ÕÕ	Éaß²²²`§ö5þó+xõËz»¯FÆå§Ãh$£³ãõzíM<­Mww·c-¨ÊV!*hÁbºº?+½qã#Å»Ñ&cz;KùãöUÉ^RR"%WTTÐwê1l²³³uì7­JZZ<zéÒ%»V¹¢Uÿùèª~Tã:Úa±uLo÷ÕH#díc°'ªÑ>|XÑ¯d|;(ÓªS­©â¢9ÕÑùïøñãþ£ìúÓÒÒbD«Pdßûôv²©×ªª*¾.¹¹¹ö0cAp¢I«IIIòè'MÍBjj*Z¨@tX__oÕSºnoÑÔÔ¤RÑaff¦;íÓÛmÚ´É½ÿhCCãÉ+ãÛDV÷ìÙã?yggg°Û9ú´*æ±±±yyyGA«0erEêJLLPu¶¶¶þÝ[·nE"aÀiÕñäñí¢O«¢··WåÇãU*8p TTTìÚµK[·nµF@«0eÈÑÞ¿¾££Ã>§ûÝï~7!!A®/Ç Õ°2<<lf3SËýìg?[øMyæÇØ4Qä·nÝºòòr9Oñq¸ÝÜÜÜöööïÿûIII©©©Ú´ibñpÒS­ÕÛ·o01t~³ÜÝÝ½é¬]»Öj¨£¿¿?;;»²²²««k÷îÝ>Ï½-RPhñ¡ÊOúS«?®iS«õõõfªHU÷ìÙc%ÖØþÜiLYY¢3gS[Â_ýþ³G)*óóóUa#&Z]µj£Ò3gSÝ`jjêÃÍMà±£UønãÆþ"mÛ¶ÕÕÕÙSdîäääq;Ö^øáAÍ70èÌ<w¦¥Xcc£+YÁ"N«¦_þZnoo×²ãã/Bì»bÅ?þXz­¨¨@«0½µê0Ü¨ñeÈÈ7½ÃÏ¦MüÇl2ºÕ[úÇWVVÆÄÄè¬ÐYÏ>ûlÈÑíäôÔÜÜì½¥¥Å1éä÷îÝú9½N¸"K«för³¬øÒ>xah;v,--M[ðz½'N@«0Q°å11???7lôWRØzèÐ!	Ôáôd©ÎÎNUgÏ­ÿ 1´1þü¼¼¼`W__¾|¹c¤§0FþÂè('û)÷TÛP^555éô)oÂ¬UDVûúúÁ`É¦±±Q¥þ*¬UR½çkGGGUU$×ÐÐ0êHcó»o½õ<*ZÝvO<)Ñ;nôì?ÒS£IÔIæU¼¾¹²©~QGôÄOØÏQ[[[FFÆÄ'´´:V @úûû«««>âTiïßÿýßkjjTpk9iéÂlgõgåWd,û¡sçÎÚæ¨#=1Ïì>S,®v2ò§««KÂVÜÿÉ'(p/..VÈª1+&Q«40ZiO]]]vv¶â¼CI®¦fòpùÉº3üÎ;ïH!ÔnRTR­úÏ±öhRâ×ïÞ½[«Prò²+33ÓÌô§Ú~HY¡:5°eUUUxïÛZu¡öD*uL#3MRøå@á²KK~±±±ßúÖ·LTÏ=÷bVyN5m|Ü±±ôzÁ62R@ìõzÍ]ßS§NiY)­­­¦µéEdø*¼MíVÜÃ;ÇL×ÒÀÔäÛßß¿`Áæææ[#üøÇ?Vx:wîUCÇ|×ÖÖNYðí~·¨¨H; ×øøxíLà°÷»ß=ñÄõW¥oõôô,_¾WL___¯Íª¢ H¦Vh¦æ¨°¦­/ÊÉ#;wîðáÃ;vì¸zõªû?.ßq-dÇ»»wïvo-5¨¸P@©0òSeEr°SWWz¡¦¦fãÆª|üÓ?ýtÎ9©©©Ê+-w@@«h <(ª+,,´Ç=mmmSÓ3dÌP²®[·NÑB¤ídgg§<jOQ¥A²tö.¶¦:CÇxæÌ	UÝ²eKlllVVª¤Z¢ååå¦°JsåVü÷úë¯<Ù³gëµ´´4vØL6gPÛY!#EMMMDûi«­ê©­		dSYÖ|F1®â]ìÛßþöo¾P­Dy_PP`M5£eÙtÇZÖëO>¹hÑ¢©Ù%ÓÌgÔÖ@öÖÂòÐÔ ±iÇTùwª;F:´§H¨ªXjôïbk¢pi5??ß±5ÿa(Ð*@4aú·=ÖJÑ²R80ÙB1IB£Èúþ-í]`';;;[µªªªÌÌÌââbfÀÚmÕT¬Ñ-dSí¶ÿ.¶_+iÙû¤*U ËþVa"ñ¼õÖ[7nyàV ÒJX¯xKyÚÜÜüÌ3Ï8öY«KÌ$¡ã­¬¬4*Õ«ò¡ººÚþþþ~EÕv;wN)Á9¤¿ó;ï¼SZZZSSÈ úE»&õEÇcifÄAÆhXfMý»ØZttth¡­­M§ÞLïh­~­Òo"ÓøÓ4gÎs5(ÝN´Íq;G_üâiiiD¥HEê`í¢RÕDãð¥öÜÜk³§§GË?<ªÿrBBÂ/¼ ¥(ëþÿº:Ý:SîRéX´ú®öÖ!`ÿ.¶ö		:;;õsKJJôõ`srê/z­Ú©úk~«ðXxî¹çdS«sbV]ãöq àLq=E±#%(ÔS©­"Þíi·%ß]»v)%ØÑÂ#ìH,**òïB#É¦ú?J<ã:Õ<µMª¶Ï¨#§h£6AÒ¾ýæ7¿©¯¯WmÀlÓz2¦­¢R		EuÓ9ÇôÃ¡ZÍ7Ë£ï¾ûî½V¯^­Õ­[·¢UzT4;¾Qx÷ü$¨¨h¶O©öÕtÂ[Ëa^¯·²²²¸¸ØÞPUpÊ(zÕ²EaÐäÔÚÇüqú-íaÈÃ_¹r¥°°ÐçóéH¥áüü|íÿÙ³gçÎëè÷©µ¦¦ÆeSþfL$ýgögV;jFÙèÜ x¬.¶f8½%û¨[_k®»¹£Î­Çãq²of]E«0ÅtIµ4óòË/µiÀ¡7¼öèÃJsÙÔRõ+Rc@"E¨yyyJÿÞ÷¾÷£ýÈ;ºa2n31ù¬çbÝºumÈ"×w­Lt(Óè =SÚemMNÕ¦ô-ÇãUÓÛgâY±sçN3Î¾ä*YÒ7W§RE½bnSO<¨U]ëCCC<¨¨¨Ðj\ZÇ­:&Qil´ê([±ÚÚÚÂ¾·Ú7G¨ØíîÌø^EQ4d ±äåCn±¥½íÎ31·|%ZEçÄ¾²¦lª8UõÅ1FÐ&8Í¸ßÁãöÍ=~ü¸ÿIÑNðhf´V7lØàÿluûöíh¦ÅF*­òúõ×_QÔFtIo¹¹¹F070Ã~ßUå¾åQÑá¨Wî¡%û #%@"¶L)êò¤vÑ¢E:S÷î5·ë¯ %W«O­ÿÊVkÞä@hsUIÊÎÎvªhkü7Ñjp¨îA%þ3t°ÇJmEB*²cccuµÜËSJ¨Aáã$=ËTÜæx«5²oüolÊÁÞëÃt3µçÍî_~Ùì¯W½nà¸·æûæJ½ö¾°ÚéË­Òo¢«W¯îØ±CBðÁÐ%EÂÖ`­JB£î³ÀÁµÿýÏHC*ª­­µzÁ*xu<¥>yòäOÂpµæÕIQQc¿û-¡Ò7W?­3¥ÃlkkÓigUÑ*Z:qª°233åGðj§¬¬ÌÞPÖò.¡öðÔ¬aÆlÒQLðöì¨¸´æÞ«j>ææfmYÔFOXUCÒÉZ·n3¡Õ¹páBNNþäfüü|´¸xô Ýo5+TÝ666*NÕßm²¨-((°nÒæååM$¦èééúYW¼J¨¥7IÑÌKø×eÓmAb&è©Ójww·cp%-¨~VÂÎ¾û	µ¶¶Nv*[O¦O<9þüg6ºòJ½BLûsM©]feTt­¦¥¥É£.]²k~«ÑàÉÞðX±²þÝ¦án(X4Ó«)Î¤ÖËþ¨òá?i¹Bð©iÐjèZ5qªYÐoxx1¢QkÈÌÌ·_é¨V<µµµ]]]Û¶m3óOö!Hä/~â'Ìd	VpV!Òµ$8qÂØÔ,¤¦¦¢U(EeDll¬ãf©þéã?*EEEvö÷÷'&&NêÀCFä«V­zúé§Ï?¯_×ªl*¯s¢@«öìñ¢³³­D/óìC	6iÁWVÉÑ«®®Î¢Ó0RYYi¦FX·n]FFBä+VäææjO.àñhUôööêÚõx<ªáz½ÞÐ`¬+k¦¥¥É©!L±÷ÕH;gÿ1ÿü':/Ö`÷_QSS³lÙ2E®Üþ¨Ñ*ýV¦M¤þä'?	vH;¦÷§]´ªOjââbÇÐÐ===42Z)Å<ÑliiQ©åÉcÙBaIÔzª¶¹¹sÑ¡Uó0uýúõöZ=X¬¨¨ÈÎÎÞ6mÚ43[%HäeeeL'Q¦U±víZ´ÂL0YZD·oßn­ZVµpôèQs7­Z¨VÅ±cÇì£U@«Á!ÆÆÆZ«2«×ëeL`@«t°@«Q­Uû4pþðlÐj(TcFÃþ´­Zå&0Z:­Îrg«V¶já¯U­ZÍ7Ë£ï¾ûî½V¯^­Õ­[·¢U@«AãñxäQÇýaFY´ºV<xPQQ¡Õ¸¸8´h5h6lØàÿluûöíhÐj(tvv¦§§FÈÈÈèéé¡ Uú­<V­*NÍÊÊº|ù2Zg```ß¾W®7 Bµjú­­Âô£¿¿¿½½ãÆ*ÉhgÓ¦MÉÉÉå#ÄÇÇëÌ'ZýøãMKà÷î£U:t(11±²²²©©)??¿°°ðÆdKô¢5##ãÜ¹sfõâÅ»wï&g â´Êà0ýðz½*­ªª*8äLô²|ùrD;::JJJÈ8­21L?Tþ*Nµ§(TMNNæVpôâóù¡ÚS´MÎ@ÄiÀ0ýØ¸qccc£#1##v.Q­îÜ¹Ó¢U¢U­­Y³­BôÒÙÙYPP`MÏ9C´íçÔëõªø3«ZX°`AGG9§UÕßSRRóØðl¢é3??¿¦¦Æ4S:~ü¸Ïçkii!g¢ITu£ü´à^"E«iiiöJçþýûh¢	µ¬¬,111##CQNkk+y2=*L§FPaBn@jÕÄ©wîÜQÑ£×Ó§Okµ°°­Âô(Èj­j¡¦¦fíÚµ*¸	h5D.$zýúuë>pjj*Z´4=òz½fùý÷ß÷x<4D0Zi®Uú­ZE«£ÕY®Ðd	ÐjCûk1­ÂæÍåÑwß÷Þ«W¯ÖêÖ­[Ñ* Õ ñx<iÌµV­¨Õ¡¡¡TTTh5..­Z6ø?[Ý¾;Z´ééé222zzzè`h~«h`zhõÚµk^¯iÌ­ysk´F¨'Nà&0 Õj5%%Å1Z´"G1ÃAÜ¿­ZÃM`f°´­ÆM ªéêêjiiikk»xñ"¹SªUú­ÀtâÖ­[EEE>¯±±±ººZeÎ¾ûÈxZZ³fZh¤ªªª²²rppÐ¬>|811ñÊ+äLVuµ¥¤¤0L,XpãÆ,ÛÞÞNÎÀiÕ>ÁãñLR«`´êõzMMMëÖ­#s`´jâÔ;wîèZÔëéÓ§µZXXV INN>~ü¸=EÚîÝ»ÉR­j¡¦¦fíÚµÜèeãÆÙÙÙÆ¬*s>È"­.ÐHôúõëÖàÔÔT´QJ[[Û¼^¯"×âââò¦N«=ÒÅgßÿÇxùòe´QlJA«ô[´ÎÁíORe¾»'N,^¼866Öçó>­Zý¿÷ÂàYYY½½½ZØ³gÄ¬?üáÿß7nÑ*LO­*$5ñññ¹Ù§×êêjÿ-KØEZ½~ýº5¸IqË-!;µ¯¯oÕªUfûç¾IOOÑ*LO­ZýVÃ8_Í½÷***x¶! +dÛ¶mµµµª!ZÖ­]³zóæÍ+WÞ¾ÀW®ÎÎ^¾|¹ÌÚÜÜØÖÖF¶@ÔhuõêÕög¨¯¾úªÂV¥vX±ÅK/½tçÎ:Ø@hÕ××[«çÎY,¢C«7o¶·~íµ×ìäÈ`7nßZ LNNvL<R]]­ÈÌ(Ðª¸æ³Ï>3D§öööj!##á `*kâ2¢@«±±±Òçðð°W®J	­ß*Z	"­êâ¶§äççïÛ·(ÐªéZ3<qê¯ýk¥?|ø­Âc¡­­-##ÃUJ]]]aaáàà 9Q Uó(ôìÙ³ü±ýiè®]»Á£YíüêW¿Z²dÉ¢E~ö³9ÑZ=zô¨½Qcc£½ÏÌöíÛÑ*<.ÌÄ#999ºSRRT=vbbâÍ7ÉP­­[·Æ°zõj+1!!á½÷Þcx¼TTTÈ©;vì0«¿ÿýïcbb$Zr"W«LbÓ%KØS~üãË¬ä U´A£ëDµ§ìÞ½öìÙä U´AþôÓOÛS-ZôÄO3VÑ*ÍoûÛY³fåääfJ+V¬ÐêúõëÉ@«hBaË-sçÎ5MÓgÏí¸'VÑ*ÍÕ«WûúúÈ­^»vÍëõZó[³£U@«AcÆÜ·lj`ðB@«¡OE~âÄ	nZ¨VSRRÆ­Z#GH«÷ïßG«VÃpØM­BÌhÐd	Ð*ýVÐ*À´Ñê'222bGX¸páùóçÑ* ÕPèììôo²ÔÝÝV­Øoµ¡¡axxøÑ£GZÐjjj*Z´bGl­­ÖÕÕ=AZMKKC«VfÿþýþÏV8V­ÂÙ³g.ñxâââ´0yÃî£UþZ¥ß* U´Zµû2Ô> ÕjÕO¡ö­r­¢UÆZøðaFFYÎÊÊ5kVzz:Z´rªi ÔØØh5YÊÉÉA«V¿¡íÑª×ëMJJzðà´J%@«!jUÒ«¹V­HzzºuïwýúõF«Ï?ÿ<Z´4wïÞMMMUÌúÆoÌÌL,Z¥ZZ¥ß* Uú­DVé·h~«§Uú­Z¥ß*@äi6VÑ*@DjõÂ999ñññ¦Ip~~>Z´ÝÝÝV%£U-£U@«A&^ºtÉ®UE®hÐjÐ8Õ,H«ÃÃÃ_Ñ* ÕàHJJGO8aljRSSÑ* Õ Ù³gÏ,?:;;Ñ* ÕPèííÍÈÈðx<±±±^¯÷À´´J¿UÇªÕôôô¬¬¬Ë/£U@«áÁhÐj´úñÇK«÷îÝF«V'ÚoÕýV­xØ¦1´JK`´0=´:Ö³Õªª*´0C¸uëVfffbbbAAÁ¾ûÈ@«¡?[u8ÕZ®¨¨@«ÓÁÁÁÂÂÂòòòþþ~­vtt$''ë´Êæqqqo¿ýö½÷îß¿ÿÞï¥¤¤õööJ«ª·¢UiO»´*¹Z)]]]^¯´4ñññá M=z¤ð6	F«ICCÃ¶mÛÒê7È@«Ááñx¤ÏÆÆFE«wïÞÕV¿~þùçaÏ­D&úã·´´ØS¹&''ßºuÌ´[¶lño²ôÙgïÿûh`ÚsøðárÅJY·n]QQ9h58`M¶ûvs+xéÒ¥´!È£2ksss[[[yyynnîÅÉ@«ô[Qá"­ÖÖÖ¶¶¶òTÐ*Z­^¸p!'''>>Þ°V­Bww·câ-£U@«A&^ºtÉ®Õðö«A«0S´jâT³ ­3ß* ÕIJJGO8aljRSSÑ* Õ Ù³gÿphÐj(ôööZÃAx½ÞÐÐ*ýV"L«·oßÎÉÉA«V ¾¾>!!aÖ¬YIIIöì±i	h58V­Zåh¦tæÌãT¡´h5¸~5÷îÕr»MäáÂ­Z3xøð¡ðB,Zzp%»DVûúúÐ* Õðh6VÃ£Õ°Ü6óá UqZu!4Å>zôhñâÅV=ºãlÚ´Éãñp`ºi5ÆØØØ´úþûïðÁVëêêþ&O=õ¶ÌYé¦Õ°sýúõüü|3¯7­Nòòò£GÛËhÐê×!? õXV­ÇµD«VÑ* Uæ[@«h­ZE«h­¢U@«hÐ*Z@«h­ZE«h­¢U@«hÐ*Z@«h­ZE«h­¢U@«hÐ*Z@«h­ZE«VÑ*ZE«VÑ* U´V`N:URR¼`Á+W®'h­B¿lÚÜÜ|ëÖ­7nÔ××gdd3h­Muuucc£=¥²²Òh¢  àÜ¹sö®®®²²2r­¢U ),,ÔßÞ²÷îòòrr­¢U ijjRl:88hVµ øµµµ@«h hôÇGKJJ:;;;::´¼|ùrË²V 8nÝºÕÜÜ¬µ¼¼*N@«hÐ*Z´VÐ*ZE«VÑ* U´VÑ* U´h­ U´V­¢U@«h­r­¢U@«h­ U´h­ZE«h­¢U@«¥Õ/"^x!µú?üaÞ¼y±áfÎ9±3¹sçÎÌc×ëðgì©ç²ç²ç²ö?­Z®]»6kÖ¬óçÏÏÌÃ/((hllÇ¾eËoûÛ3óØUCÕeÿßÿýß3óð_~ùåüä'3óØwîÜ03ýÁºì<85?VÑ*ZE«h­¢U´VÑ*ZE«h­¢U´VÑ*ZE«h­¢U´VÑ*ZE«h­¢U´VÑ*ZE«hu¸÷î?üÃ?|ùå3óðwíÚõ»ßýnfû3g¶mÛ63ýþýûºìoÜ¸13¿££ã?ÿó?gæ±ÿþ÷¿ß¼yóÌ<öGé²W(V¢´VÃÁþùØØØ¼¼¼cÇÍ¨c?qâÄâÅuì>ïôéÓ3íÔß¹s'==¦µN´×ë5ü#G8éüÙ)çÑjQ.úé§ZèííMIIQÇ¥£ÖÂ=rrrfÔ±ë¯;kÖ»ø+**víÚ¥­[·¾ñÆtþìóhu²8xð êq3öðãââfÔñ^¾|y°*SµððáÃ·ÍØ>Ãÿì¥éÙ£GôgS=nfæ@__ßªU«fàÏÀ666vÔeN:vÊy´fº»»SSSgàß»w¯¢¢bhhv&c-<N:vÊy´ÿÁ¥"?CýæÍ+W®¼ûöÌ<ï3°UòðáÃ¯GnÏÌzäÕêú³»05åüV³²².ðõHK¹^ziFOOùÎ;3öìÏÀvÅü±ôªÀÎr­Ó§Oçåå©þ²lÙ²VKOOev&pìØ±´´´¯×«"Îr­Dví,@«h­Z@«h­Z@«hÐ*Z@«0c®oâñx222Þï½Èßç0n°»»»¨¨È3ÂâÅ?üðÃßyûFÂcmÍq¤¥¥ðÁaÿÅIõlÔçåå)Ñ>×Øàà`LLÏçÊ+Ð*L[­Z¬]»vhõ£>ò?üõë×wûúúÑê¨IXêÁKÈgdÔ«©D]VJ»RÞ÷]´VÂPbÅëõÎHJJÒñ=ztxÿøÿÐjbbâ$ a×êÔTYìé=Ú°aVÓÓÓ§>CB>öQ¿xúôi%Z)eeeJ¼Ñ*Z[¡fnôY«|òIVVVll¬Ïç;xð ýÛ·oOIIY¸p¡)aÇ¿ýÛ¿%$$,]ºÔ;þyQQÒãââ|ñÅîéÞ»wo^^>³ÿ~Ç¡éôsz÷ùç¿páÄÇÇëc7o~ðà¦ë×¯kO´?fVáëþ[Ê¥ÔÔÔ´´´]»vÙwÞkÜk#þËä$ºe÷ëD«ÚZ§;ÀczWÕ;]555rù¨gdâ¦ÄÄÄÜ»wOËCCCZVJ 'hÔ<Û­ÂÐª¯-[¶(%77×¤tww;Ê2«péìì´¸U¾¿öÚkîÛ1Ï·,,Yânÿ¡Ï>ûÌ±YÅc9F%£¬Y³Æ¼+%466Zòµ×ì)ù¸¿µgÏ±lÈÖLî¹l$­ºd¾Ë]¢Õ_þòZÍÌÌátx,ª-ùß÷ÿÖÄMÎVºü§es¯B) qµê²oVakÕ)Ó|`Ù²eZ½´|ìØ1-¿ñÆæ­ÂÂB­644h¹¾¾Þ¿¬1o¸oGñVí>sO·ÿ	-×ÖÖª¬×«õCö½ùæÃÃÃb!¸µk×*"1×ÖÕÕ)jq¿Ý§²oÐå·òóóµºzõj-ëuÔº5î¹ldÔ;ùï¿ÿþ¸'1Ýë:Q,Óà±«KÞåËýníØÄÍ¯òòr-WTThÙ~ÏÃå«U´3H«>ÏøÇÕ·bccµzóæMs»Ì¿¬±Ñe;&Ï.]ª äîÝ»îéö2øð¡õªe	Òþ±qiÐÇ>øàóU¬r¬o9sFA­)²Ç;êoÊikªqlÐeköÜsÙÿªW¶XNuÏ|÷Ýõ:ÑÖ·mß¾Ýñn§;Àc1W×¸÷W&~hªÅpÿþ³`n8ÆÕªË¾ZéØYiiiVØa/Ýì¨´²¿%¡jYÎs/ë]¶£RLÖ,++óz½Jé¥ÜÓÒêXÅñ¨è($WÈ%Zï½÷LxÙjÝ9÷·æ¾Ç9¢wtrÉ|-»ÈlÜwCûE­ê²tÿ­xõÕWõVUUu;ðd_6W ãFÝ7@«0ýµj¸sçNFFRÖ¬YcRÌã¥îînÿï.^¼Ø8Xeß¸w&]¶c188ho3Vºÿ6ûM`«÷EZ5Înoo7-<¨U«ÑùÖL$jJjÕ$d¯µjnfÇuuuuþÆwkî±/÷ôôÈ©Vï @2ßeËÑjh¿èo¸téÛlëLüÐ¬æVÃ+GmÆýw~öÙgºxÞ|óMÿgçî×< UæZýúÄçþõ7Û%¬Þü±ë.íâÏÂjJ:VºãæÁÞÞÞ ´úþûïûß	·:2Z·W¬X¡ÕZ1MM0äò[~úéXgÜûFìËÊ¢Q?ãù.[VCûE&Kõõõ£¸û¶Mªg¥rÆú´3H«_ÿ±Ç÷¿ÿ³ºk×.YS%"ÕÇ­gBÁDzzºÞ½råý×¨k;*Ô***L7;æa­KºÓ­E·GßþðÃÍÄ%KìÑ>§¥¥)ÝH½¯¯ÏtæQævM|ûí·Çý-Õ?tÈ­Ipkî±/¢T¸Ä±¶<­öí¨rj:ØÜ¿Ô32ñC³Gr¤=1tùòeW¿cUÉ@«NLÓAÓÜ;MII![­"HGldÝ©@«ÁqçÎ+W&$$x<Å©rªÕÃ­Z@«h­Z@«h­Z@«hÐ*Z6þ&T7JU§IEND®B`
