# Supplementary figures and images for: Feasibility and benefits of group-based exercise in residential aged care adults: a pilot study for the GrACE programme
Source: PeerJ. 2016 May 18;4:e2018. doi: 10.7717/peerj.2018 (PMC4878364; doi:10.7717/peerj.2018)

## Slide 1
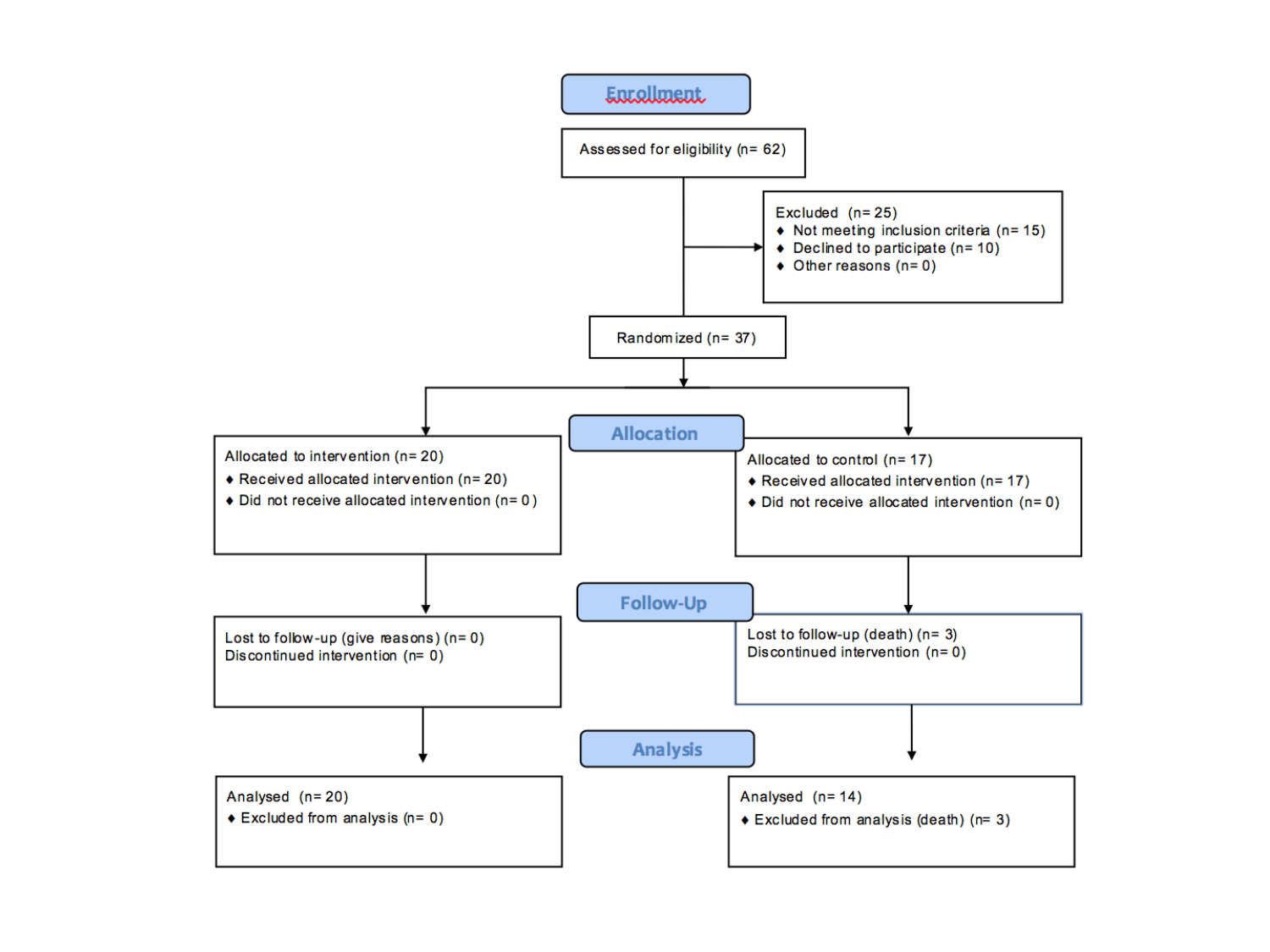

Supplement: Supplemental Information 8 [file peerj-04-2018-s008.pptx]
